# The role of analytical reasoning and source credibility on the evaluation of real and fake full-length news articles

**DOI:** 10.1186/s41235-021-00292-3

**Published:** 2021-03-31

**Authors:** Didem Pehlivanoglu, Tian Lin, Farha Deceus, Amber Heemskerk, Natalie C. Ebner, Brian S. Cahill

**Affiliations:** 1 Department of Psychology, University of Florida, 945 Center Dr, Gainesville, FL 32603 USA; 2 Department of Aging and Geriatric Research, Institute on Aging, University of Florida, Gainesville, USA; 3 Florida Institute for Cybersecurity, University of Florida, Gainesville, USA; 4 Evelyn F. McKnight Brain Institute, University of Florida, Gainesville, USA

**Keywords:** Real news, Fake news, Analytical reasoning, Cognitive reflection test, Source credibility, Perceived credibility, Dual-process theory, Naturalistic decision-making

## Abstract

**Aim:**

Previous research has focused on accuracy associated with real and fake news presented in the form of news headlines only, which does not capture the rich context news is frequently encountered in real life. Additionally, while previous studies on evaluation of real and fake news have mostly focused on characteristics of the evaluator (i.e., analytical reasoning), characteristics of the news stimuli (i.e., news source credibility) and the interplay between the two have been largely ignored. To address these research gaps, this project examined the role of analytical reasoning and news source credibility on evaluation of real and fake full-length news story articles. The project considered both accuracy and perceived credibility ratings as outcome variables, thus qualifying previous work focused solely on news detection accuracy.

**Method:**

We conducted two independent but parallel studies, with Study 2 as a direct replication of Study 1, employing the same design but in a larger sample (Study 1: *N* = 292 vs. Study 2: *N* = 357). In both studies, participants viewed 12 full-length news articles (6 real, 6 fake), followed by prompts to evaluate each article’s veracity and credibility. Participants were randomly assigned to view articles with a credible or non-credible source and completed the Cognitive Reflection Test as well as short demographic questions.

**Findings:**

Consistent across both studies, higher analytical reasoning was associated with greater fake news accuracy, while analytical reasoning was not associated with real news accuracy. In addition, in both studies, higher analytical reasoning was associated with lower perceived credibility for fake news, while analytical reasoning was not associated with perceived credibility for real news. Furthermore, lower analytical reasoning was associated with greater accuracy for real (but not fake) news from credible compared to non-credible sources, with this effect only detected in Study 2.

**Conclusions:**

The novel results generated in this research are discussed in light of classical vs. naturalistic accounts of decision-making as well as cognitive processes underlying news articles evaluation. The results extend previous findings that analytical reasoning contributes to fake news detection to full-length news articles. Furthermore, news-related cues such as the credibility of the news source systematically affected discrimination ability between real and fake news.

**Supplementary Information:**

The online version contains supplementary material available at 10.1186/s41235-021-00292-3.

## Introduction

Fake news refers to “fabricated information that mimics news media content in form but not in organizational process or intent” (Lazer et al., 2018, p. 1094). While fake news is certainly not a new occurrence—e.g., tabloid magazines have been around for nearly a century (Murray, 2013)—its prominence in and impact on our culture has been growing. This is also related to enhanced global connectedness and broader use of online media platforms in modern society which have drastically increased access to news but also increased distribution of misinformation via fake news. One study estimated that the average American encountered between one and three fake news articles during the month prior to the 2016 presidential election (Allcott & Gentzkow, 2017). Given the prevalence of fake news, the relevant question is, how good are people at detecting real and fake news? Recent polls indicate that a significant portion of Americans (47%) report having difficulty distinguishing between real and fake news (Associated Press, 2019). Analysis of Facebook activity of the top 20 fake and real news stories showed that user engagement was greater for fake compared to real news stories (Silverman et al., 2016). Further, in an analysis of 126,000 real and fake news stories tweeted by about 3 million Twitter users, fake compared to real news spread more than 4.5 million times faster and in a wider range (Vosoughi et al., 2018).

Thus, it is crucial to investigate the processes involved in the evaluation of real and fake news. Here, we will address the following understudied research questions: (1) Is current evidence regarding an impact of analytical reasoning on fake news detection robust to methodological change (i.e., by presenting full-length articles as opposed to headlines only)?; (2) Does systematically varying the credibility of the news source influence news article evaluation?; and (3) What can we learn from examining the perceived credibility of the news articles, beyond real and fake news detection accuracy?

## A cognitive account of fake and real news detection

According to Dual-Process Theory, individuals engage in two modes of information processing: a quick, intuitive mode (called *System 1*) and a slow, deliberate mode (called *System 2*; De Neys, 2012; Ferreira et al., 2006; Kahneman, 2011; Stanovich, 2009). System 1 is associated with low analytical reasoning and reliance on cognitive heuristics when making decisions (i.e., mental shortcuts based on prior knowledge and beliefs; Evans, 2007; Kahneman et al., 1982). System 2, in contrast, is associated with high analytical reasoning and involves careful and systematic consideration of information, and therefore, is less error prone than System 1.

In line with Dual-Process Theory, individuals who scored higher on a measure of analytical reasoning (i.e., Cognitive Reflection Test [CRT]; Frederick, 2005) were better at detecting fake news than individuals who scored low on analytical reasoning, regardless of whether the news content aligned with their political beliefs (Pennycook & Rand, 2019b; also see Bago et al., 2020; Pennycook & Rand, 2019a for evidence supporting the role of analytic reasoning over and above political ideology on fake news detection). Furthermore, engagement in analytic reasoning accounted for ~ 56% to 95% of the variance in accurate detection of fake news (Pennycook & Rand, 2020). Lastly, while delusion-prone individuals, dogmatic individuals, and religious fundamentalists were more likely to believe fake news, these relationships were partially or fully explained by lower levels of analytical reasoning (Bronstein et al., 2019). In sum, there appears to be consistent evidence that lower analytical reasoning is associated with poorer fake news detection.

## Current study

From previous research we know that the prevalence of fake news is significant and that individuals are poor at detecting fake news, due to low engagement of analytical reasoning. Previous research, however, focused on real and fake news detection accuracy using news headlines only, which does not capture the rich context news is frequently encountered in real life. Additionally, while previous studies considered characteristics of the evaluator (i.e., analytical reasoning), characteristics of the news stimuli (i.e., news source credibility) and the interplay between the two have been largely ignored. This paper went beyond previous work by employing full-length news articles (involving full news story along with a headline) to determine the role of: *(i) analytical reasoning* on evaluation of real and fake full-length news articles; *(ii) credibility of the news source* on evaluation of news articles; and *(iii) perceived credibility* of news articles, in addition to detection accuracy. Next, we will discuss the theoretical background leading to these central research aims.

### Impact of analytical reasoning on real and fake news evaluation for full-length articles

In a typical fake news paradigm, participants are presented with news headlines only that are either factually accurate (real news) or not (fake news). Following each headline, participants are asked to make a set of evaluations, including, but not limited to, veracity (i.e., real vs. fake), familiarity, and willingness to share. Given that in real life, people are not typically restricted to solely using the headline to evaluate a news article (i.e., people typically can go beyond browsing headlines and read the full article), we employed full-length news articles. Limited research has attempted to shift the research field by adopting more ecologically valid news evaluation methodology. Besides being more ecologically valid, full-length articles provide rather rich contextual information and a larger set of diagnostic cues to determine credibility of the news (e.g., coherence in story line, writing and grammatical style). These additional features of full-length news articles as opposed to news headlines only inform the news evaluation process. To our knowledge only Schaewitz et al. (2020) employed full articles and found that people with high compared to those with low need for cognition were less susceptible to misinformation via fake news. Their design, however, did not involve a systematic manipulation of news veracity as they only used fake news stories. Thus, systematic variation of news veracity within a relatively more naturalistic decision-making context that allows for full exploration of the entire article, as done in the present study, has potential to further understanding of the cognitive mechanisms underlying real and fake news evaluation.

According to the Naturalistic Decision Making framework (Klein, 2008, 2015), in fast-paced complex settings, decision makers mostly rely on past experiences to find the first workable option rather than trying to find the best possible solution, which requires analytical reasoning and is resource-intensive. People in real life come across real news more frequently than fake news (Allen et al., 2020; Guess et al., 2019). It is therefore possible that detection of real news relies on relatively more naturalistic decision-making processes which do not require analytical reasoning to the same extent as those involved in (less frequently encountered) fake news stories (Gigerenzer, 2007). Detection of fake news, in contrast, may rely more on deliberative processes that require high analytical reasoning and careful scrutinization of potential deceptive cues; which full-length news articles may be more diagnostic of than (brief) headlines. Based on these considerations, we predicted that higher analytical reasoning would be associated with increased fake news accuracy, while there would be no relationship between analytical reasoning ability and real news detection accuracy (Hypothesis 1).

### Effects of systematic variation of news source credibility on real and fake news evaluation

The Elaboration Likelihood Model put forth by Petty and Cacioppo (1986) is a dual-process model of persuasion. According to this model, information is processed via a central, systematic route when the decision maker is both motivated and has the necessary cognitive resources to do so. However, when the decision maker lacks either the necessary motivation or the cognitive resources, they will process information via a peripheral, heuristic route. Importantly, this model posits that heuristic cues such as the credibility of the source (in our case the news source of the article) will have a greater effect when the decision maker is processing the message via the peripheral route (Carpenter, 2015; Petty & Cacioppo, 1986; Ratneshwar & Chaiken, 1991). Thus, it is possible that news source credibility moderates real and fake news evaluation, especially when information is processed peripherally (i.e., involving lower analytical reasoning).

To our knowledge, there are no studies examining the impact of analytical reasoning on accuracy for both real and fake news under systematic variation of news source credibility. Given that individuals rely more on heuristics as cognitive resources decrease (Cacioppo et al., 1986; Petty & Cacioppo, 1986) and that low analytical reasoning is associated with reduced ability to detect fake news (Bronstein et al., 2019; Pennycook & Rand, 2019b), we hypothesized that lower analytical reasoning would be associated with increased accuracy for real and particularly fake news paired with a credible compared to a non-credible news source (*Hypothesis 2*).

### Beyond accuracy, the role of perceived credibility on real and fake news evaluation

Most fake news studies have focused on accuracy as the primary outcome measure, while neglecting perceived credibility of real and fake news as relevant evaluation metric. Pennycook and Rand (2019a) demonstrated that mainstream online news sources (e.g., cnn.com; npr.org) were perceived as more credible than online sources of partisan (e.g., breitbart.com; dailykos.com) or fake (e.g., thelastlineofdefense.org; now8news.com) news. This finding suggests that the source of a news item may be an important piece of information when evaluating the credibility of an article. Indeed, Luo et al. (2020) showed that perceived credibility of news headlines was greater when paired with more credible news sources (but see Schaewitz et al., 2020 for no effect of news source on perceived credibility of fake news).

Based on this evidence, we propose that perceived credibility may constitute a relevant, but currently understudied, construct involved in news evaluation. We hypothesized that higher analytical reasoning would be associated with less perceived credibility for fake news, while analytical reasoning ability would not affect perceived credibility of real news (*Hypothesis 3*). Furthermore, we predicted that lower analytical reasoning would be associated with greater perceived credibility for real and particularly fake news paired with a credible compared to a non-credible news source (*Hypothesis 4*).

## Method

To enhance scientific rigor and reproducibility (Open Science Collaboration, 2015), we adopted a two-study approach in this paper. In particular, we conducted two parallel but independent studies to systematically test in Study 1 and replicate with a large sample in Study 2 our research hypotheses.

### Participants

Study 1 recruited 360 undergraduates from the Department of Psychology’s SONA system. A total of 68 participants were removed from the final analysis for the following reasons: 3 had average reading times 3 *SD*s greater than the group average, 41 had incomplete news evaluation data, and 24 failed the attention checks (e.g., *Please answer 2 to this question*). The final analysis sample in Study 1 thus comprised 292 participants.

Study 2 used the same recruitment methods as Study 1; assuring through the SONA system that not the same participants were enrolled across the two studies. The initial sample consisted of 424 undergraduate students. A total of 67 participants were removed from the final analysis for the following reasons: 1 had average reading times 3 *SD*s greater than the group average, 42 had incomplete news evaluation data, and 24 failed the attention checks. The final analysis sample for Study 2 thus comprised 357 participants. Table 1 presents sample characteristics for participants in Study 1 and Study 2.

### Design

Both studies adopted a 2 (Veracity: real vs. fake; dichotomous; within-subjects) × 2 (Source: credible vs. non-credible; dichotomous; between-subjects) mixed design. Participants were randomly assigned to evaluate 6 real and 6 fake news articles either from credible (*N* = 138 in Study 1; *N* = 171 in Study 2) or non-credible (*N* = 154 in Study 1; *N* = 186 in Study 2) news sources (see below for more details).

### Materials

Study materials were identical in Study 1 and 2.

#### News articles

To select fake news articles, we used the “junk news” archive maintained by the reputable fact-checking website Snopes.com (Junk News Archives, n.d.). For real news articles, we used the “true news” archive maintained by Snopes (www.snopes.com/archive/) which involves news articles from reputable news organizations (e.g., Washington Post, NPR). From these archives, we selected 6 fake and 6 real news articles that varied by topic, including healthcare (e.g., doctors refusing care on religious grounds), religion (e.g., Mormonism and same-sex marriage, Pope Francis), education (e.g., California textbooks, guns on campuses), crime (e.g., prison escape, felony assault), and politics (e.g., the Black Lives Matter movement, gun confiscations). We conducted an independent pilot study with 98 college students from the Department of Psychology’s SONA system to assess the credibility of the selected 12 news articles (i.e., *How credible was this news article?*; rated on a scale from 1 = *Not at all credible* to 10 = *Completely credible*). Real news articles were rated as more credible (*M* = 5.90, *SD* = 1.09) than fake news articles (*M* = 4.00, *SD* = 1.39); *t*(97) = 13.40, *p* < 0.001).

We conducted an additional independent pilot study with 161 college students from the Department of Psychology’s SONA system to determine the final set of news sources for use in our study paradigm. Participants were asked to indicate the level of credibility (*How credible is this news source?*) on a scale from 1 = *Not at all credible* to 10 = *Completely credible* for 10 commonly known news organizations (i.e., 5 credible sources: NPR, CNN, Washington Post, New York Times, BBC; 5 non-credible sources: True Pundit, Conservative Daily News, World News Daily Report, Liberty Writers News, Red State). The three sources with the highest averages (i.e., NY Times [*M* = 7.00, *SD* = 2.30], Washington Post [*M* = 6.84, *SD* = 2.23], and NPR [*M* = 6.80, *SD* = 2.21]) were selected as “credible sources” and the three sources with the lowest averages (i.e., True Pundit [*M* = 4.30, *SD* = 1.70], Red State [*M* = 4.34, *SD* = 1.73], and Conservative Daily News [*M* = 4.55, *SD* = 1.83]) were selected as “non-credible sources” for use in the study. Additional file 1: Appendix A provides a full set of the news articles used in this project.

We created two experimental lists to control pairing of Veracity of the news article (real vs. fake; within-subjects) and Credibility of the news source (credible vs. non-credible; between-subjects). The two lists comprised the same 12 unique articles and were counterbalanced across participants. In List 1, the 6 real and the 6 fakes news articles were randomly paired with credible news sources (i.e., NY Times, Washington Post, NPR; credible condition). In List 2, the 6 real and the 6 fakes news articles were randomly paired with non-credible news sources (i.e., True Pundit, Red State, Conservative Daily News; non-credible condition). Presentation order within each list was pseudorandomized, with the constraint that the same type of news articles (real vs. fake) was not repeated more than two times in a row. For each list, (approximately) half of the participants received the reversed order to counter order effects.

#### Cognitive reflection test

The CRT (Frederick, 2005) is a three-item task designed to measure the degree to which analytical reasoning is used when solving problems. For example, one item asks: “*A bat and a ball cost $1.10 in total. The bat costs $1.00 more than the ball. How much does the ball cost?*” Participants high in analytical reasoning would overcome the impulse to give the intuitive answer 10 cents and would instead give the correct answer of 5 cents. Thus, a greater CRT score reflects higher analytical reasoning.

### Procedure

Study procedures for Study 1 and Study 2 were identical unless noted otherwise. Participants accessed the study link through the SONA system website and completed the study remotely through Qualtrics (https://www.qualtrics.com/). Prior to study enrollment, all participants consented electronically to participate.

During the *News Evaluation Task*, participants were presented with 12 news articles (6 real, 6 fake). Each article was presented on the screen for at least 60 s to ensure sufficient reading time, as determined in an internal pilot. Beyond the 60-s window, the task was self-paced.^1^ After reading each article, participants were prompted with the following questions (in this order): accuracy (*Is this news article real or fake?*; response option: *Real vs. Fake*), confidence (*How confident are you in your decision regarding the authenticity of this news article?*; response option: 1 (*Not at all confident*) to 10 (*Completely confident*)), perceived credibility (*How credible do you find this news article?*; response option: 1 (*Not at all credible*) to 10 (*Completely credible*)), media sharing (*Would you share this news article on social media?*; response option: *Yes vs. No*), and familiarity (*Have you seen this article before?*; response option: *Yes vs. No*). Participants were not informed about the number of articles presented to them to avoid response biases (e.g., 50/50 real vs. fake response rate).

After evaluating the news articles, participants completed the CRT and a short demographic questionnaire.^2^ Study duration was about 1 h in each of the two studies.

### Data analysis

We used multilevel random intercept models (Gelman & Hill, 2007; Hox, 2010) to accommodate for the nested data structure. Specifically, we conducted cross-random effects analyses with cross-classification of news articles and participants, and a nesting structure for repeated observations within participants. This approach allows evaluations made by the same participant to be correlated across different news articles, as well as accounts for dependencies of evaluations of the same news article made by different participants.

Our analyses included two separate models, one for accuracy^3^ and one for perceived credibility. Complete datasets and analysis code can be found at https://osf.io/yrabp/. For the binary outcome variable *accuracy* (0 = wrong, 1 = correct), we used mixed effects logistic regression; for the ordinal/continuous outcome variables *perceived credibility* we employed multilevel regression. Each model considered the fixed effect of veracity of the news article (0 = real, 1 = fake), credibility of the source (0 = credible, 1 = non-credible), and the CRT score of each participant (continuous variable) as predictors. We further estimated the interactions between these independent variables in each model. We also entered the random intercepts of evaluations for news articles and participants to estimate the variability of mean evaluations across news articles and participants, respectively. Reading time (beyond the fixed 60-s window), familiarity, gender, and presentation order were entered as covariates.

We applied maximum likelihood estimation for all model parameters and used the Wald tests to determine significance of the effects. For significant interactions, we compared (using *z* tests for pairwise comparisons) and plotted predicted marginal means (using a mean of 0 and ± 1 *SD* for interactions involving the continuous CRT variable) from the estimated model parameters to facilitate understanding of significant interactions. All analyses were performed in Stata 16.1 (StataCorp, 2019).

## Results

### Accuracy

**Fig. 1:**
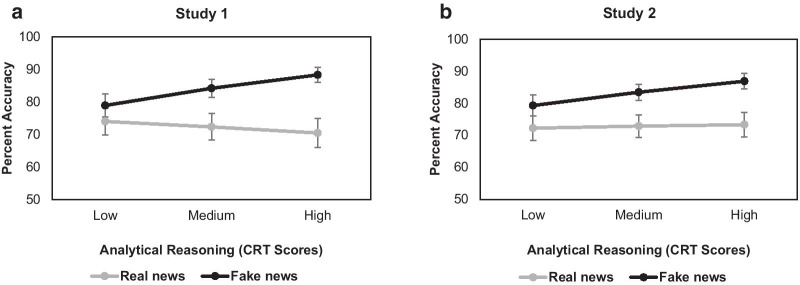
Percent accuracy for real (gray line) and fake (black line) news articles across levels of analytical reasoning (continuous; indexed by Cognitive Reflection Test (CRT) scores) in Study 1 (Panel A) and Study 2 (Panel B). Error bars denote standard errors. The medium analytical reasoning level indicates the mean CRT score in the current sample while the low and high levels indicate 1 *SD* below and above the mean CRT score, respectively. The y-axis start point reflects the 50% chance level. Consistent across both studies, real news accuracy did not change across levels of analytical reasoning, while accuracy for fake news increased with higher analytical reasoning

Consistent across both studies, the Veracity × CRT interaction on accuracy was significant [Study 1: *χ*^2^_(1)_ = 23.84, *p* < 0.001; Study 2: *χ*^2^_(1)_ = 10.78, *p* = 0.001]. As shown in Fig. 1, real news accuracy did not change across levels of analytical reasoning (indexed by CRT scores) [Study 1/Panel A: *z* = 1.37, *p* = 0.339; Study 2/Panel B: *z* = 0.5, *p* = 0.619]. Accuracy for fake news, however, increased with higher analytical reasoning [Study 1/Panel A: *z* = 4.53, *p* < 0.001; Study 2/Panel B: *z* = 4.13, *p* < 0.001], thus supporting *Hypothesis 1*. Furthermore, also consistent across both studies and depicted in Fig. 1, higher analytical reasoning was associated with better detection of fake than real news [Study 1/Panel 1A: *z* = 3.79, *p* < 0.001; Study 2/Panel 1B: *z* = 3.43, *p* = 0.001].

**Fig. 2:**
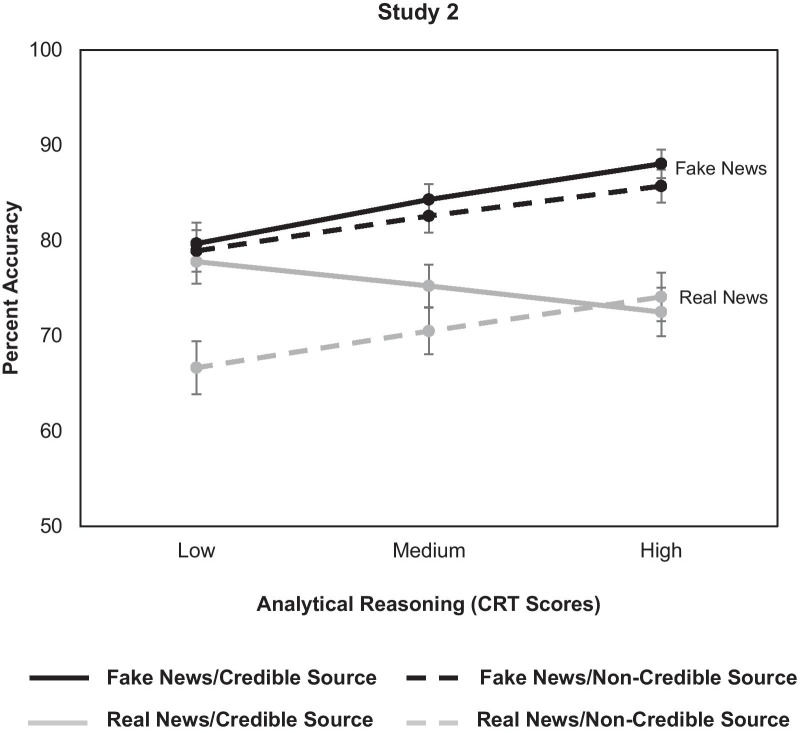
Veracity × CRT × Source interaction in Study 2; this 3-way interaction was not significant in Study 1. Percent accuracy for real (gray lines) and fake (black lines) news articles from credible (solid lines) and non-credible (dashed lines) news sources across levels of analytical reasoning (continuous; indexed by Cognitive Reflection Test (CRT) scores) in Study 2. Error bars denote standard errors. The medium analytical reasoning level indicates the mean CRT score in the current sample while the low and high levels indicate 1 SD below and above the mean CRT score, respectively. The y-axis start point reflects the 50% chance level. Lower analytical reasoning was associated with greater accuracy for real news paired with credible compared to non-credible sources, while news source did not influence accuracy for fake news across levels of analytical reasoning

The three-way interaction between Veracity × CRT × Source was not significant in Study 1 (*χ*^2^_(1)_ = 0.42, *p* = 0.517), but was significant in Study 2 (*χ*^2^_(1)_ = 6.2, *p* = 0.013). In particular, as shown in Fig. 2 (for Study 2), lower analytical reasoning was associated with greater accuracy for real news from credible compared to non-credible sources (*z* = 3.42, *p* = 0.001). Furthermore as depicted in Fig. 2, news source credibility did not influence accuracy for fake news across levels of analytical reasoning (*z*s < 1, *p*s > 0.272); and higher analytical reasoning was associated with greater accuracy for fake news irrespective of news source credibility (all *z*s > 2.55, *p*s < 0.02). These findings partially supported *Hypothesis 2*.^4^

### Perceived credibility

**Fig. 3:**
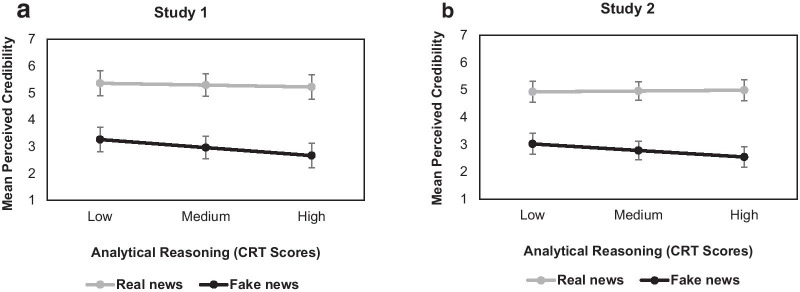
Mean perceived credibility rating (1 = *Not at all credible* to 10 = *Completely credible*) for real (gray line) and fake (black line) news articles across levels of analytical reasoning (continuous; indexed by Cognitive Reflection Test (CRT) scores) in Study 1 (Panel A) and Study 2 (Panel B). Error bars denote standard errors. The medium analytical reasoning level indicates the mean CRT score in the current sample while the low and high levels indicate 1 *SD* below and above the mean CRT score, respectively. Note that the y-axis ranges from 1 to 7 to reflect the actual range of responses given by participants. Consistent across both studies, perceived credibility for real news was not influenced by levels of analytical reasoning, while higher analytical reasoning was associated with less perceived credibility for fake news

Consistent across both studies, the Veracity × CRT interaction was significant [Study 1: *χ*^2^_(1)_ = 14.28, *p* < 0.001; Study 2: *χ*^2^_(1)_ = 24.57, *p* < 0.001]. As depicted in Fig. 3, perceived credibility for real news was overall higher than perceived credibility for fake news and was not influenced by levels of analytical reasoning [Study 1/Panel A: *z* = 0.97, *p* = 0.66; Study 2/Panel B: *z* = 0.52,* p* = 0.6]. In contrast, higher analytical reasoning was associated with less perceived credibility for fake news [Study 1/Panel A: *z* = 4.22, *p* < 0.001; Study 2/Panel B: *z* = 3.55, *p* < 0.001], in line with *Hypothesis 3*.

The three-way interaction between Veracity × CRT × Source was not significant in either of the studies [Study 1: *χ*^2^_(1)_ = 1.49, *p* = 0.222; Study 2: *χ*^2^_(1)_ = 0.67, *p* = 0.413]. Thus, our data did not support *Hypothesis 4*.

## Discussion

The present two-study project, with a built-in replication, is the first to examine evaluation of both real and fake news under consideration of cognitive factors (i.e., analytical reasoning), characteristics of the news stimuli (i.e., source credibility) as well as the interplay between the two using a novel, relatively more ecologically valid full-length article paradigm. In addition, our approach went beyond investigation of real and fake news evaluation accuracy in also determining effects on the perceived credibility of the articles. Consistent across both studies, higher analytical reasoning was associated with greater accuracy and reduced perceived credibility for fake news, while analytical reasoning ability did not moderate accuracy and perceived credibility of real news. Furthermore, in Study 2 (but not in Study 1) news source credibility influenced the relationship between analytical reasoning ability and news detection accuracy for real (but not fake) news. These novel findings have potential to advance theory and empirical understanding of cognitive processes underlying news evaluations, as discussed next.

### Higher analytical reasoning improves fake news detection in full-length articles

Consistently across both studies and in line with our predictions, higher analytical reasoning was associated with more accurate detection of fake news articles. Thus, extending previous evidence from headlines-only studies (Bronstein et al., 2019; Pennycook & Rand, 2019a, 2020; Pennycook et al., 2015), by using full-length news articles the present study provides support for a role of analytical reasoning on fake news detection. In line with our prediction, real news accuracy, in contrast, was not influenced by analytical reasoning ability. As real news is more common in everyday life than fake news, detection of real news may not be as resource-demanding than detection of fake news, possibly underlying the moderating effect of analytical reasoning on fake but not real news detection. The Naturalistic Decision Making framework (Klein, 2008, 2015) highlights the role of relatively automatic (intuitive) and experience-based successful decision making in naturalistic real-world settings. This framework may be particularly fruitful in future research on determining the mechanisms underlying news evaluation. As touched on earlier, we believe that our full-length article approach is more representative of how news articles are typically encountered in real life (e.g., with rich contextual information), thus allowing to better capture complex cognitive processes involved in naturalistic news evaluation. To further improve ecological validity, future research could leverage real or simulated social media platforms (e.g., Twitter, Facebook), where people directly interact with the news (see Lin et al., 2019, for a similar approach in email phishing detection). This approach would also be in line with research demonstrating the importance of using ecological valid task formats to improve performance (Evans, 2011; Mercier & Sperber, 2011). The present study constitutes a first important step in this direction.

Further, consistent across both studies, higher analytical reasoning was associated with better detection of fake than real news. One could argue that better detection of fake compared to real news with higher analytical reasoning may simply reflect a response bias (i.e., tendency to overclaim news as fake, which could be an artifact of task instructions). However, results from an additional analysis we conducted that controlled for sensitivity and response bias did not support this interpretation. Instead, this finding may reflect an enhanced ability to detect deceptive cues inherent in fake news stories among individuals who engage in higher levels of analytical reasoning. That is, diagnostic cues and details in the full-length fake news articles used in this study such as pertaining to formatting, grammar issues, general writing style (and that may not be present in real news articles) may have facilitated fake news detection among individuals who engage in deeper processing (i.e., higher analytical reasoning). These explanations are rather speculative and warrant research that uses natural language processing machine learning approaches (Gilda, 2017; Oshikawa et al., 2018), for example, to determine deception-related diagnostic cues in fake (relative to real) news and to further clarify the interplay between these cues and analytical reasoning ability in news detection.

### Lower analytical reasoning enhances detection of real news paired with credible sources

We found that lower analytical reasoning was associated with better detection of real news paired with credible sources, while news source credibility did not influence accuracy for fake news across levels of analytical reasoning. To date only a small number of studies have examined the impact of source credibility on news detection accuracy. Luo et al. (2020) showed that reliability of the source (indexed by a high number of Facebook likes) increased the detection of real news but decreased the detection of fake news. In contrast, Schaewitz et al. (2020) found no effect of source credibility (i.e., fictitious news sources that were rated on credibility) on fake news accuracy. Furthermore, Pennycook and Rand (2020) reported a negative association between analytical reasoning and susceptibility to fake news, regardless of whether a news source was present or absent, suggesting no moderating effect of source credibility on the relationship between analytical reasoning and fake news detection (also see Dias et al., 2020 for similar results).

Our study contributes to this literature and is the first to suggest that news source credibility may influence news detection as a function of analytical reasoning in full-length real (but not fake) news articles. However, this finding only emerged in Study 2 but not in Study 1 and thus needs to be interpreted with caution. It is possible that lower analytical reasoning reflects greater reliance on source heuristics. In fact, our results are consistent with predictions from the Elaboration Likelihood Model (Petty & Cacioppo, 1986), which proposes that peripheral cues such as the credibility of the source of a message, more likely influence individuals low in cognitive resources as they engage in less elaborative or systematic processing; a possible explanation that can be systematically explored in future work. Also, as the three-way Veracity × CRT × Source interaction was only significant in Study 2, which comprised a larger sample size, but not in Study 1, a future replication of this effect in a sample of at least the size as in Study 2 is warranted to corroborate the finding. Additionally, because of study duration related constraints and our preference for keeping our news article material uniform across participants (i.e., each participant viewed the same real and fake news articles), the credibility of the source the news articles were paired with was manipulated between participants in this project. This design feature may have reduced statistical power to detect significant effects related to source credibility (i.e., one would expect greater sensitivity of a factor that is manipulated within-subjects (in this case, veracity) than one that is manipulated between-subjects (in this case, news source credibility)). Future studies could employ a within-subjects design to investigate this possibility.

### Beyond accuracy, perceived credibility as an additional route to study cognitive mechanisms underlying news evaluation

Overall, perceived credibility for real news was higher than perceived credibility for fake news in both studies. Furthermore, and again consistent across both studies, higher analytical reasoning was associated with lower perceived credibility for fake news, while perceived credibility for real news did not vary by level of analytical reasoning.

Somewhat in contrast to our findings pertaining to accuracy, news source did not moderate the effect of analytical reasoning on perceived credibility of real vs. fake news. Specifically, participants who relied more on analytical reasoning were better at detecting fake news and rated fake news as less credible. Importantly, the credibility of the news source did not affect accuracy or perceived credibility of fake news in individuals high on analytical reasoning. This finding may suggest that individuals high on analytical reasoning utilize diagnostic cues and contextual features provided within the fake news article itself (e.g., sentiment, formatting style, grammar issues, general writing style).

If this interpretation is true, then this highlights two important implications for future research. First, future research may benefit from using full-length news articles because headlines only contain a finite amount of diagnostic cues and may strip away important information to discern between real and fake news. Given that our current results (using full-length articles) align with past research that used only headlines, future research needs to directly compare full-length articles with headlines only and by systematically manipulating news source among individuals with varying levels of analytical reasoning to better assess these claims. Second, the aforementioned pattern emerged clearer by collecting novel outcome measures (i.e., perceived credibility of the news), thus, supporting the need for future research to explore other (sensitive) outcome measures (e.g., news content related questions) that may help gain a more complete understanding of the phenomenological process individuals engage in when detecting fake news.

Additionally, the possibility that participants may have directed their attention primarily towards the news stories and its central content (e.g., sentiment, language style) rather than peripheral cues (e.g., the news source) can be further investigated using eye-tracking. This technique will allow determination of eye gaze patterns as well as physiological reactions associated with arousal levels (e.g., pupil dilation) when interacting with news stories. These innovative methodological approaches would not only help identifying candidate cognitive mechanisms but could also inform targeted interventions (e.g., eye-tracking guided reading intervention to train people to process information relevant to detection of deceptive cues). This rich data will also lend itself particularly well to computational modeling approaches to describe decision-making processes underlying deception detection (see Hakim et al., 2020 for a computational modeling approach to phishing email detection).

### Future research directions

Our study, like the majority of previous work, focused on a rather homogeneous (e.g., in terms of race/ethnicity and age) sample. Based on growing evidence that sensitivity for detection of deceptive cues decreases with chronological age (Ebner et al., 2020; Grilli et al., in press; Zebrowitz et al., 2018) as well as varies by gender and marital status (Alves & Wilson, 2008), education (Wood et al., 2018), and income (James et al., 2014), we propose examining fake news detection using more diverse samples to move this research forward (Pehlivanoglu et al., 2020). For example, older compared to younger individuals were more likely to share fake news (Grinberg et al., 2019; Guess et al., 2019). A recent narrative review by Brashier and Schacter (2020) argues that susceptibility to fake news with age may not only depend on cognitive decline, but may also be related to age-related changes in socioemotional functioning (e.g., increase in positive emotion and interpersonal trust) as well as in expertise with online news media platforms. Thus, examining the role of expertise with online news media outlets (e.g., indexed by digital literacy; Sengpiel & Dittberner, 2008, and news media literacy; Maksl et al., 2015) on the relationship between analytical reasoning and real vs. fake news evaluation in a sample of adults varying in age (college students vs. middle-aged adults vs. older adults) is a fruitful future research direction. These future age-comparative studies would also be helpful to identify mechanisms that may render certain groups particularly vulnerable to fake news and would open tremendous potential for interventional approaches, including particular at-risk populations (Ebner, et al., in press).

Future studies should also set out to determine the specific dynamics of the impact of analytical reasoning on real and fake news evaluation. For example, it is possible that news related variables such as news topics/content (e.g., politics vs. pop culture) differentially call on analytical reasoning ability when evaluating real and fake news articles. In addition, it is possible that individuals can flexibly allocate their resources and switch between processing modes (e.g., effortful vs. non-effortful thinking; shallow vs. deep processing) for improved news evaluation. Utilizing neuroimaging techniques (e.g., fMRI) could help outline the neurocognitive mechanisms underlying news evaluation. Event-related potentials could help determine temporal dynamics of engagement in different levels of reasoning during news evaluation (e.g., whether engagement in analytic reasoning changes during early vs. late stages of processing; whether one reasoning mode is replaced by the other over time; whether news-related variables such as source credibility moderates these processes).

## Conclusions

This study is the first to demonstrate a positive association between analytical reasoning and fake news detection accuracy using full-length news articles, as a relatively more ecologically valid approach in research on news evaluation. The study is also first in supporting a moderating role of news source credibility in the endeavor to delineate cognitive mechanisms underlying news evaluation; and it advances knowledge pertaining to perceived credibility of news as an alternative outcome variable to accuracy. Across two independent studies, findings from this research underline the importance of both individual differences and news-related characteristics when evaluating news. Our research has potential for theoretical advancement regarding relative contributions of rational vs. more naturalistic decision making in the applied context of fake news detection. Employing full-length news articles, novel findings reported here spur future research hypotheses regarding the (neuro)cognitive mechanisms involved in detection of deceptive cues in news evaluation as well as possible intervention designs to tackle the major and daily growing threat of misinformation from fake news, at both individual and societal levels.

**Table 1:** Sample characteristics in Study 1 and Study 2

	Study 1 (*N* = 292)	Study 2 (*N* = 357)
**Mean Age** (in years) ± *SD*	18.98 ± 1.81	20.45 ± 2.99
**Gender**		
Male	37%	26%
Female	62%	72%
Other	1%	2%
**Race/Ethnicity**		
White (non-Hispanic/Latino)	53%	59%
Asian	16%	10%
Hispanic/Latino	15%	18%
Black/African-American	6%	6%
Other	1%	3%
Multiple	9%	4%
**Political Affiliation**		
Republican	N/A	29%
Democrat	N/A	46%
Other	N/A	25%

Note. SD *= *standard deviation

## Supplementary Information

**Additional file1. Appendix A:** A full set of the news articles used in the current project.

**Additional file 2. Appendix B:** Confidence ratings and sharing responses for the news articles.

## Data Availability

The stimulus set, complete datasets used in the analyses, and analysis code are available in the OSF repository, https://osf.io/yrabp/. None of the experiments were preregistered.

## Abbreviations

CRT: Cognitive reflection test

## Footnotes

1 Reading time data (in seconds; averaged across real and fake news articles) showed that participants took more than 60 s on average [Mean = 106.15 (Study 1), 101.53 (Study 2); Median = 89.25 (Study 1), 87.26 (Study 2); *SD* = 48.69 (Study 2), 48.13 (Study 2); Range = 62.15–363.15 (Study 1), 61.93–404.48 (Study 2)], suggesting that the news articles were processed adequately.

2 Both Study 1 and Study 2 also included the Gullibility Scale (Teunisse et al., 2020) and the short form of the Need for Cognition Scale (Cacioppo et al., 1984). Study 2 additionally included measures for religiosity (Batson & Schoenrade, 1991), spirituality (Büssing et al., 2007), conservatism (Everett, 2013), and media consumption habits (adopted from Maksl et al., 2015). These additional constructs were outside the scope of this report and were therefore not included in the statistical analysis for parsimony.

3 To support findings for accuracy, we conducted parallel analyses on confidence ratings and report the results in Additional file 2: Appendix B. News sharing was not analysed as an outcome measure due to floor effects in “yes” responses (see Table B1 in Additional file 2: Appendix B).

4 To ensure that our results regarding accuracy were not confounded by response bias, based on signal detection theory (Macmillan & Creelman, 2004), we computed sensitivity (d’ = z(Hit rate)—z(False alarm rate)) and response bias (c = − 0.5[z(Hit rate) + z(False alarm rate)]) for each participant, in both Study 1 and Study 2. Then, we added the scores for sensitivity and response bias as covariates and re-ran the analyses pertaining to accuracy. This re-analysis resulted in the same findings as our original analysis. In particular, the Veracity × CRT interaction was significant in both studies [Study 1: *χ*^2^_(1)_ = 21.92, *p* < 0.001; Study 2: *χ*^2^_(1)_) = 10.01* p* = 0.002]. The three-way interaction between Veracity × CRT × Source was not significant in Study 1 (*χ*^2^_(1)_ = 0.03, *p* = 0.857), but was significant in Study 2 (*χ*^2^_(1)_ = 5.8, *p* = 0.016).
