# Supplementary material for: The role of analytical reasoning and source credibility on the evaluation of real and fake full-length news articles
Source: Cogn Res Princ Implic. 2021 Mar 31;6:24. doi: 10.1186/s41235-021-00292-3 (PMC8012428; doi:10.1186/s41235-021-00292-3)
Supplement: Supplementary file 1 — Additional file1. Appendix A: A full set of the news articles used in the current project. [file 41235_2021_292_MOESM1_ESM.pdf]

Appendix A

Real News Articles with Credible News Source

The New York Times

A passerby heard a newborn crying in a storm drain. Four hours later, she was rescued.

By Gianluca Mezzofiore
Updated 11:32 AM ET, Tue February 12, 2019

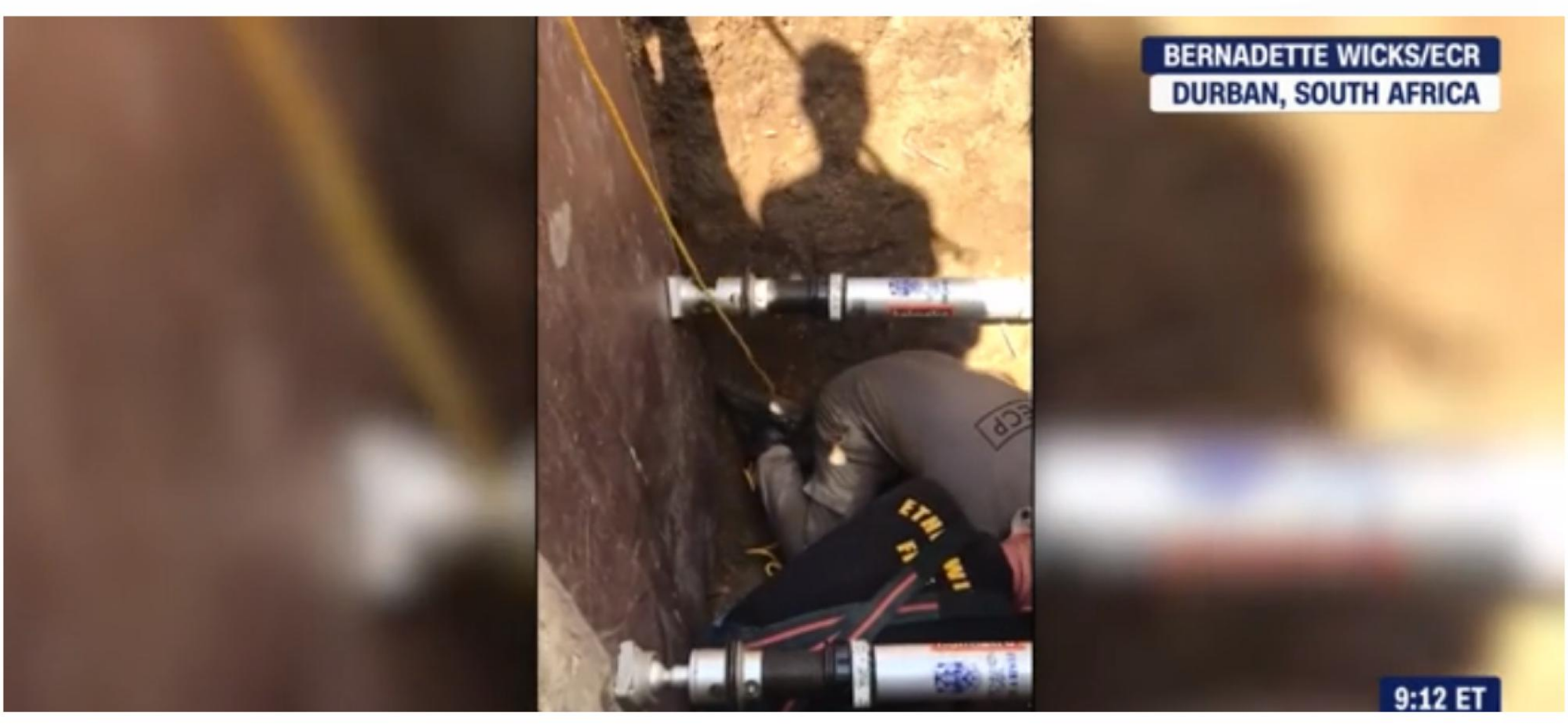

A baby girl was rescued from a storm drain in Durban, South Africa, after residents heard her crying and alerted emergency services.

A bystander had spotted the newborn after climbing down into the drain on Monday, Garrith Jamieson, head of Durban Rescue Care, said in a statement obtained by CNN.

Rescue services used a chisel and hammer to break into the drain, Jamieson said. The whole operation lasted nearly four hours.

After being located and extricated, the baby was flown to Inkosi Albert Luthuli Hospital for urgent care.

Dramatic video from the scene showed the moment the baby was lifted to safety.

Bernadette Wicks (Wolhuter)
@bern_wicks

The incredible moment a newborn baby girl is rescued from more than seven metres down a storm water pipe. The amazing men & women from Durban's emergency services have been working for nearly three hours to save the infant. #NewlandsEastBabyRescue @ECR_Newswatch

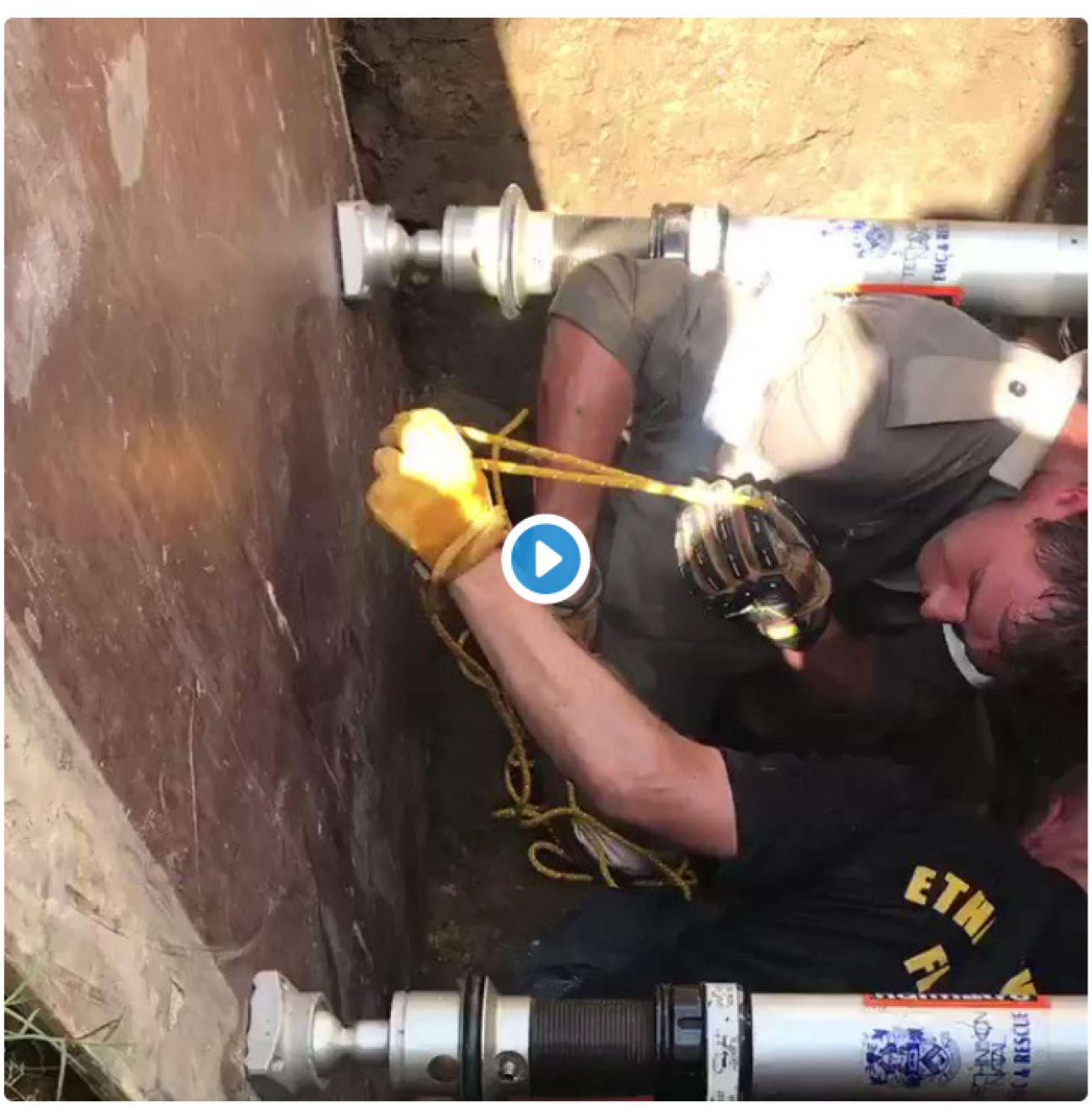

1,559 3:29 AM - Feb 11, 2019

895 people are talking about this

The hospital's trauma specialist, Dr Timothy Hardcastle, said the baby was suffering from hypothermia and had to be warmed up, but was in satisfactory condition with only minor injuries, according to a statement from the KwaZulu-Natal (KZN) province department of health.

The baby, who was found with her umbilical cord intact, was believed to be between one and three days old.

She was named Sibanisethu (Our Ray of Light) and Gabriella by residents, the statement said.

Dr Sibongiseni Dhlomo, MP
about 5 months ago

MEDIA RELEASE:

MIRACULOUS BABY RESCUE: MEC DHLOMO SAYS BABY ABANDONMENT IS A SIGN THAT PROGRAMMES PROMOTING CONTRACEPTIVES, CONDOMS NEEDS TO BE STRENGTHENED

11 February 2019... See More

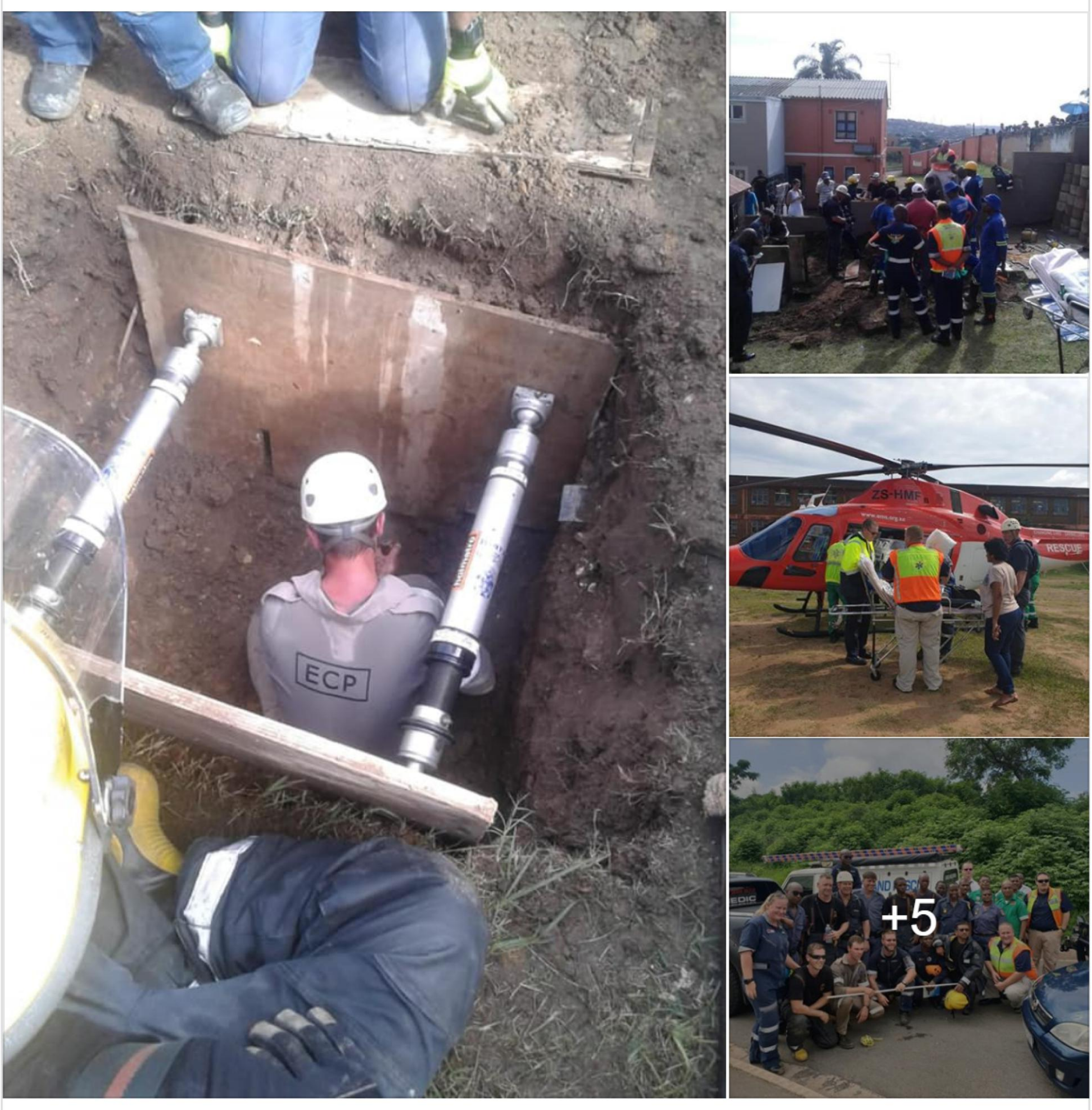

27 7 13

"It's really a miracle that this baby was saved. The doctors have given this baby a clean bill of health, and she will be transferred to another hospital," said regional health official Dr. Sibongiseni Dhlomo.

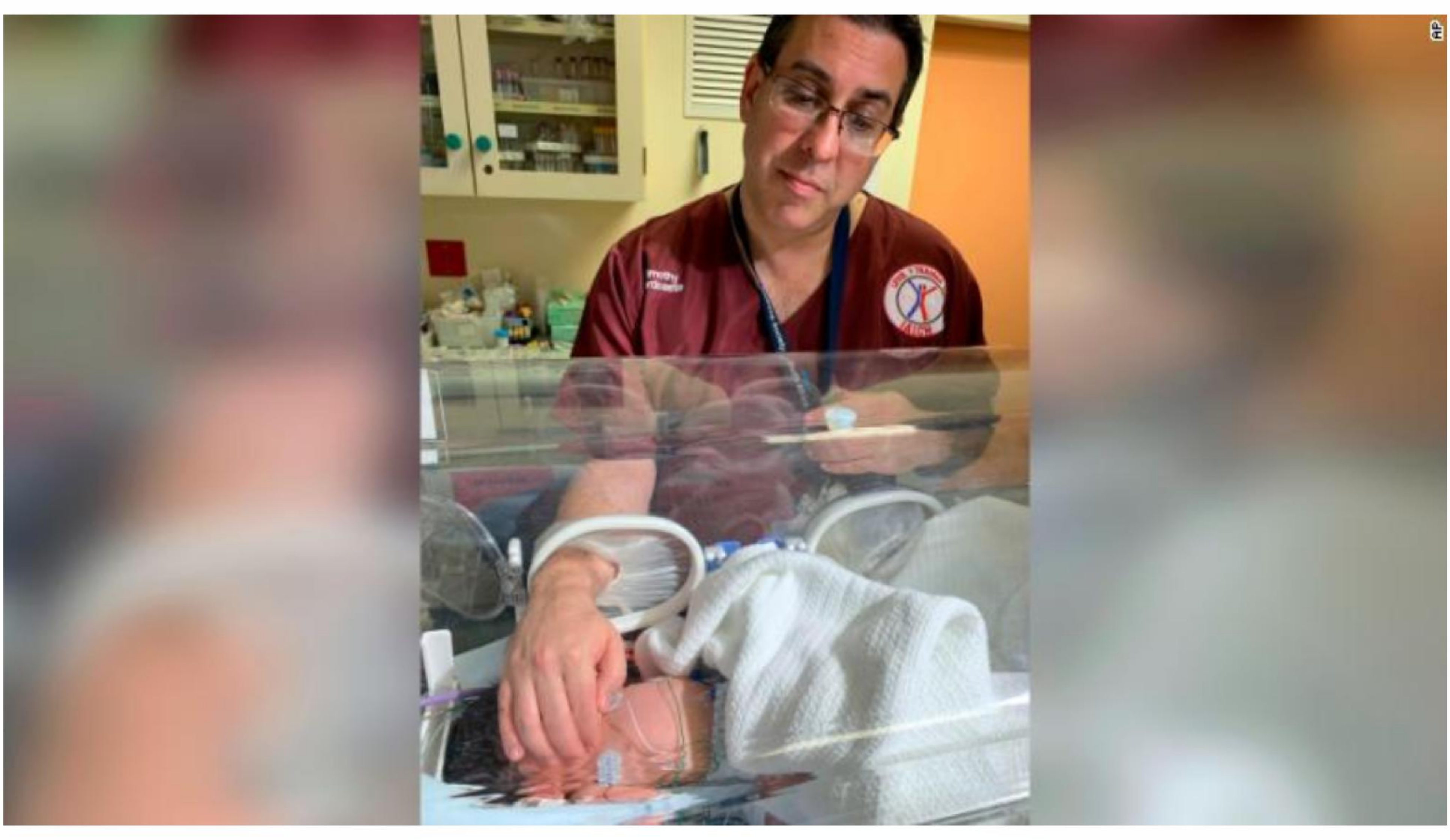

Doctor Timothy Hardcastle treats a baby in the hospital after she was rescued from a storm water pipe in Durban, South Africa.

KZN police spokesperson Col. Thembeka Mbele said a case of attempted murder was opened at Newlands East police station, according to Times Live. Police are investigating how the newborn ended up in the storm drain.

Texas Senate passes bill allowing school marshals to carry, not lock up, their guns

Eliminating the lockbox requirement for school marshals was one of many suggestions Gov. Greg Abbott outlined for the Legislature in a 43-page plan released weeks after last year's deadly shooting at Santa Fe High School.

BY ALEX SAMUELS APRIL 3, 2019

REPUBLISH

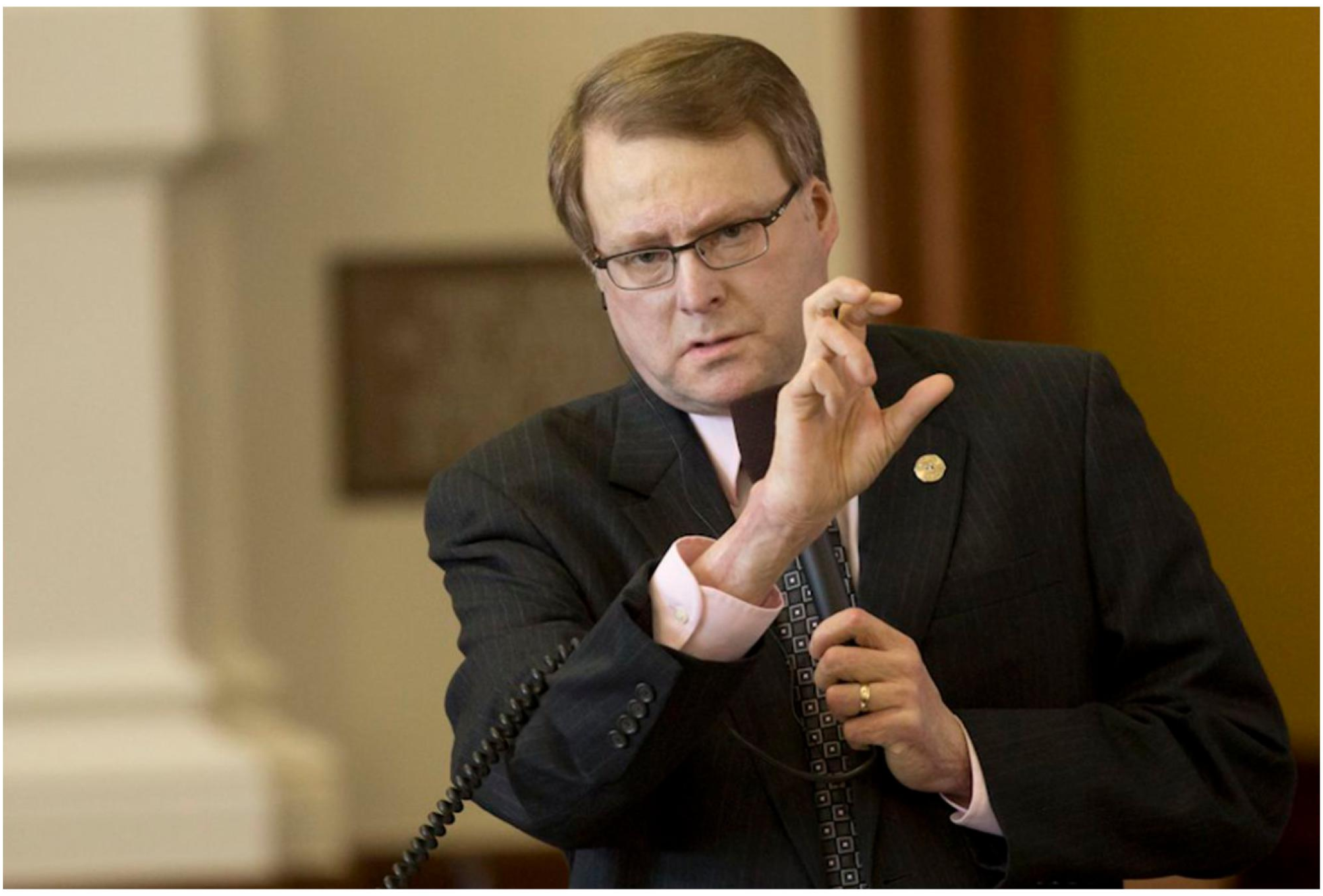

Senate Bill 406, by Republican state Sen. Brian Birdwell of Granbury, would eliminate the mandate that trained school marshals keep their firearms under lock and key. Marjorie Kamys Cotera for The Texas Tribune

After a brief debate, the Texas Senate approved a bill Wednesday that would allow local school boards to let their marshals carry their concealed guns on campuses.

The legislation — Senate Bill 406 by Republican state Sen. Brian Birdwell of Granbury — would eliminate the mandate that trained school marshals, whose identities are kept secret from all but a few local officials, keep their firearms under lock and key.

More specifically, it would give the board of trustees or the governing body of public schools, open-enrollment charter schools, private schools and junior colleges the discretion to decide whether their marshals carry their weapons on their person or in a locked and secured safe.

The measure passed 28-3, with Democratic state Sens. José Menéndez of San Antonio, José Rodríguez of El Paso and Kirk Watson of Austin voting against it. The bill can now be sent to the Texas House for debate.

“The code we’re currently dealing with specifically requires that those individuals who are designated as school marshals store their handguns in a locked box at all times,” Birdwell told other senators. “While a marshal would be acting in accordance with this state law, the practicality of this security is nullified if a school marshal cannot protect their students at the critical time and the critical place.”

To be clear, SB 406 does not compel school districts to implement a marshal program. Nor does it compel local districts to force marshals to openly carry their weapons.

“School districts retain the discretion to decide whether to implement the school marshal program fully, not at all or to some degree,” Birdwell said.

Eliminating the lockbox requirement for school marshals was one of many suggestions Gov. Greg Abbott outlined for the Legislature in a 43-page plan released weeks after a deadly shooting at Santa Fe High School in May that left 10 dead and 13 others wounded.

Gun control advocates, meanwhile, criticized the passage of the bill. In a statement, the Texas chapter of Moms Demand Action for Gun Sense in America, said SB 406 “would make school a much more dangerous place for our children.”

“It’s baffling that lawmakers are trying to strip gun storage standards in our schools,” wrote Hilary Whitfield, volunteer leader with the Texas chapter of Moms Demand Action for Gun Sense in America. “That is a recipe for disaster.”

During a brief floor debate, some Democrats echoed these worries.

The New York Times

'Cocaine King Of Milan' On The Run After Uruguay Jailbreak

June 25, 2019 · 6:41 PM ET

VANESSA ROMO

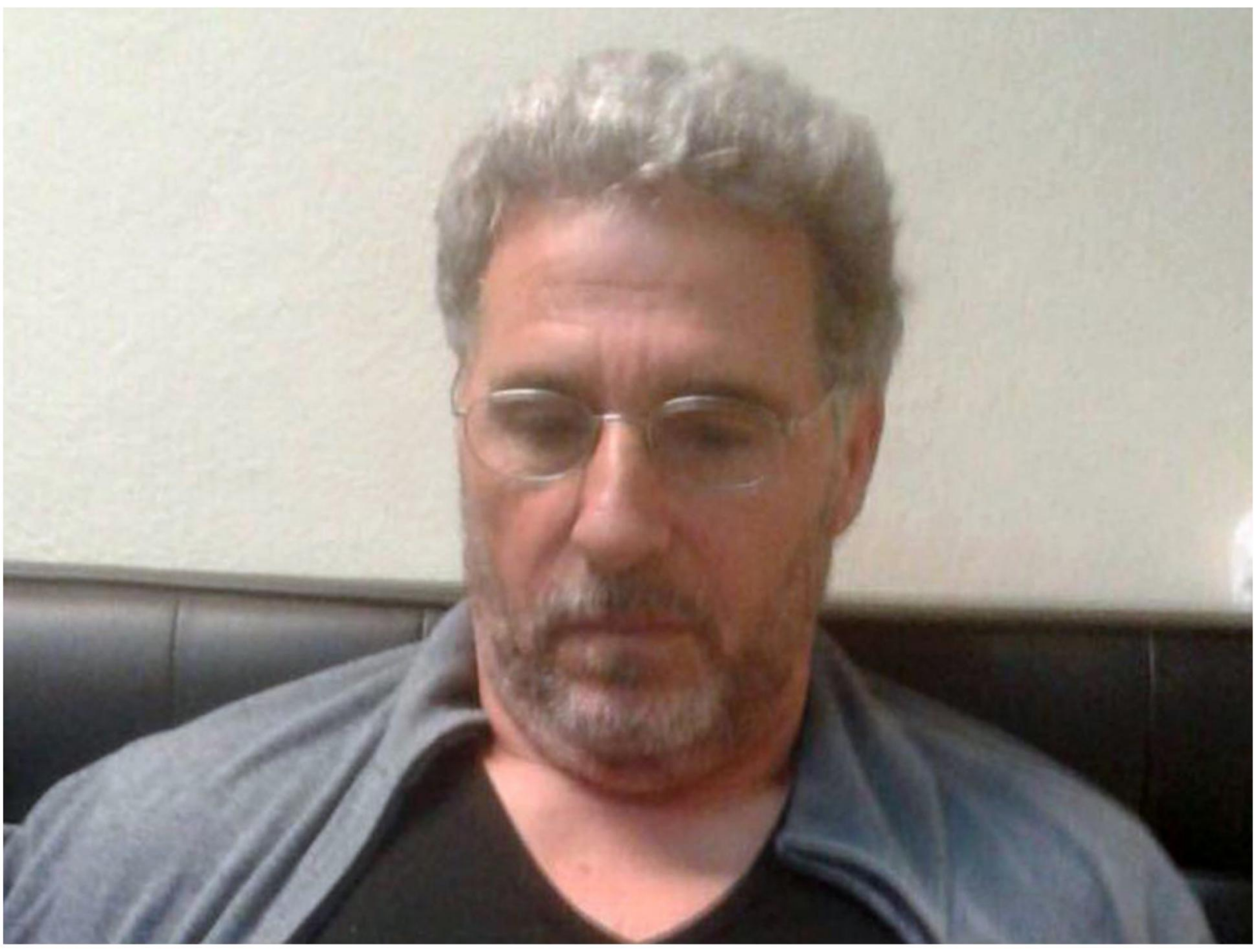

Rocco Morabito, pictured after his arrest in 2017, escaped from the Uruguayan prison where he was awaiting extradition to Italy.

Italian Police via AP

Uruguayan officials have launched a manhunt for an Italian organized crime boss known as the Cocaine King of Milan who escaped on Sunday from a detention center where he was awaiting extradition to Italy, the Uruguayan Ministry of Interior said in a statement.

Rocco Morabito and three other inmates made a brazen escape from the prison in Montevideo, climbing through a hole in the roof of the building. Reports from ministry officials indicated that the men eventually broke into a neighboring property, robbed the owner, then fled.

But *El Observador* reports that one of the four men, a Brazilian also waiting to be extradited to his home country, actually avoided the hassle of the daring jailbreak by simply walking through a side door of the building "without anyone stopping him."

Morabito, who is believed to be the son of another famed mobster with the same name, was one of the 10 most wanted criminals in the world in 2017 as the leader of the Calabrian 'Ndrangheta — one of Italy's most powerful organized crime groups.

A 2013 Europol report found the 'Ndrangheta "now recognised as a major threat not only in Italy but also in many other countries where it operates, including Germany, Spain, the Netherlands, France, Belgium, Switzerland, Canada, US, Colombia and Australia."

The drug kingpin was sentenced in absentia to 30 years in prison in Italy. Authorities have been chasing him since he was caught trying to import nearly a ton of cocaine into the country from Brazil in 1994.

Morabito had been living on the run for more than two decades under a false identity but was eventually captured after trying to enroll his daughter in a school using his real name.

El Observador reported the director of prisons has resigned following Sunday's escape.

According to the newspaper, officials were warned a year ago about an escape plan hatched by Morabito bearing an eerie resemblance to the weekend's events. The only major difference between the 2018 plan and what happened around midnight Sunday is that in the earlier version Morabito was set to escape from the sixth floor of the penitentiary onto the roof of a supermarket — not a neighboring apartment building.

Italy's minister of the interior, Matteo Salvini, was furious over the breakout.

"I make two commitments. First: to shed light on the methods of evasion, asking for immediate explanations from the Montevideo government. Second: continue to hunt down Morabito, wherever he is, to throw him in jail as he deserves," Salvini wrote on Twitter.

Interpol has issued a red notice — its highest-priority international arrest warrant — for all four escapees.

National

A black woman was beaten by a white man with a gun. Police charged her with damaging his truck.

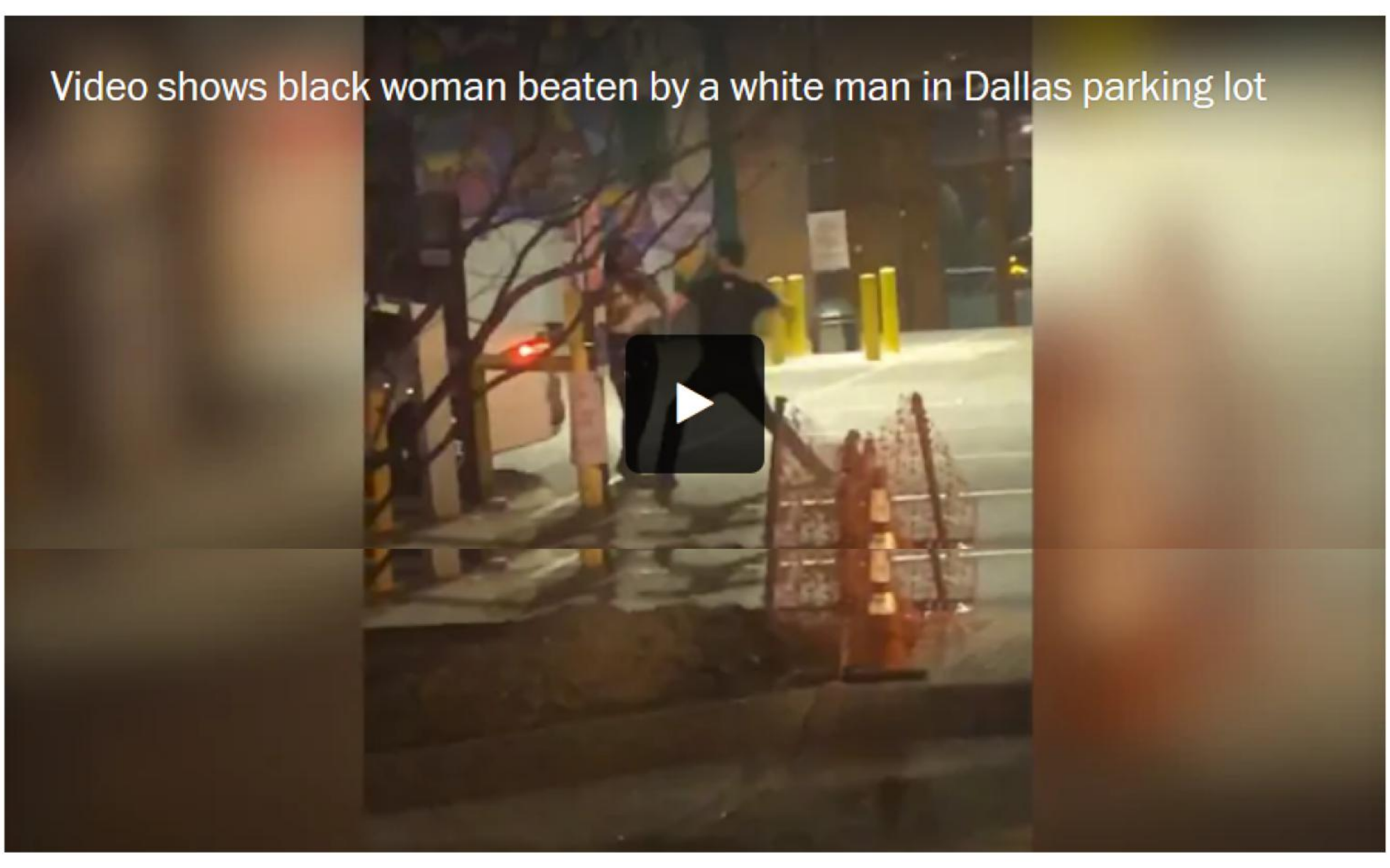

Dallas police charged L'Daijohnique Lee with felony criminal mischief for allegedly damaging Austin Shuffield's truck after he attacked her on March 21. (Courtesy of S. Lee Merritt)

By **Alex Horton**
April 3

On the night when a gun was pulled, punches were thrown and car windows smashed, transforming Dallas into the latest flash point of criminal justice and race relations, everything began with a traffic dispute.

L'Daijohnique Lee was going the wrong way down a one-way street in the Deep Ellum neighborhood on March 21. She was dropping off a friend, she said. Austin Shuffield, 30, was trying to leave a parking lot. He tried to take a picture of her license plate. Lee, 24, threatened to mace him if he didn't back away, WFAA reported, citing a police affidavit.

A bystander video captured what happened next. Shuffield, who is white, clutches a pistol at his side and steps toward Lee, who is black. She pulls out her phone to dial 911. Shuffield swats the phone to the ground, and Lee connects a punch. Then Shuffield winds up for at least five hard blows to Lee's head, sending her reeling. Then he kicks her phone down the street.

The video roared across social media and throughout Dallas, where protesters and community activists suggested Lee's race played a role in lesser misdemeanors for Shuffield because he is white, and urged more serious charges against Shuffield. But a felony charge landed first for Lee — the assault victim.

Lee was charged Tuesday with felony criminal mischief after allegedly smashing the windows of Shuffield's truck after the incident. That decision triggered more protests in Dallas, including one planned at city hall Wednesday, the Dallas Morning News reported, as the video and Lee's story spread.

On Wednesday, the Dallas County district attorney's office said Lee's warrant was recalled. "The case has been declined for prosecution," said Kimberlee Leach, a spokeswoman for the office. It was not immediately clear why.

The initial felony charge for Lee raised questions about whether it was appropriate for an assault victim.

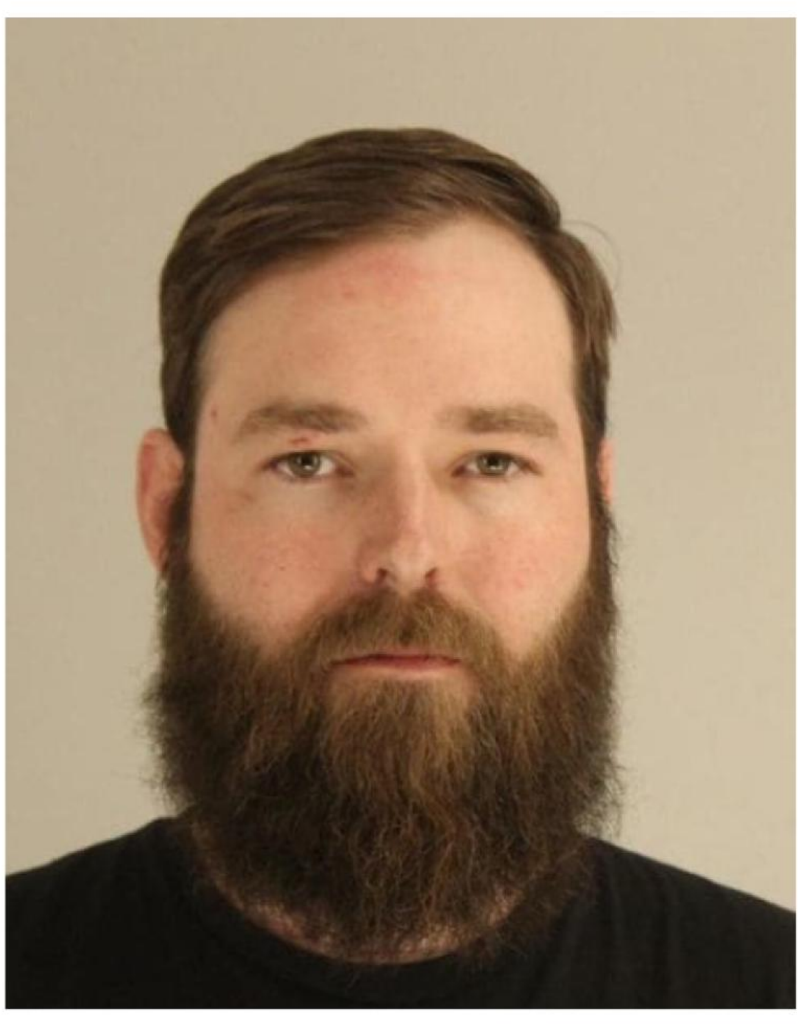

Austin Shuffield. (Dallas County Sheriff's Department)

"She's obviously in distress. You can't consider these things outside of context," said her attorney S. Lee Merritt, who criticized authorities for filing a felony charge against Lee before they focused on Shuffield.

Shuffield has been referred for a felony assault charge to the Dallas County district attorney's office to consider for a grand jury, police said. He has not been formally charged with any felonies.

"We understand that some people are upset," Dallas Deputy Police Chief Thomas Castro said in a news conference Tuesday, when he announced the now-dropped charges.

"It's not the intention of the Dallas Police Department to pick one side or the other. We simply had information that was provided to us on a criminal offense."

Police first charged Shuffield with public intoxication, interfering with an emergency call and assault — all misdemeanors.

But following a public outcry, an additional charge of unlawful carrying of a weapon was added on March 28. That is also a misdemeanor, Dallas police spokeswoman Sgt. Nicole Watson said. The recommendation for aggravated assault with a deadly weapon was made on the same day.

An attorney for Shuffield, J.R. Cook, declined to comment. Shuffield told detectives days later he feared for his life after Lee allegedly threatened to have friends shoot him, WFAA reported.

Lee also spoke about her fears from the encounter.

"All I could do was try to protect myself. He literally sat there and beat me like a man," Lee told WFAA soon after the incident.

At the news conference Tuesday, Castro was asked by a reporter if it was typical for crime victims to later be charged for what came after the crime.

He appeared to wince. "Each case is unique. Each case has its own set of circumstances," he said.

The Washington Post
Democracy Dies in Darkness

Woman sues McDonald's, alleges discrimination because she's transgender

Sarah Fowler, Mississippi Clarion Ledger Published 12:49 p.m. CT April 2, 2019 | Updated 1:15 p.m. CT April 2, 2019

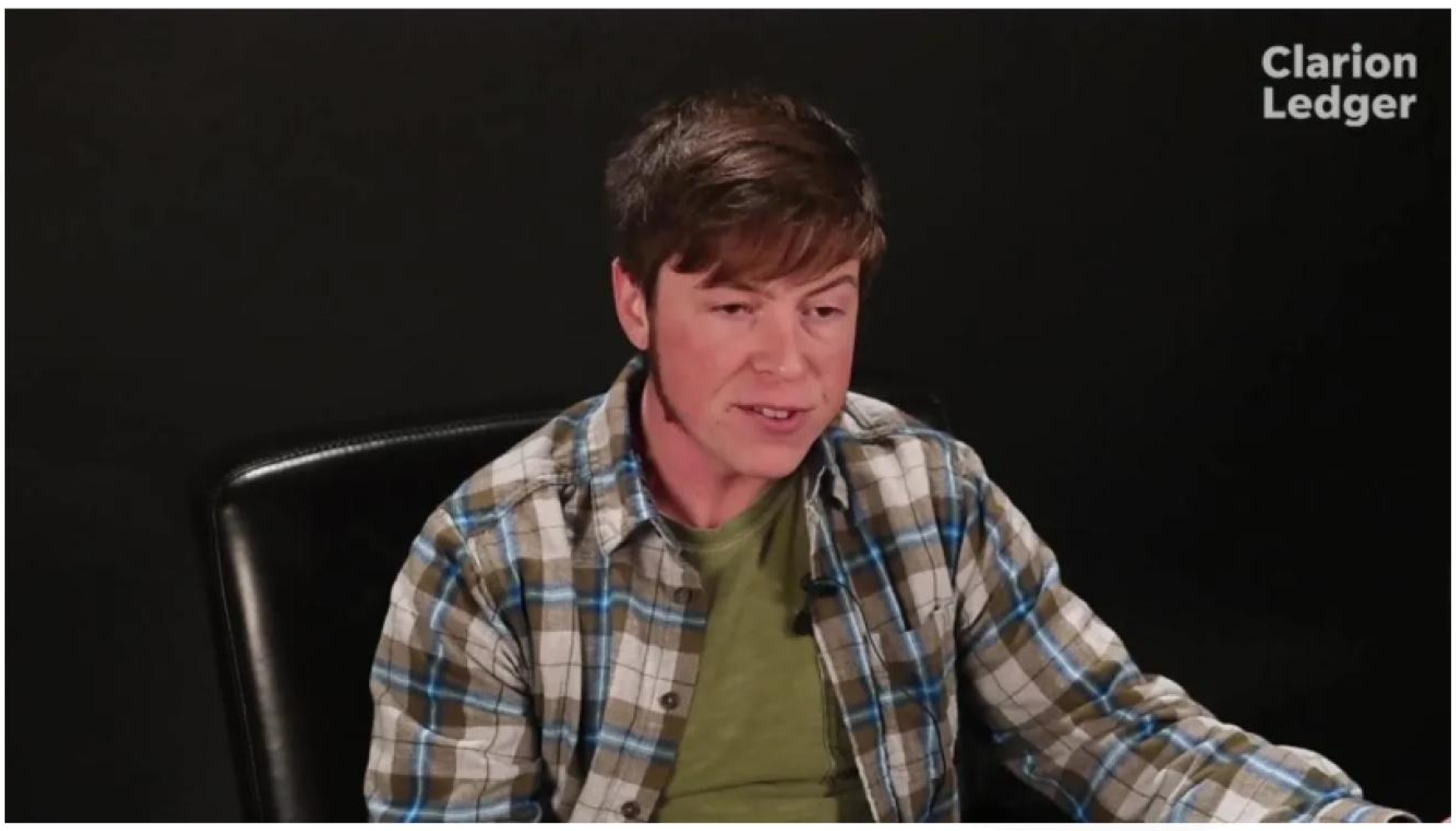

Several years ago, Ryan Anderson of Meridian, began the process of becoming himself. Sarah Warnock, Clarion Ledger

CONNECT TWEET LINKEDIN COMMENT EMAIL MORE

A Mississippi woman is suing McDonald's and a local franchise owner, claiming that she was subjected to repeated discrimination because she's transgender.

Earlier this year, Joselyn Kelly was hired by McDonald's franchise owners Elizabeth and Andrew Smith to work as an area supervisor for multiple McDonald's restaurants across North Mississippi, according to court documents.

She was hired on Jan. 4 with an anticipated start date of Jan. 8. On Jan. 7, according to the suit, Kelly informed the Smiths that she is transgender.

Shortly after she began working, the suit alleges, the Smiths "either directly or through their other employees began to discriminate against Joselyn."

Neither the Smiths nor Kelly's attorney, Charles Burchfield, could be immediately reached for comment. McDonald's did not respond to an emailed request for comment.
The suit only represents one side of a legal argument. It was not immediately known Tuesday if the Smiths had filed a response.

According to the suit, examples of the discrimination include:

- Refusing to address her by her preferred pronoun, calling her "he" or "him" rather than "she or "her";
- Addressing her as "it" and using a slur;
- Stating that "transgenders" were an abomination;
- Mockingly calling her "Juwanna Man," in reference to a male film character who impersonates a female;
- Assigning menial tasks to her that were only done by entry-level employees.

The actions were so traumatizing, the suit says, that Kelly was "forced to resign."

Kelly then filed a charge of discrimination against the Smiths with the Equal Employment Opportunity Commission. On March 20, the EEOC issued a notice of Kelly's right to sue. The suit was filed in the U.S. District Court for the Northern District of Mississippi on March 29.

Kelly is seeking compensatory damages, costs, including reasonable attorney's fees and any other equitable relief the court deems appropriate. She is also requesting a jury trial.

The New York Times

Mormons to allow baptisms for children of LGBTQ parents, won't expel those in same-sex marriages

Jorge L. Ortiz

Published 12:08 p.m. ET April 4, 2019 | **Updated 8:37 p.m. ET April 5, 2019**

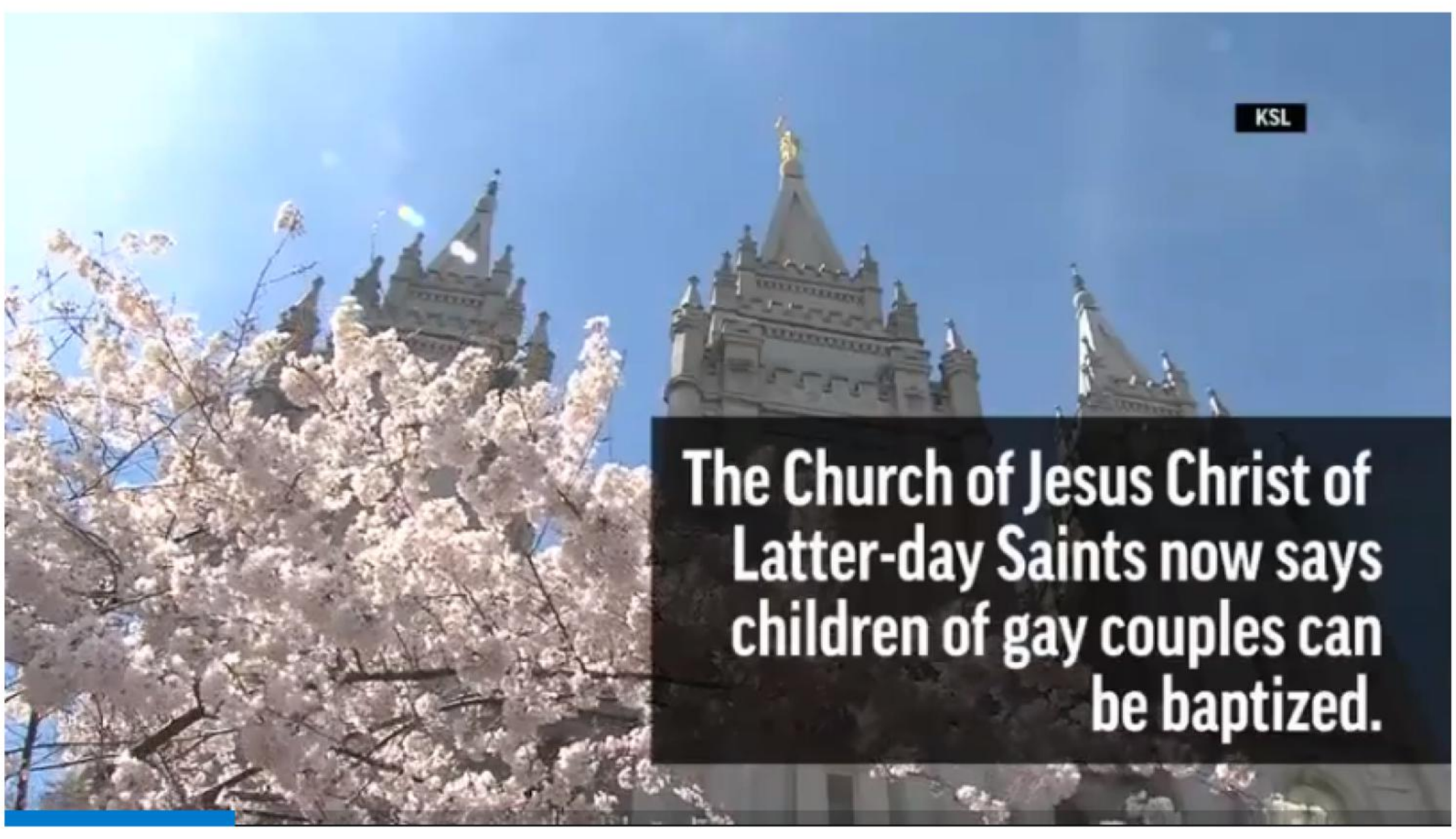

The Church of Jesus Christ of Latter-day Saints on Thursday repealed rules that banned baptisms for children of gay parents and that labeled same-sex couples as sinners eligible for expulsion. (April 5)

Gay Mormons won't be kicked out of the church anymore, and they'll be allowed to baptize their kids again.

In a surprise reversal of controversial rules established four years ago, the Church of Jesus Christ of Latter-day Saints announced Thursday that gay marriage will no longer be considered a sin worthy of expulsion.

A 2015 policy that deemed those in same-sex relationships "apostates" who must be banished from the religion drew strong opposition and condemnation from the LGBTQ community and its supporters.

Under the change, children of gay parents can be baptized without special permission as long as their parents approve and acknowledge that the children will be taught Mormon doctrine, the church said in a statement from its three-person governing body, known as the First Presidency.

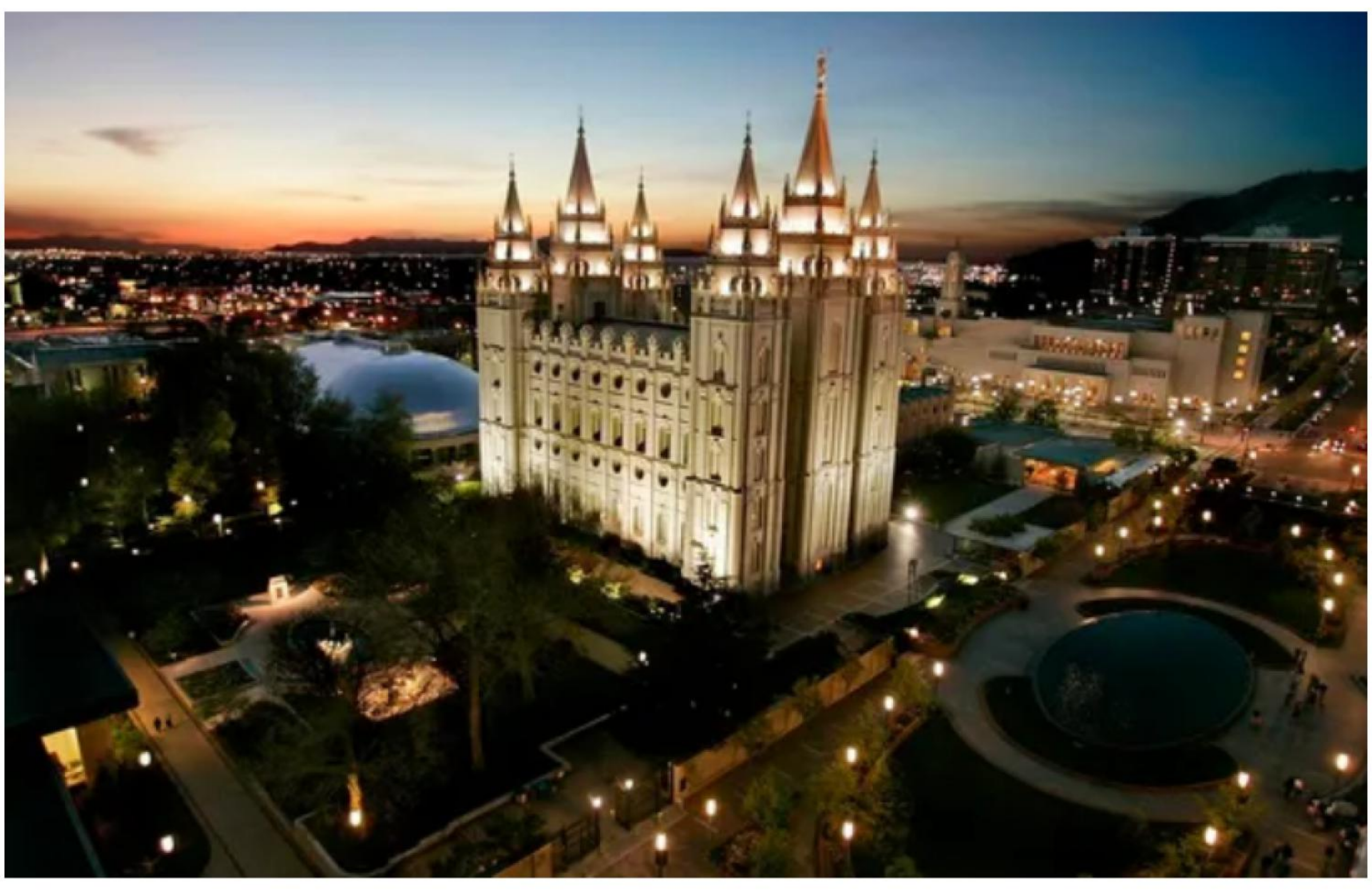

The sun sets behind the Mormon Temple, the centerpiece of Temple Square, in Salt Lake City.

First counselor "Dallin H. Oaks instructed that the Gospel of Jesus Christ teaches us to love and treat all people with kindness and civility – even when we disagree," said the statement from the church's general conference in Salt Lake City.

The church said it's not changing its doctrinal opposition to gay marriage and still regards same-sex relationships as a "serious transgression." There was no apology for the previous policy from leaders of a faith that has about 16 million followers globally.

"The very positive policies announced this morning should help affected families," the statement said. "In addition, our members' efforts to show more understanding, compassion and love should increase respect and understanding among all people of goodwill."

The reversal from the stricter rules is the latest in a series of changes by church President Russell Nelson since he assumed the leadership in January 2018.

The Human Rights Campaign, the largest LGBTQ rights organization in the country, was among those greeting the change warmly.

"There's still work to do," HRC President Chad Griffin said. "But this policy reversal is a very welcome change that moves the church closer to a day where LGBTQ Mormons can see themselves affirmed and included within their faith community."

Sam Brinton, head of advocacy and government affairs for the Trevor Project – a suicide prevention and crisis intervention organization for LGBTQ young people – said his group regularly hears from gay youths struggling to reconcile their faith with their sexual orientation.

"The Trevor Project welcomes any faith group's public commitment to treat the LGBTQ community fairly and equally, and this statement by the LDS Church to change course is a move in the right direction that will make a real difference in the lives of LGBTQ Mormons," Brinton said.

Patrick Mason, chair of Mormon studies at Claremont Graduate University in California, said the church hurt its image and relationship with many members – both conservative and liberal – with its 2015 policy, which ran counter to a movement toward more openness on LGBTQ issues.

Troy Williams, executive director of the advocacy group Equality Utah, was among those who viewed the new policy as a positive step.

"Clearly this is a great development for the church," he said. "I think this will go a long way toward healing Latter-day Saint families that have LGBT members."

Real News Articles with Non-Credible News Source

A passerby heard a newborn crying in a storm drain. Four hours later, she was rescued.

By Gianluca Mezzofiore
Updated 11:32 AM ET, Tue February 12, 2019

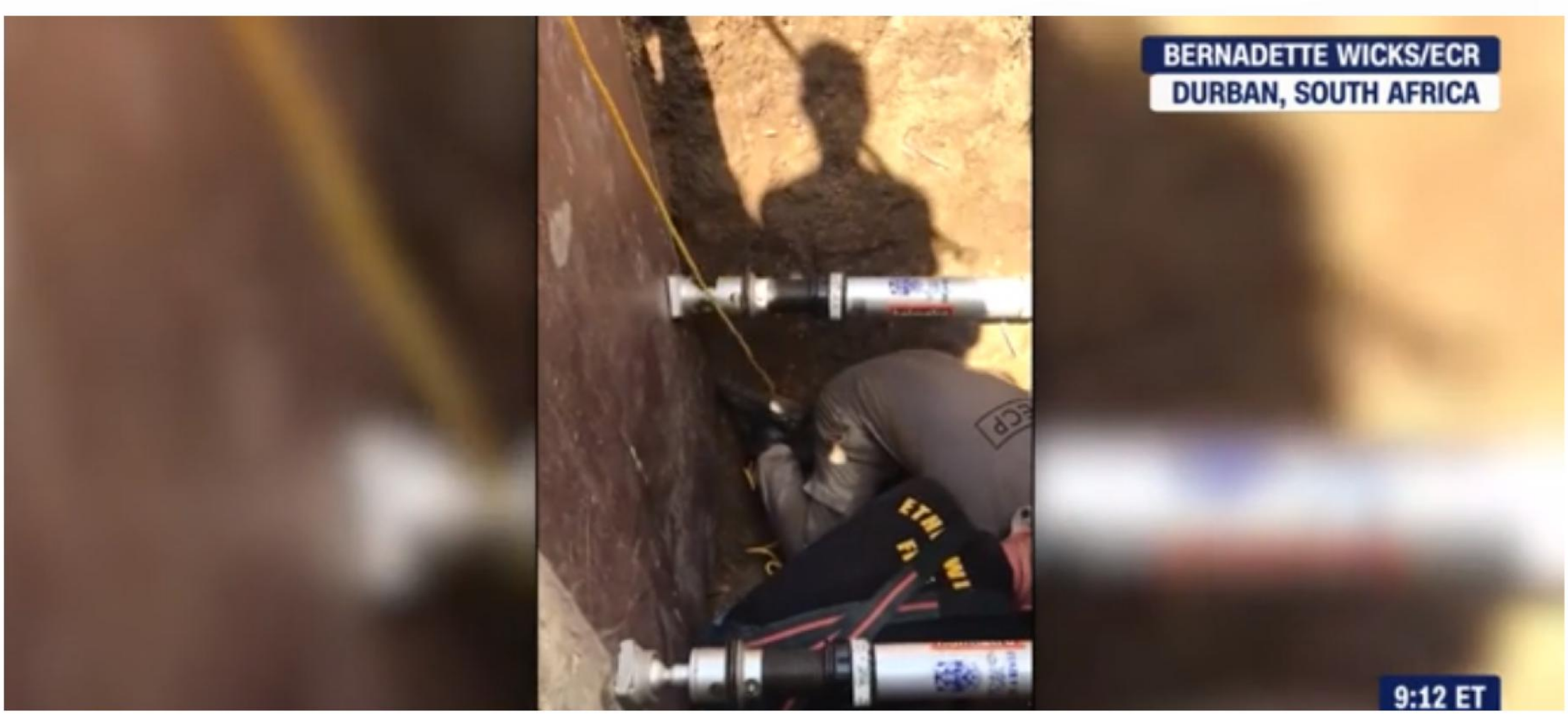

A baby girl was rescued from a storm drain in Durban, South Africa, after residents heard her crying and alerted emergency services.

A bystander had spotted the newborn after climbing down into the drain on Monday, Garrith Jamieson, head of Durban Rescue Care, said in a statement obtained by CNN.

Rescue services used a chisel and hammer to break into the drain, Jamieson said. The whole operation lasted nearly four hours.

After being located and extricated, the baby was flown to Inkosi Albert Luthuli Hospital for urgent care.

Dramatic video from the scene showed the moment the baby was lifted to safety.

Bernadette Wicks (Wolhuter)
@bern_wicks

The incredible moment a newborn baby girl is rescued from more than seven metres down a storm water pipe. The amazing men & women from Durban's emergency services have been working for nearly three hours to save the infant. #NewlandsEastBabyRescue @ECR_Newswatch

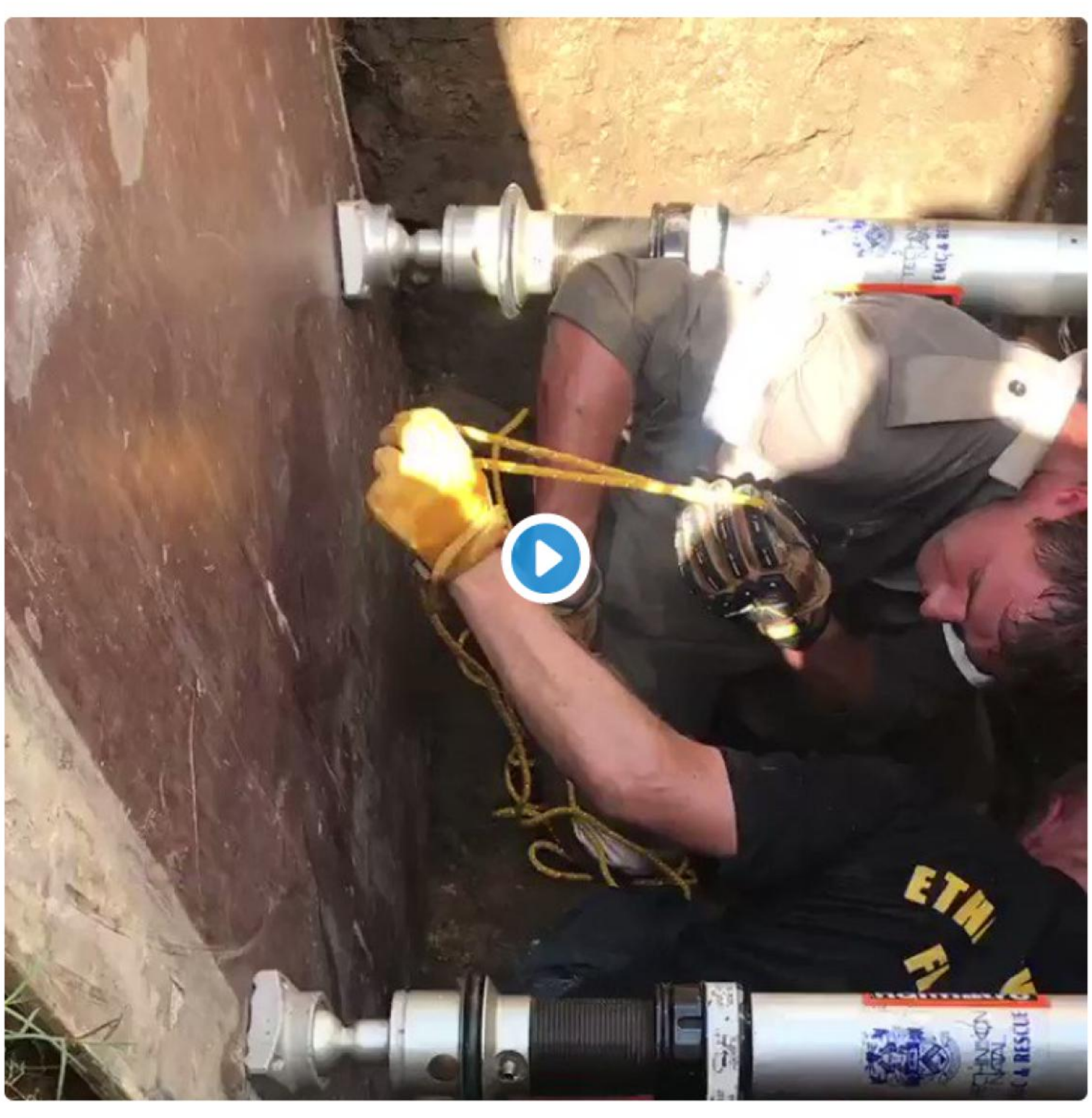

1,559 3:29 AM - Feb 11, 2019

895 people are talking about this

The hospital's trauma specialist, Dr Timothy Hardcastle, said the baby was suffering from hypothermia and had to be warmed up, but was in satisfactory condition with only minor injuries, according to a statement from the KwaZulu-Natal (KZN) province department of health.

The baby, who was found with her umbilical cord intact, was believed to be between one and three days old.

She was named Sibanisethu (Our Ray of Light) and Gabriella by residents, the statement said.

Dr Sibongiseni Dhlomo, MP
about 5 months ago

MEDIA RELEASE:
MIRACULOUS BABY RESCUE: MEC DHLOMO SAYS BABY ABANDONMENT IS A SIGN THAT PROGRAMMES PROMOTING CONTRACEPTIVES, CONDOMS NEEDS TO BE STRENGTHENED
11 February 2019... See More

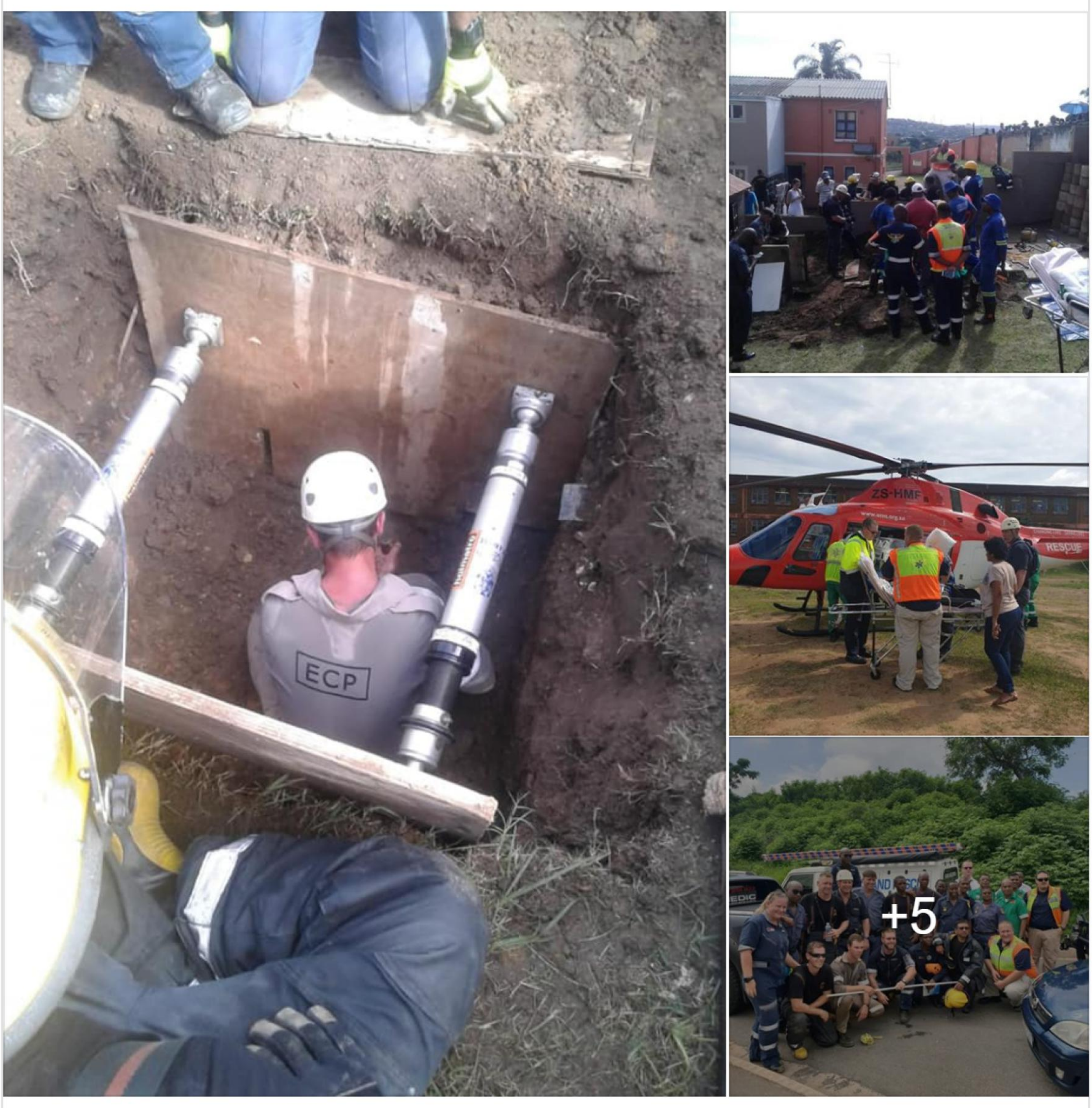

27 7 13

"It's really a miracle that this baby was saved. The doctors have given this baby a clean bill of health, and she will be transferred to another hospital," said regional health official Dr. Sibongiseni Dhlomo.

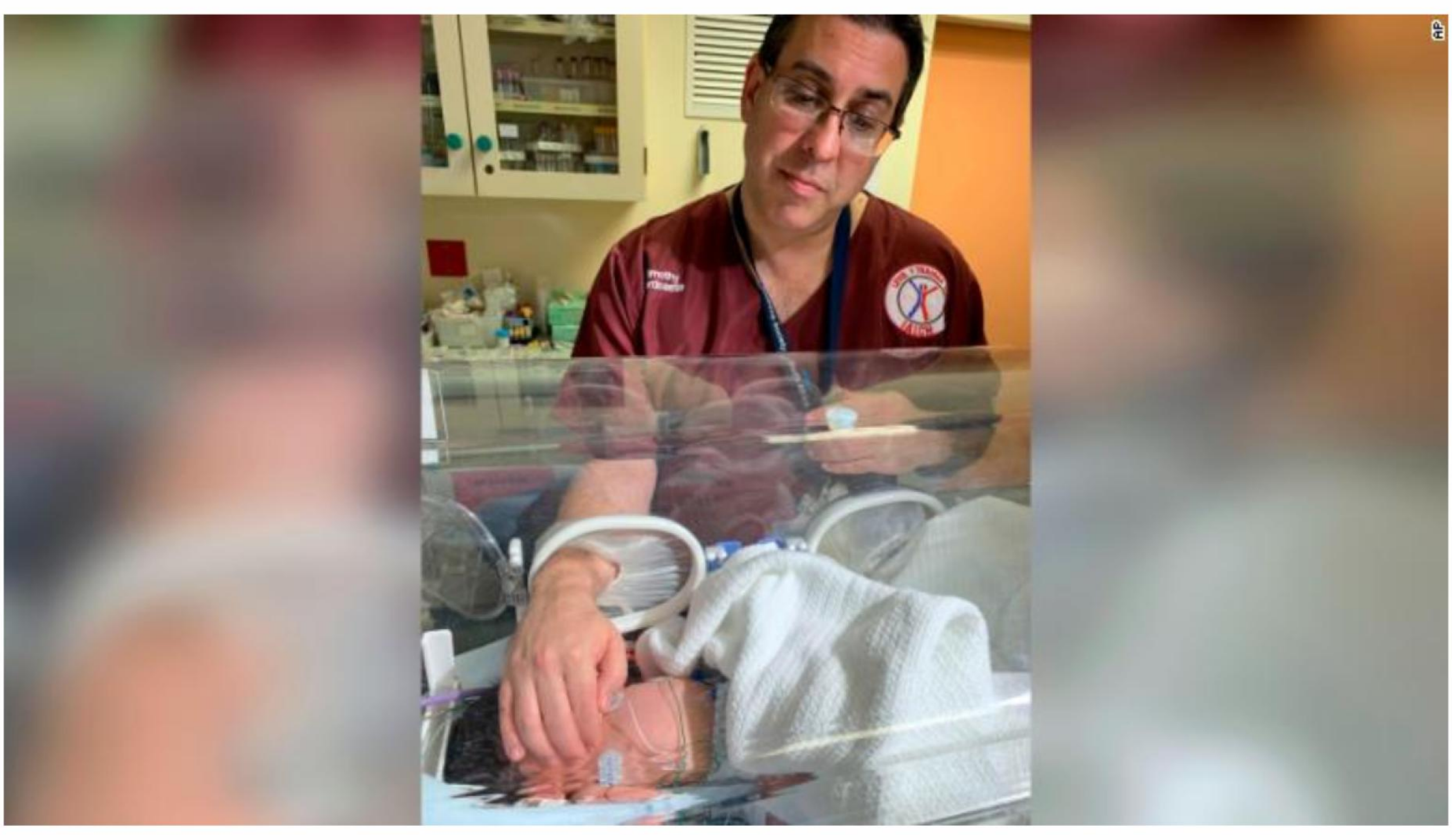

Doctor Timothy Hardcastle treats a baby in the hospital after she was rescued from a storm water pipe in Durban, South Africa.

KZN police spokesperson Col. Thembeka Mbele said a case of attempted murder was opened at Newlands East police station, according to Times Live. Police are investigating how the newborn ended up in the storm drain.

CDN
News for the People,
by the People

Texas Senate passes bill allowing school marshals to carry, not lock up, their guns

Eliminating the lockbox requirement for school marshals was one of many suggestions Gov. Greg Abbott outlined for the Legislature in a 43-page plan released weeks after last year's deadly shooting at Santa Fe High School.

BY ALEX SAMUELS APRIL 3, 2019

REPUBLISH

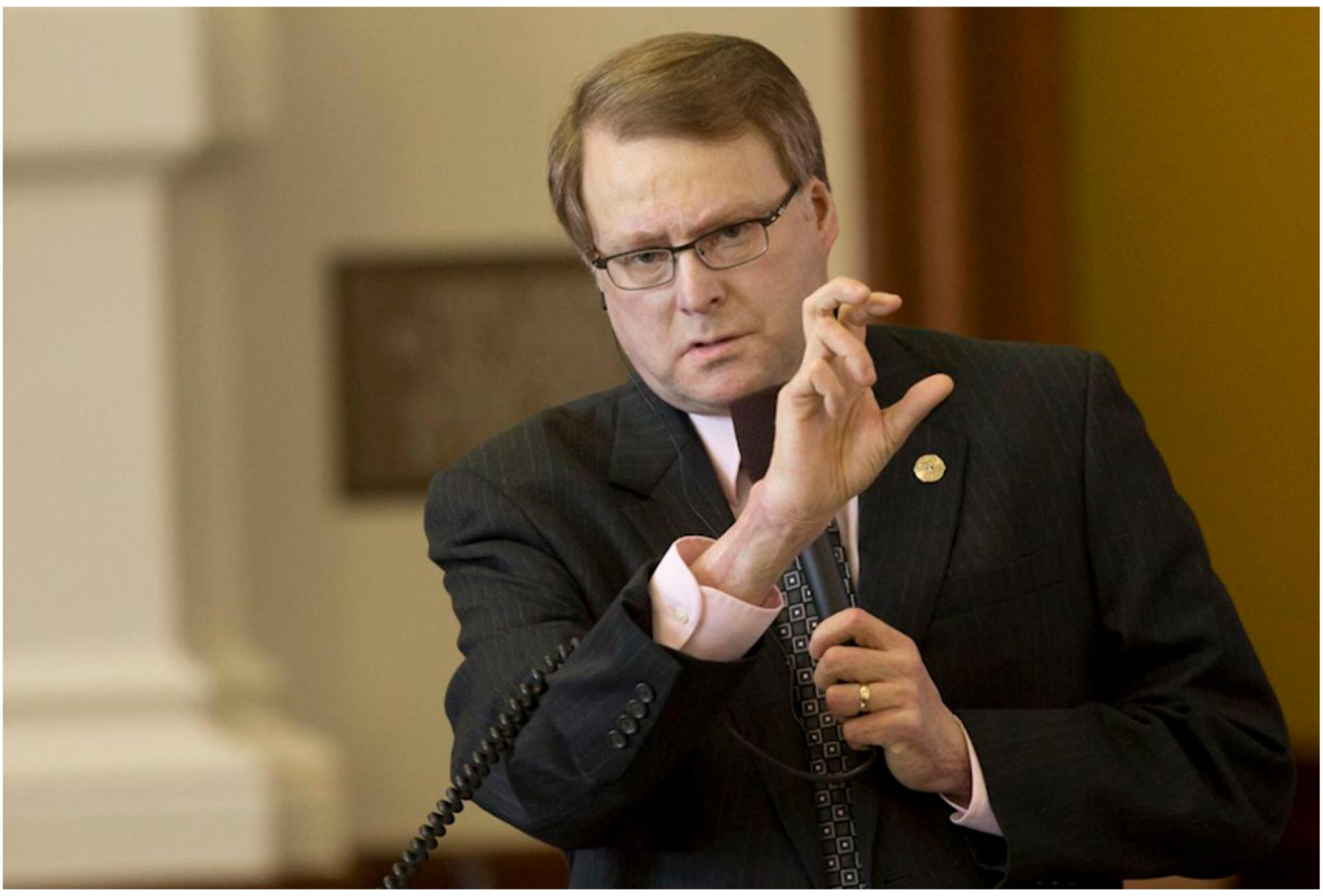

Senate Bill 406, by Republican state Sen. Brian Birdwell of Granbury, would eliminate the mandate that trained school marshals keep their firearms under lock and key. Marjorie Kamys Cotera for The Texas Tribune

After a brief debate, the Texas Senate approved a bill Wednesday that would allow local school boards to let their marshals carry their concealed guns on campuses.

The legislation — Senate Bill 406 by Republican state Sen. Brian Birdwell of Granbury — would eliminate the mandate that trained school marshals, whose identities are kept secret from all but a few local officials, keep their firearms under lock and key.

More specifically, it would give the board of trustees or the governing body of public schools, open-enrollment charter schools, private schools and junior colleges the discretion to decide whether their marshals carry their weapons on their person or in a locked and secured safe.

The measure passed 28-3, with Democratic state Sens. José Menéndez of San Antonio, José Rodríguez of El Paso and Kirk Watson of Austin voting against it. The bill can now be sent to the Texas House for debate.

“The code we’re currently dealing with specifically requires that those individuals who are designated as school marshals store their handguns in a locked box at all times,” Birdwell told other senators. “While a marshal would be acting in accordance with this state law, the practicality of this security is nullified if a school marshal cannot protect their students at the critical time and the critical place.”

To be clear, SB 406 does not compel school districts to implement a marshal program. Nor does it compel local districts to force marshals to openly carry their weapons.

“School districts retain the discretion to decide whether to implement the school marshal program fully, not at all or to some degree,” Birdwell said.

Eliminating the lockbox requirement for school marshals was one of many suggestions Gov. Greg Abbott outlined for the Legislature in a 43-page plan released weeks after a deadly shooting at Santa Fe High School in May that left 10 dead and 13 others wounded.

Gun control advocates, meanwhile, criticized the passage of the bill. In a statement, the Texas chapter of Moms Demand Action for Gun Sense in America, said SB 406 “would make school a much more dangerous place for our children.”

“It’s baffling that lawmakers are trying to strip gun storage standards in our schools,” wrote Hilary Whitfield, volunteer leader with the Texas chapter of Moms Demand Action for Gun Sense in America. “That is a recipe for disaster.”

During a brief floor debate, some Democrats echoed these worries.

'Cocaine King Of Milan' On The Run After Uruguay Jailbreak

June 25, 2019 · 6:41 PM ET

VANESSA ROMO

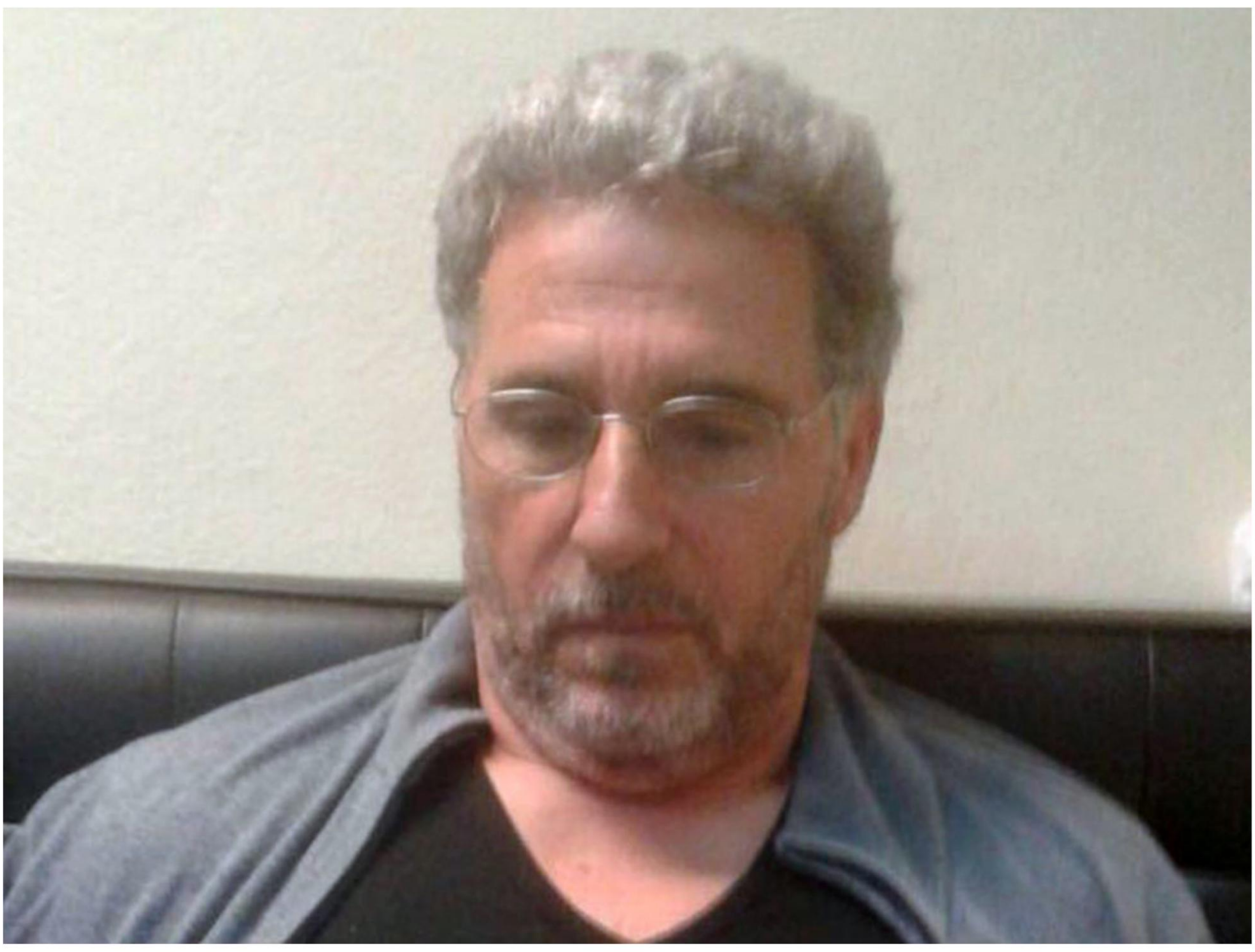

Rocco Morabito, pictured after his arrest in 2017, escaped from the Uruguayan prison where he was awaiting extradition to Italy.

Italian Police via AP

Uruguayan officials have launched a manhunt for an Italian organized crime boss known as the Cocaine King of Milan who escaped on Sunday from a detention center where he was awaiting extradition to Italy, the Uruguayan Ministry of Interior said in a statement.

Rocco Morabito and three other inmates made a brazen escape from the prison in Montevideo, climbing through a hole in the roof of the building. Reports from ministry officials indicated that the men eventually broke into a neighboring property, robbed the owner, then fled.

But *El Observador* reports that one of the four men, a Brazilian also waiting to be extradited to his home country, actually avoided the hassle of the daring jailbreak by simply walking through a side door of the building "without anyone stopping him."

Morabito, who is believed to be the son of another famed mobster with the same name, was one of the 10 most wanted criminals in the world in 2017 as the leader of the Calabrian 'Ndrangheta — one of Italy's most powerful organized crime groups.

A 2013 Europol report found the 'Ndrangheta "now recognised as a major threat not only in Italy but also in many other countries where it operates, including Germany, Spain, the Netherlands, France, Belgium, Switzerland, Canada, US, Colombia and Australia."

The drug kingpin was sentenced in absentia to 30 years in prison in Italy. Authorities have been chasing him since he was caught trying to import nearly a ton of cocaine into the country from Brazil in 1994.

Morabito had been living on the run for more than two decades under a false identity but was eventually captured after trying to enroll his daughter in a school using his real name.

El Observador reported the director of prisons has resigned following Sunday's escape.

According to the newspaper, officials were warned a year ago about an escape plan hatched by Morabito bearing an eerie resemblance to the weekend's events. The only major difference between the 2018 plan and what happened around midnight Sunday is that in the earlier version Morabito was set to escape from the sixth floor of the penitentiary onto the roof of a supermarket — not a neighboring apartment building.

Italy's minister of the interior, Matteo Salvini, was furious over the breakout.

"I make two commitments. First: to shed light on the methods of evasion, asking for immediate explanations from the Montevideo government. Second: continue to hunt down Morabito, wherever he is, to throw him in jail as he deserves," Salvini wrote on Twitter.

Interpol has issued a red notice — its highest-priority international arrest warrant — for all four escapees.

National

A black woman was beaten by a white man with a gun. Police charged her with damaging his truck.

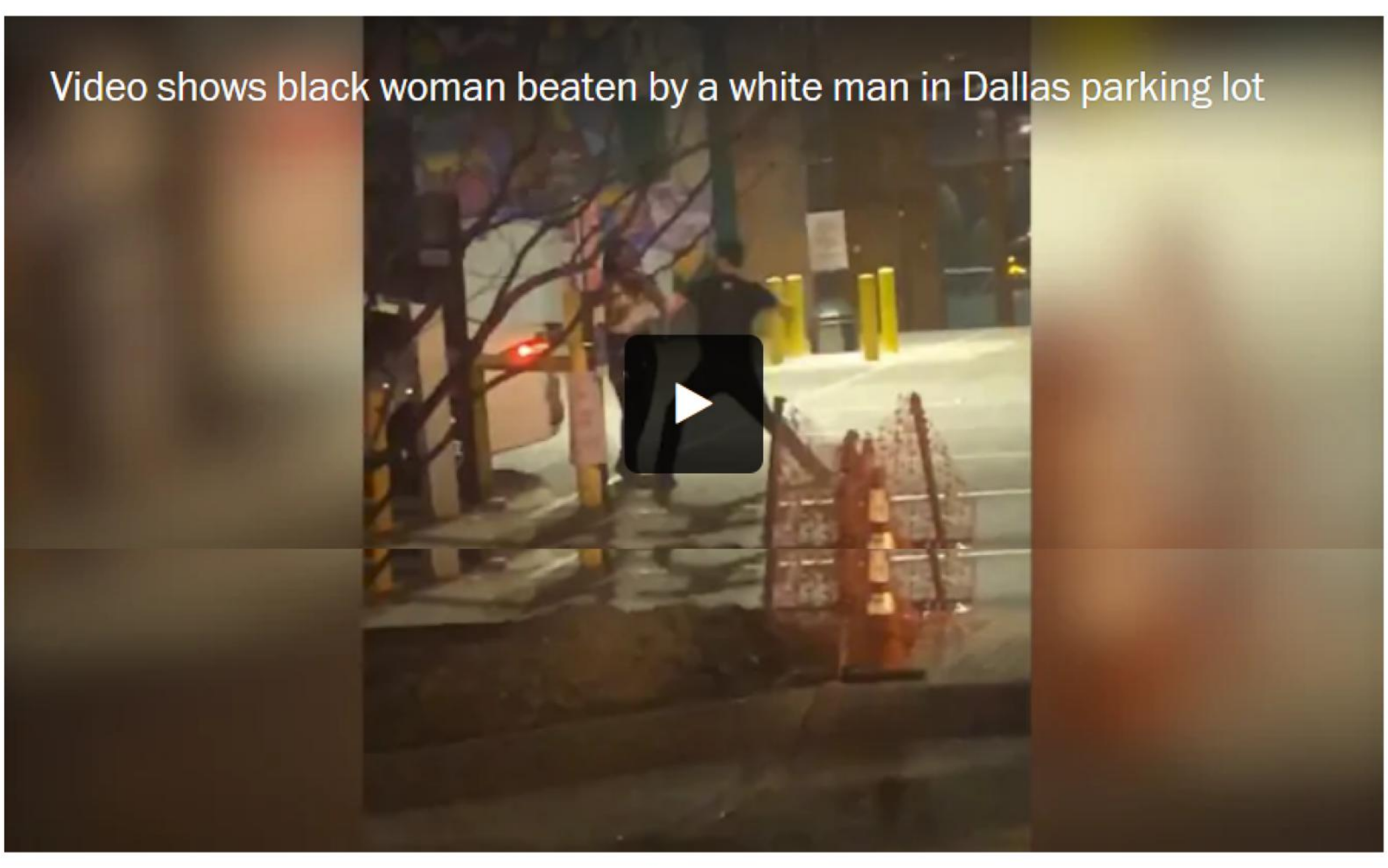

Dallas police charged L'Daijohnique Lee with felony criminal mischief for allegedly damaging Austin Shuffield's truck after he attacked her on March 21. (Courtesy of S. Lee Merritt)

By **Alex Horton**
April 3

On the night when a gun was pulled, punches were thrown and car windows smashed, transforming Dallas into the latest flash point of criminal justice and race relations, everything began with a traffic dispute.

L'Daijohnique Lee was going the wrong way down a one-way street in the Deep Ellum neighborhood on March 21. She was dropping off a friend, she said. Austin Shuffield, 30, was trying to leave a parking lot. He tried to take a picture of her license plate. Lee, 24, threatened to mace him if he didn't back away, WFAA reported, citing a police affidavit.

A bystander video captured what happened next. Shuffield, who is white, clutches a pistol at his side and steps toward Lee, who is black. She pulls out her phone to dial 911. Shuffield swats the phone to the ground, and Lee connects a punch. Then Shuffield winds up for at least five hard blows to Lee's head, sending her reeling. Then he kicks her phone down the street.

The video roared across social media and throughout Dallas, where protesters and community activists suggested Lee's race played a role in lesser misdemeanors for Shuffield because he is white, and urged more serious charges against Shuffield. But a felony charge landed first for Lee — the assault victim.

Lee was charged Tuesday with felony criminal mischief after allegedly smashing the windows of Shuffield's truck after the incident. That decision triggered more protests in Dallas, including one planned at city hall Wednesday, the Dallas Morning News reported, as the video and Lee's story spread.

On Wednesday, the Dallas County district attorney's office said Lee's warrant was recalled. "The case has been declined for prosecution," said Kimberlee Leach, a spokeswoman for the office. It was not immediately clear why.

The initial felony charge for Lee raised questions about whether it was appropriate for an assault victim.

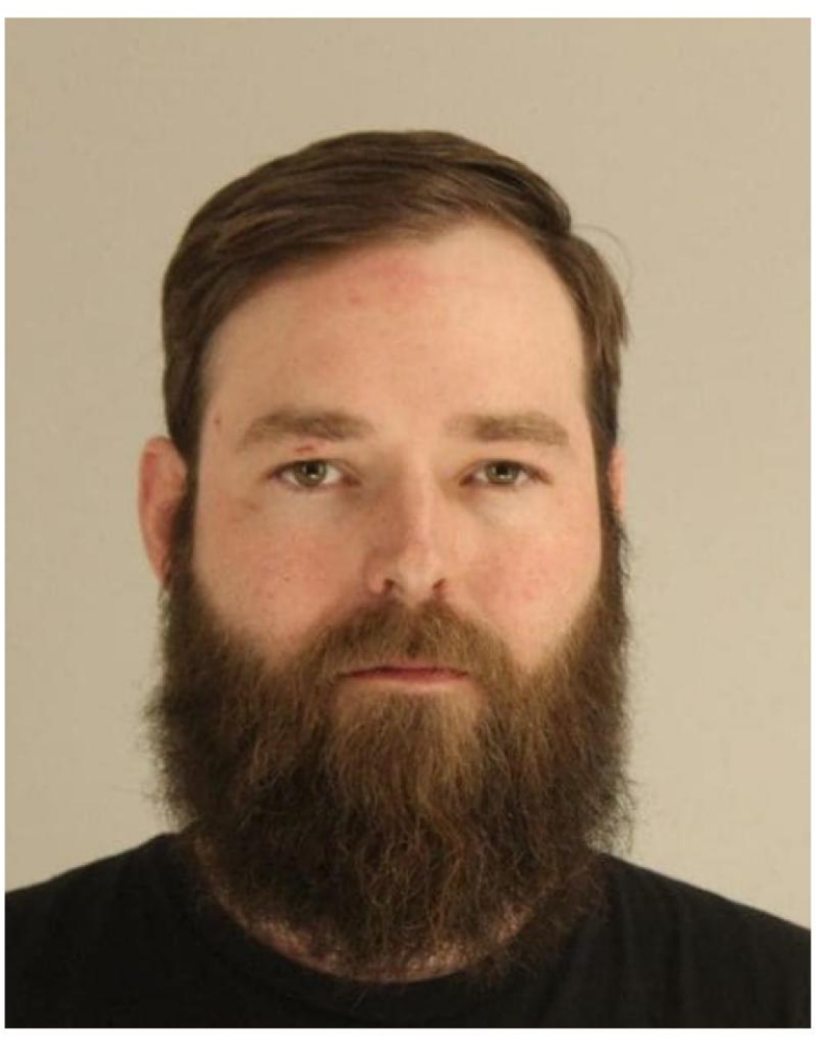

Austin Shuffield. (Dallas County Sheriff's Department)

"She's obviously in distress. You can't consider these things outside of context," said her attorney S. Lee Merritt, who criticized authorities for filing a felony charge against Lee before they focused on Shuffield.

Shuffield has been referred for a felony assault charge to the Dallas County district attorney's office to consider for a grand jury, police said. He has not been formally charged with any felonies.

"We understand that some people are upset," Dallas Deputy Police Chief Thomas Castro said in a news conference Tuesday, when he announced the now-dropped charges.

"It's not the intention of the Dallas Police Department to pick one side or the other. We simply had information that was provided to us on a criminal offense."

Police first charged Shuffield with public intoxication, interfering with an emergency call and assault — all misdemeanors.

But following a public outcry, an additional charge of unlawful carrying of a weapon was added on March 28. That is also a misdemeanor, Dallas police spokeswoman Sgt. Nicole Watson said. The recommendation for aggravated assault with a deadly weapon was made on the same day.

An attorney for Shuffield, J.R. Cook, declined to comment. Shuffield told detectives days later he feared for his life after Lee allegedly threatened to have friends shoot him, WFAA reported.

Lee also spoke about her fears from the encounter.

"All I could do was try to protect myself. He literally sat there and beat me like a man," Lee told WFAA soon after the incident.

At the news conference Tuesday, Castro was asked by a reporter if it was typical for crime victims to later be charged for what came after the crime.

He appeared to wince. "Each case is unique. Each case has its own set of circumstances," he said.

Woman sues McDonald's, alleges discrimination because she's transgender

Sarah Fowler, Mississippi Clarion Ledger Published 12:49 p.m. CT April 2, 2019 | Updated 1:15 p.m. CT April 2, 2019

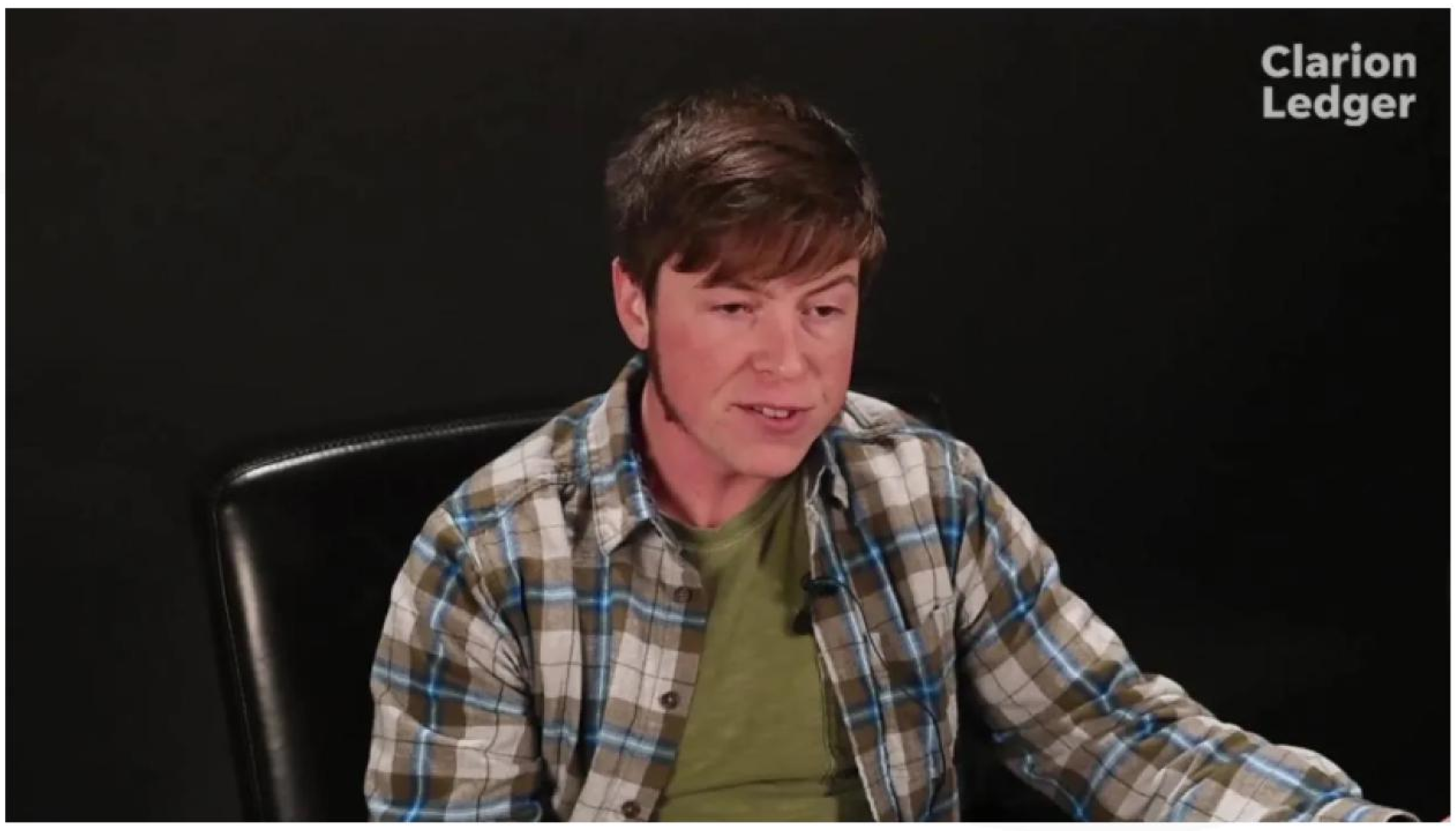

Several years ago, Ryan Anderson of Meridian, began the process of becoming himself. Sarah Warnock, Clarion Ledger

CONNECT TWEET LINKEDIN COMMENT EMAIL MORE

A Mississippi woman is suing McDonald's and a local franchise owner, claiming that she was subjected to repeated discrimination because she's transgender.

Earlier this year, Joselyn Kelly was hired by McDonald's franchise owners Elizabeth and Andrew Smith to work as an area supervisor for multiple McDonald's restaurants across North Mississippi, according to court documents.

She was hired on Jan. 4 with an anticipated start date of Jan. 8. On Jan. 7, according to the suit, Kelly informed the Smiths that she is transgender.

Shortly after she began working, the suit alleges, the Smiths "either directly or through their other employees began to discriminate against Joselyn."

Neither the Smiths nor Kelly's attorney, Charles Burchfield, could be immediately reached for comment. McDonald's did not respond to an emailed request for comment.
The suit only represents one side of a legal argument. It was not immediately known Tuesday if the Smiths had filed a response.

According to the suit, examples of the discrimination include:

- Refusing to address her by her preferred pronoun, calling her "he" or "him" rather than "she or "her";
- Addressing her as "it" and using a slur;
- Stating that "transgenders" were an abomination;
- Mockingly calling her "Juwanna Man," in reference to a male film character who impersonates a female;
- Assigning menial tasks to her that were only done by entry-level employees.

The actions were so traumatizing, the suit says, that Kelly was "forced to resign."

Kelly then filed a charge of discrimination against the Smiths with the Equal Employment Opportunity Commission. On March 20, the EEOC issued a notice of Kelly's right to sue. The suit was filed in the U.S. District Court for the Northern District of Mississippi on March 29.

Kelly is seeking compensatory damages, costs, including reasonable attorney's fees and any other equitable relief the court deems appropriate. She is also requesting a jury trial.

TRUE PUNDIT

Mormons to allow baptisms for children of LGBTQ parents, won't expel those in same-sex marriages

Jorge L. Ortiz   Published 12:08 p.m. ET April 4, 2019 | **Updated 8:37 p.m. ET April 5, 2019**

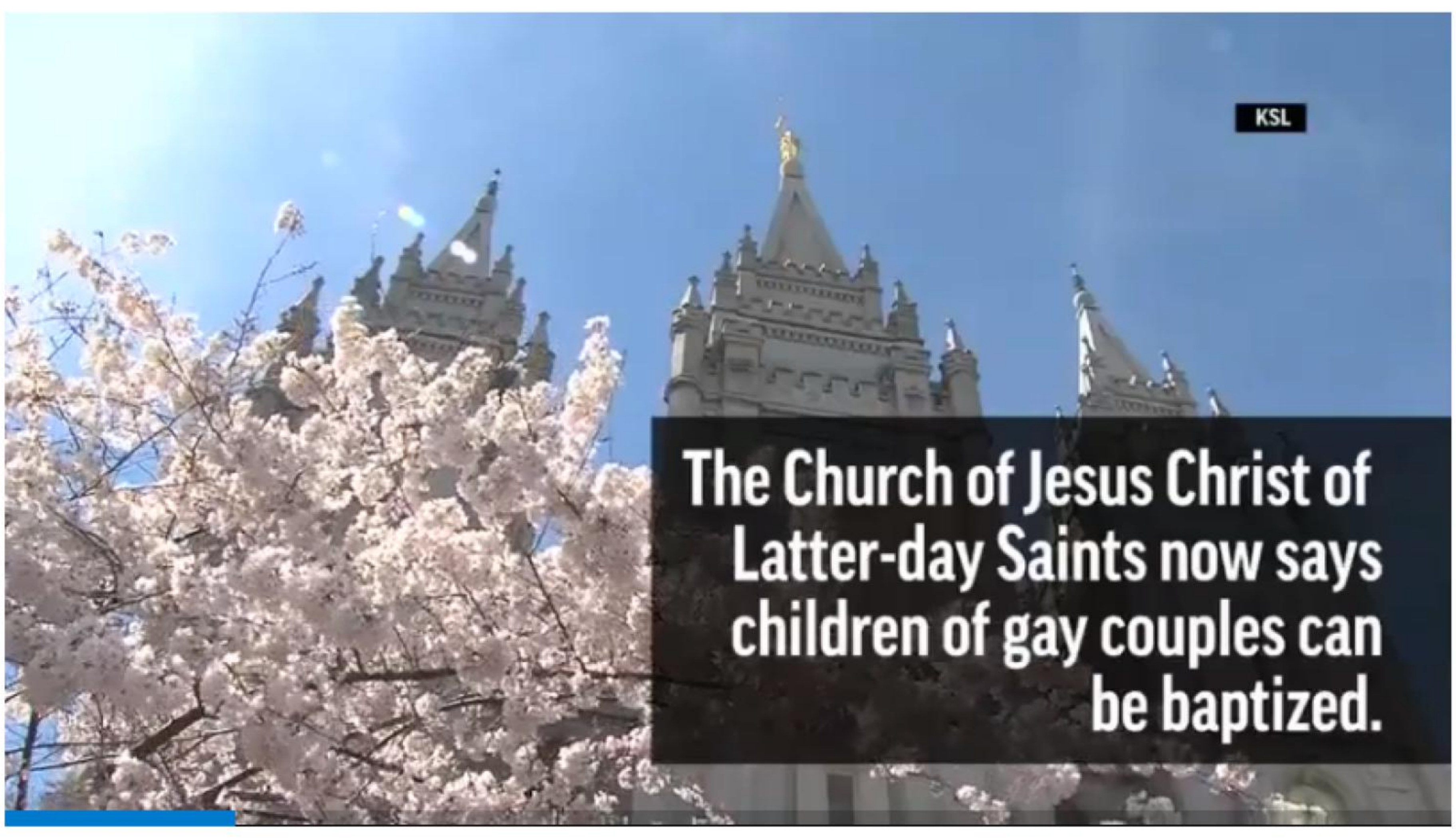

The Church of Jesus Christ of Latter-day Saints on Thursday repealed rules that banned baptisms for children of gay parents and that labeled same-sex couples as sinners eligible for expulsion. (April 5)

Gay Mormons won't be kicked out of the church anymore, and they'll be allowed to baptize their kids again.

In a surprise reversal of controversial rules established four years ago, the Church of Jesus Christ of Latter-day Saints announced Thursday that gay marriage will no longer be considered a sin worthy of expulsion.

A 2015 policy that deemed those in same-sex relationships "apostates" who must be banished from the religion drew strong opposition and condemnation from the LGBTQ community and its supporters.

Under the change, children of gay parents can be baptized without special permission as long as their parents approve and acknowledge that the children will be taught Mormon doctrine, the church said in a statement from its three-person governing body, known as the First Presidency.

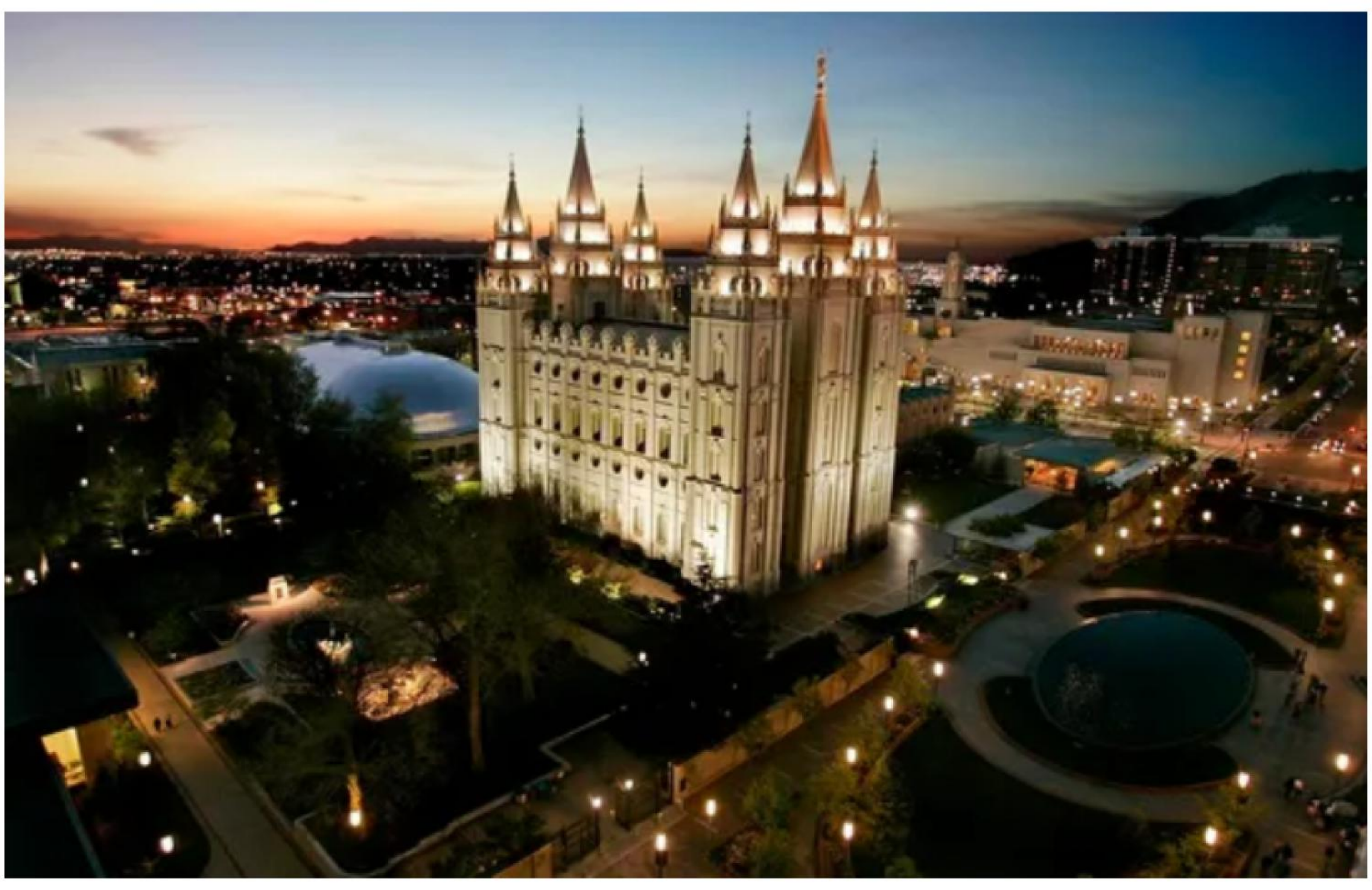

The sun sets behind the Mormon Temple, the centerpiece of Temple Square, in Salt Lake City.

First counselor "Dallin H. Oaks instructed that the Gospel of Jesus Christ teaches us to love and treat all people with kindness and civility – even when we disagree," said the statement from the church's general conference in Salt Lake City.

The church said it's not changing its doctrinal opposition to gay marriage and still regards same-sex relationships as a "serious transgression." There was no apology for the previous policy from leaders of a faith that has about 16 million followers globally.

"The very positive policies announced this morning should help affected families," the statement said. "In addition, our members' efforts to show more understanding, compassion and love should increase respect and understanding among all people of goodwill."

The reversal from the stricter rules is the latest in a series of changes by church President Russell Nelson since he assumed the leadership in January 2018.

The Human Rights Campaign, the largest LGBTQ rights organization in the country, was among those greeting the change warmly.

"There's still work to do," HRC President Chad Griffin said. "But this policy reversal is a very welcome change that moves the church closer to a day where LGBTQ Mormons can see themselves affirmed and included within their faith community."

Sam Brinton, head of advocacy and government affairs for the Trevor Project – a suicide prevention and crisis intervention organization for LGBTQ young people – said his group regularly hears from gay youths struggling to reconcile their faith with their sexual orientation.

"The Trevor Project welcomes any faith group's public commitment to treat the LGBTQ community fairly and equally, and this statement by the LDS Church to change course is a move in the right direction that will make a real difference in the lives of LGBTQ Mormons," Brinton said.

Patrick Mason, chair of Mormon studies at Claremont Graduate University in California, said the church hurt its image and relationship with many members – both conservative and liberal – with its 2015 policy, which ran counter to a movement toward more openness on LGBTQ issues.

Troy Williams, executive director of the advocacy group Equality Utah, was among those who viewed the new policy as a positive step.

"Clearly this is a great development for the church," he said. "I think this will go a long way toward healing Latter-day Saint families that have LGBT members."

Fake News Articles with Credible News Source

The Washington Post
Democracy Dies in Darkness

News

New law makes it legal for atheist doctors and nurses to refuse care to religious patients

By SP Team on January 19, 2018

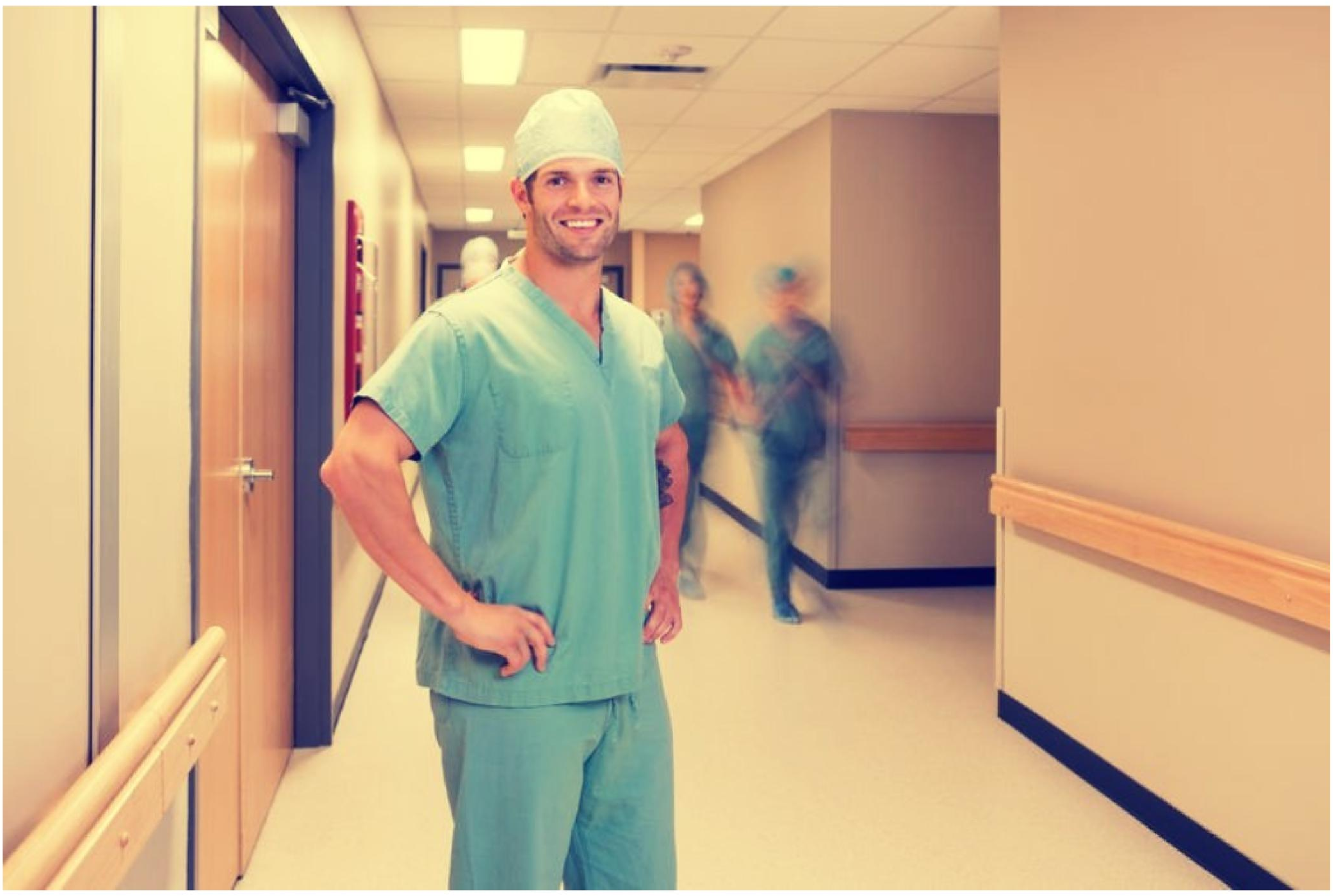

170.3k SHARES Share Tweet

JACKSON, MS – A new law in Mississippi has made it legal for doctors and nurses to refuse care to certain patients on religious grounds .

The law allows medical staff to refuse treatment to members of the LGBQT community on the grounds that it violates their religious beliefs. It would serve to reason this would also pertain to atheist doctors and nurses who could refuse to treat patients who are religious.

"No, no, no. This isn't the point of the law at all," said an angry senator Frank Danforth (R). "The law is supposed to protect doctors and nurses who follow the teachings of God and our Lord Jesus Christ from having to treat patients who practice an unholy lifestyle."

Many doctors and nurses in Mississippi are against the law, but have expressed that they will also follow it to the letter and refuse treatment to patients who they feel are bigoted against certain lifestyle choices.

"Of course not all religious people are against LGBQT's, just like not all atheists are against religion," said Dr. Susan Jewer, atheist. "But if medical professionals are allowed to refuse care on religious grounds, I am more than happy to oblige."

To date, there have been no reports of atheist doctors or nurses taking advantage of the new law.

NEWS

Military Drawing Up Plans For Nationwide Gun Confiscations

Published 7 years ago on January 10, 2013
By G-Had

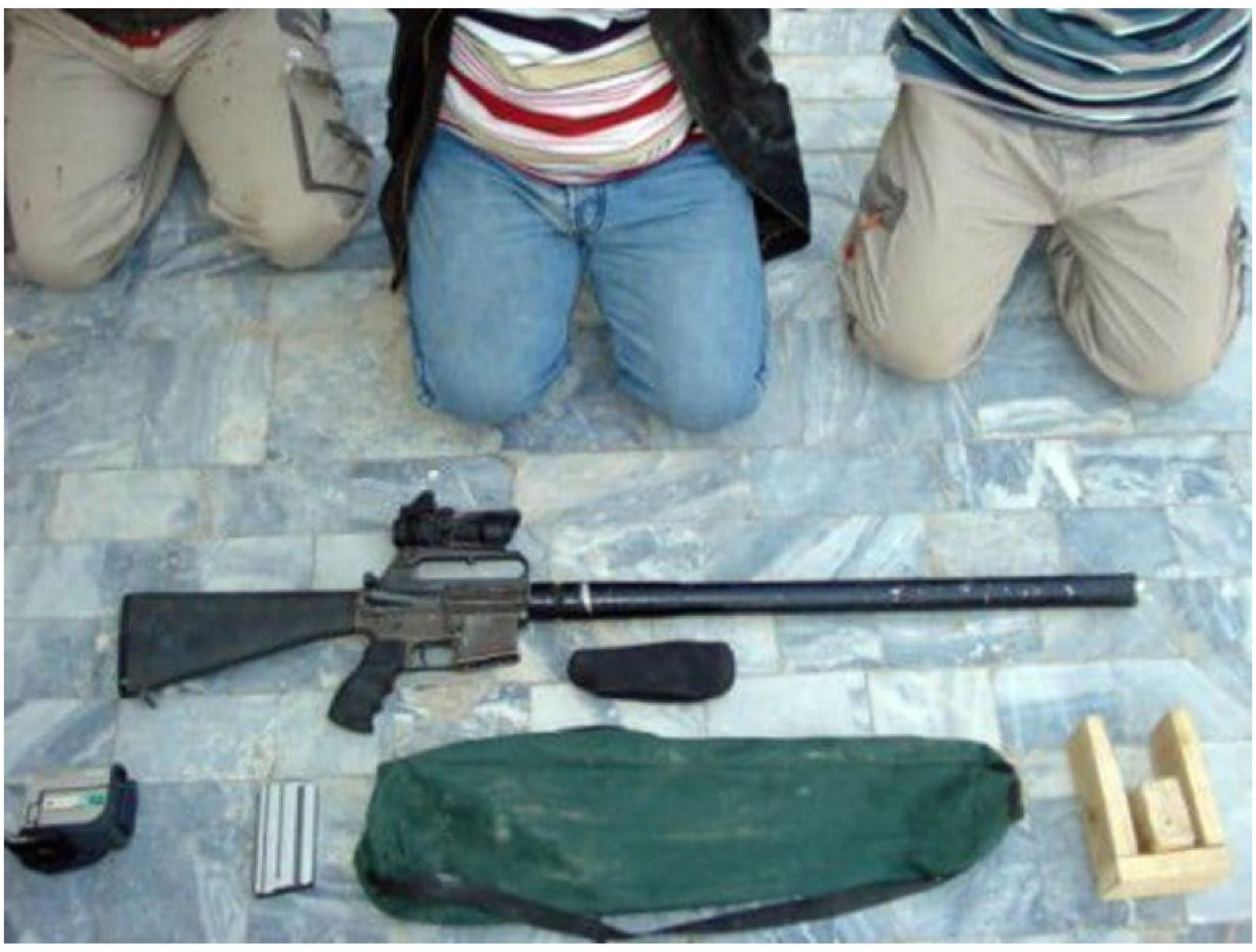

WASHINGTON, DC – A senior U.S. general has confirmed that the military has secretly drawn up plans to round up large numbers of privately-owned firearms from American gun owners.

Like 302K people like this. Be the first of your friends.

Gen. James M. Scott of the U.S. Air Force confirmed that the Pentagon received a series of formal directives from the White House between November 7 and December 13 to begin plans for a massive nationwide operation to confiscate guns using a series of federal databases compiled over the last few decades.

Scott spoke with *Duffel Blog* reporters in a parking garage in northern Virginia.

Scott also confirmed that a certain four-star general who heads the U.S. Transportation Command was intimately involved in the planning. General Scott would not reveal the general's name out of concerns for his safety.

The plan, known in the military as Operation PREAKNESS, combines a series of tactics developed for house sweeps and room clearing in Iraq and Afghanistan, which Scott admitted had been used as test-runs for the U.S.

"If we can confiscate millions of firearms in a country where we don't speak the language or understand the culture, the U.S. should be easy," Scott told *Duffel Blog*. "I just feel sorry for that poor Osama fellow we had to kill to justify the whole thing."

According to Scott, the actual planning for Operation PREAKNESS was initiated in early 2009 and developed in conjunction with the United Nations, Senator Dianne Feinstein, and several other liberal organizations such as the National Organization for Women, Planned Parenthood, Greenpeace, the American Federation of Labor, and the National Rifle Association, which is apparently a front for all the previous groups.

While there was initially some concern about the constitutionality of using the military on American soil, page 2131 of the ObamaCare Act actually amends the Posse Comitatus law to allow the military to disarm private citizens at the direction of the Secretary of Homeland Security. Objections by then-CIA Director David Petraeus were quietly silenced in November.

A test-run for PREAKNESS was actually conducted in early December in Clinger, Pennsylvania. A joint platoon of Army Rangers and UN Peacekeepers, working with select state and local officials and using imagery collected by the Google Street View Car, quietly went door-to-door and managed to collect all the firearms from Clinger owners.

The few owners who did complain were initially transported to Fort Leavenworth in Kansas to explain their case before a special international tribunal, before being sent to the National Center For Gun Control in Guantanamo Bay, Cuba.

A follow-on operation using just the UN Peacekeepers is planned later this week for any owners missed in the previous sweep, although the Peacekeepers have confirmed they will be using a post office truck to infiltrate the area.

Pope Francis Forgives 4,444 Pedophile Priests In Australia

February 27, 2018 Baxter Dmitry

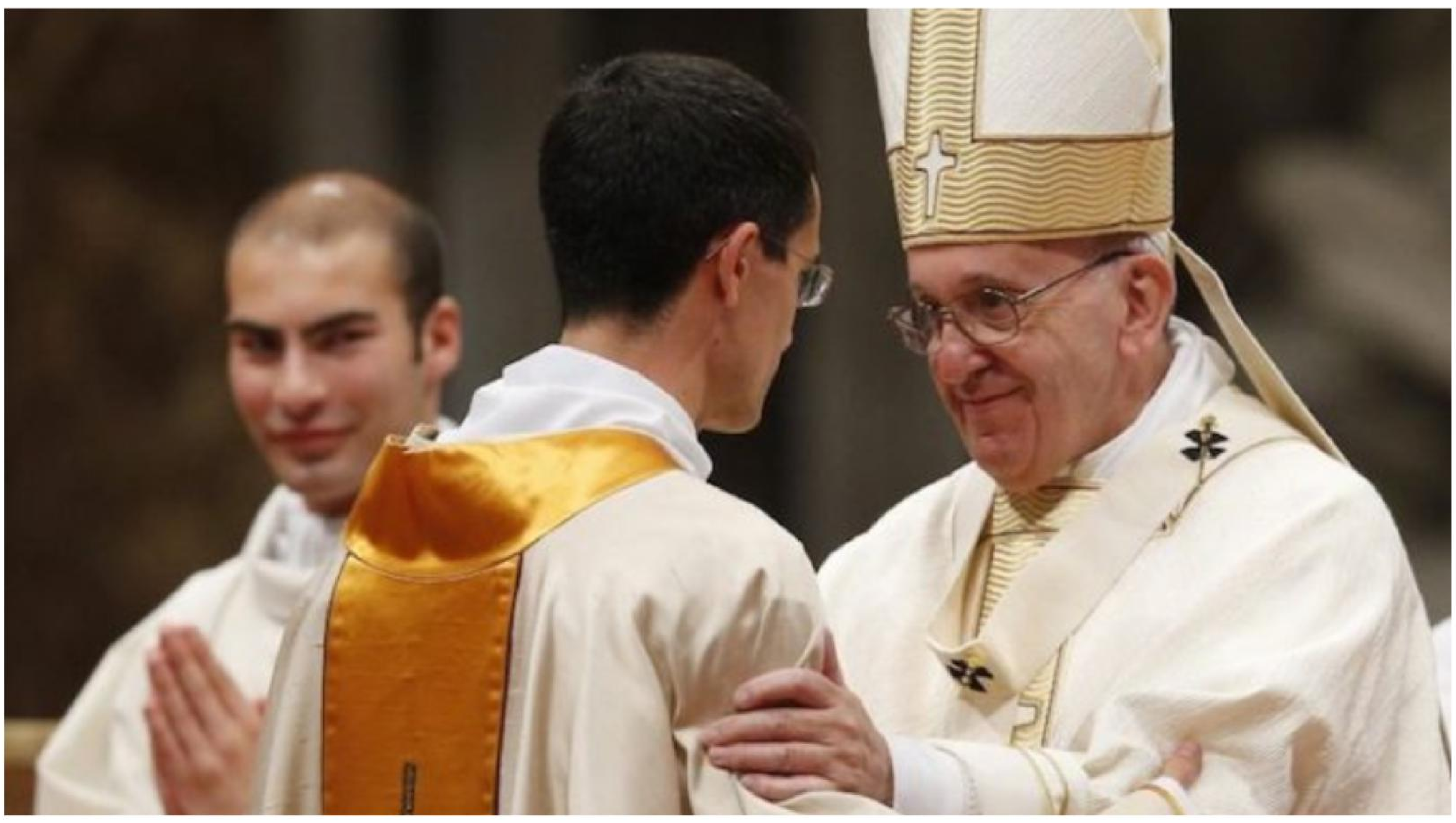

An inquiry in Sydney has uncovered evidence that Pope Francis has forgiven and covered up the crimes of 4,444 pedophile priests in Australia.

The Austrailian commission reported that not only were the priests forgiven by the Vatican for their crimes, allegations were "*swept under the carpet*" and never reported to law enforcement.

Following years of mounting pressure to look into the claims of pedophilia, Canberra finally set up the Royal Commission into Institutional Responses to Child Sexual Abuse, and the extent of the Catholic Church's cover up was astonishing.

During their investigation, the panel also discovered a pattern of witness intimidation and cover-ups. Gail Furness, the lawyer in charge of questioning at the Sydney inquiry, said in a statement:

> *"Between 1950 and 2010, overall seven percent of priests were alleged perpetrators,"*
>
> *"The accounts were depressingly similar. Children were ignored or worse, punished. Allegations were not investigated. Priests and religious (figures) were moved."*
>
> *"The parishes or communities to which they were moved knew nothing of their past. Documents were not kept or they were destroyed. Secrecy prevailed as did cover ups."*

Inquiry implicates Vatican finance chief

DW reports: The claims of a cover-up go so high that they have implicated Australia's top Catholic clergyman, George Pell.

Pell, who is now the Vatican finance chief, was questioned over his handling of abuse claims in the state of Victoria in the 1970s.

Cardinal Pell recently returned to Australia to face charges of child abuse and is the highest-ranking official in the Catholic Chruch to ever go on trial for pedophilia.

The inquiry found that about 90 percent of the 1,880 alleged perpetrators were men, and the victims averaged around age 10 for girls and 11 for boys.

> *"These numbers are shocking, they are tragic, they are indefensible," said Francis Sullivan, the head of the church's Truth, Justice and Healing Council.*
>
> *"This data, along with all we have heard over the past four years, can only be interpreted for what it is: a massive failure on the part of the Catholic Church in Australia to protect children from abusers. "As Catholics, we hang our heads in shame."*

Of the 300 cases referred to police throughout the course of the four-year investigation, only 27 prosecutions have been able to move forward, with 75 more pending.

The New York Times

NEWS

BREAKING: Black Lives Matter Leader Kept 'Virtually All' Donations

13.2k SHARES Share Tweet

You are surely familiar with the international activist movement which originated in the African-American community, under the name of Black Lives Matter. The organization advocates against violence and racism towards black people in society.

Not that long ago, the liberal media pushed an "inspiring" story of a homeless black woman who became one of the leading figures in the movement, and was an important member of Black Lives Matter, organizing over 900 events and gather millions of dollars in donations, which were allegedly for a good cause. These donations were supposed to help "raise black men and women" up in society.

However, newest revelations suggest that Marquesha Johnson was not the humanitarian everybody thought she was. In fact, the donations she collected helped only herself to "rise" in society.

A class action lawsuit, stated that Johnson had "solicited donations from vulnerable people to help others but instead used it to help herself."

According to the document, the Black Lives Matter leading figure, purchased a $1.2 million home for herself. But this is not all, instead of using the donations to help people she also bought herself a brand new Range rover as well as some "other exorbitancies".

Nevertheless, sadly, she will get away with it because it's most likely that the way she did it was perfectly legal. Namely, she used the GoFundMe campaign to ask for the funds and reportedly, the company is particularly bad at working with police to crack down on scammers.

Allegedly, Marquesha Johnson was interrogated by the police, but she denied all accusations about misusing the funds, and she refused to submit any records.

She used the company platform in order to fill her pockets with money that weren't hers to take. This is more than just bad publicity for Black Lives Matter, to have someone like this in their leading circles while blacks across America continue to suffer poverty, and gang culture.

The Washington Post
Democracy Dies in Darkness

STUDENT SUSPENDED FOR PRETENDING TO BE TRANSGENDER JUST TO USE GIRL SHOWERS DURING GYM CLASS

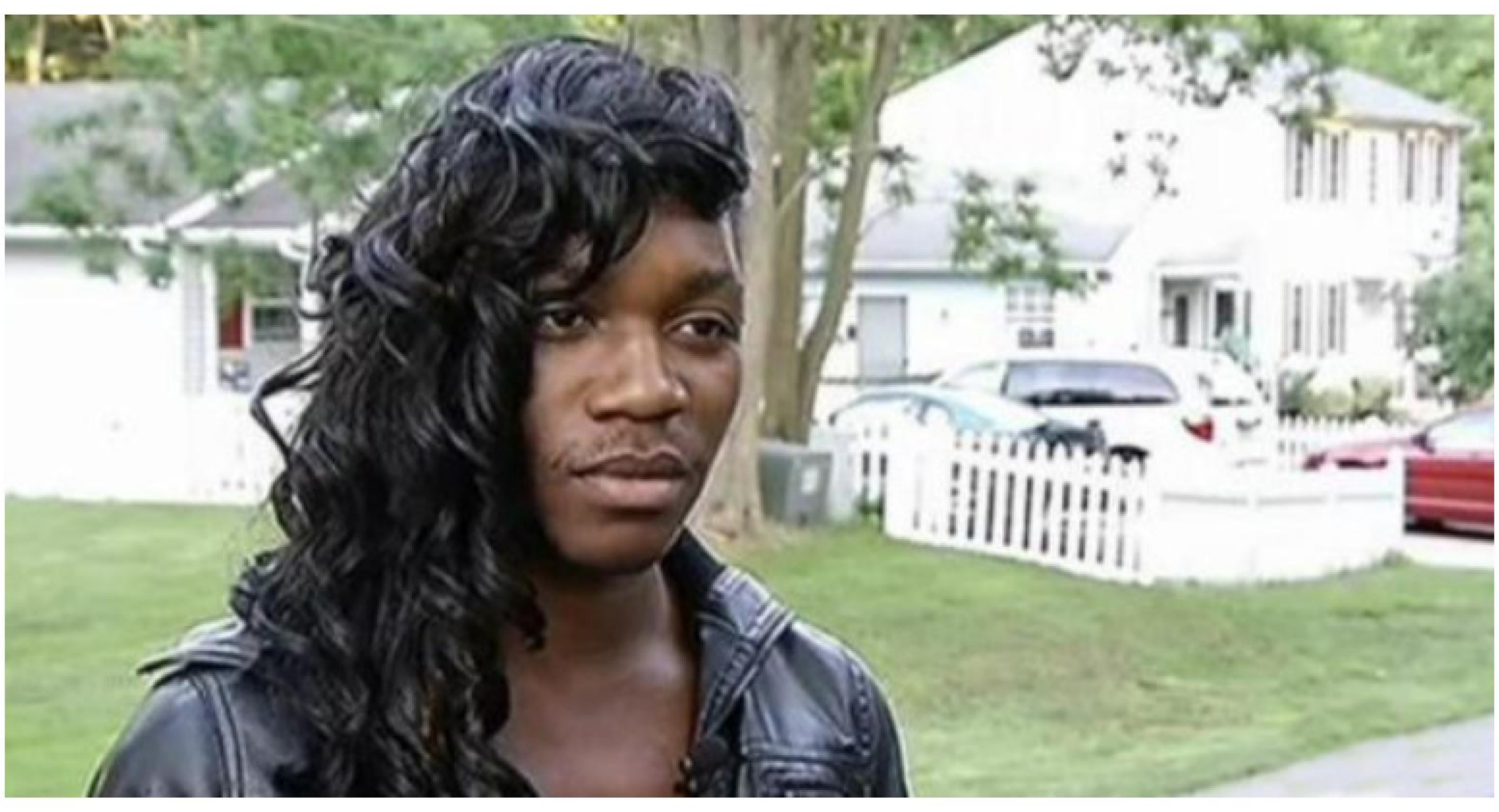

Hornwood, SC | A transgender male student has been suspended indefinitely after being accused of pretending to be transgender as an excuse to use the girl's shower during gym class.

Soccamee high school officials were alarmed by the student's indecent behavior after several students and parents lodged complaints and reported the "immoral behavior" to school administrators.

The transgender male student was recently allowed by school officials to use all female washrooms, including having access to the girl's locker room, after he had asked to be treated as a biological female.

The transgender male student also allegedly bragged about how the school would let him take showers with the girls just because he "wore a wig" and "painted his nails" according to several students.

> *"He always had this huge erection while we were in the shower, I never understood why the school allowed a boy to be in the girls' shower with us," one female student told local reporters.*

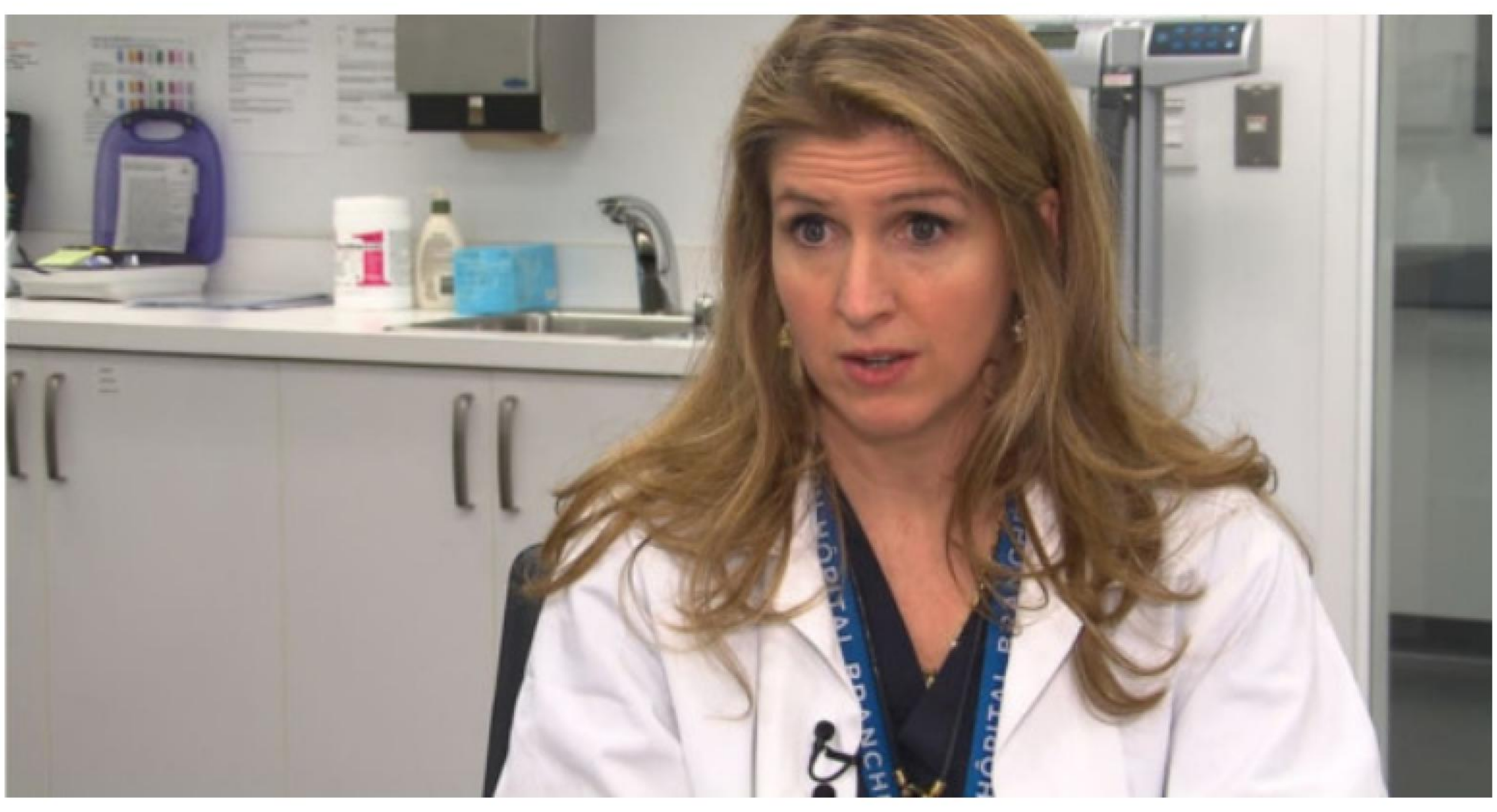

Soccamee high school nurse, Helena Ferguson, has already reported that three female students claim they have been impregnated by the transgender male student but believes others could follow.

Inappropriate behavior

Some students even reported being inappropriately touched by the transgender male student in the shower after gym class.

> *"He told me that if I didn't wash my vagina properly I could catch rabies and started to show me how by washing the inside of my vagina with his two hands and a bar of soap," one student admitted.*

Several female students admitted having consensual sexual relationships with the transgender male student but others described him as a "sexual predator."

> *"He once tried to put his dick in my asshole while I was bending over and pretended he had slipped in the shower," added another.*

Orangeburg County Sheriff's Office has opened a formal investigation into the allegations and the transgender male student, who is currently suspended indefinitely from attending Soccamee high school, is temporarily not allowed to enter the school perimeter or come in contact with fellow classmates until further notice.

US NEWS, USA

California Gov. Jerry Brown To Force Schools To Show Kids 'Gay Sex'

Date: December 31, 2017
Author: Nwo Report

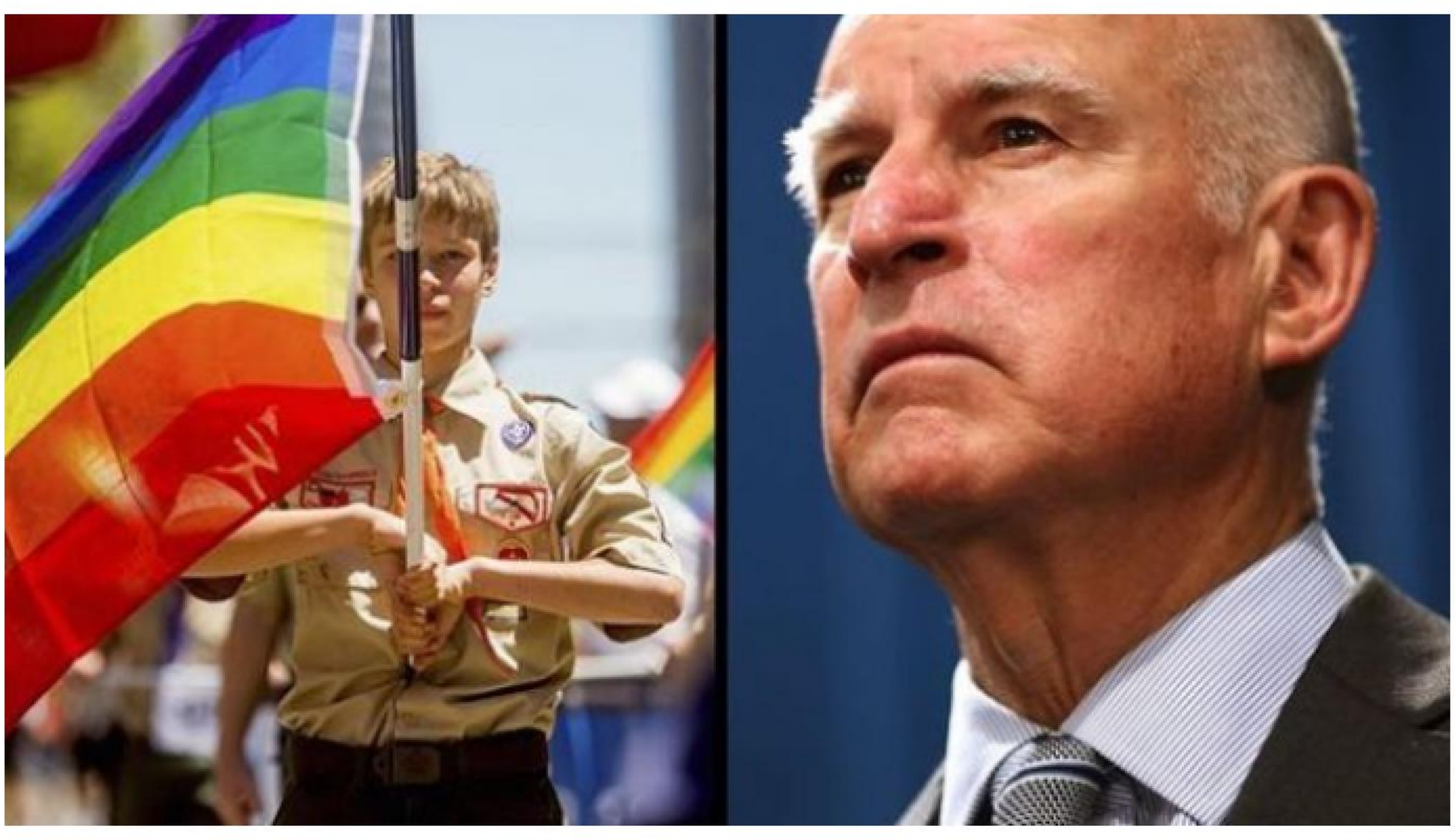

California Governor Jerry Brown has mandated the use of gay pornography in elementary schools in order to teach children about LGBT sex.

This makes California the first state in the union to not only use mandatory LGBT-inclusive textbooks in schools, but also force young children to learn sexually explicit material without the choice of opting out.

The textbooks will include references to famous gay celebrities and public figures, as well as detail the various sexual choices a gay person has.

Truthrevolt.org reports: That law requires a "fair, accurate, inclusive, and respectful" treatment of homosexual, bisexual, transgender, and lesbian Americans despite the historical insignificance.

"We're not trying to make anybody gay; we're not saying there's an agenda; we're not saying that these people are better than other people; what we're saying is this is another group of Americans and they face certain prejudices," said state publisher Mark Jarrett, whose history textbooks include special mention of the sexual preferences of historical figures like Jane Adams, Emily Dickinson, Nathaniel Hawthorne, President James Buchanan, though it's inconclusive if these prominent Americans were gay or not.

"I think we should say, 'Buchanan, he never married. He had a very good friend who was living with him. He may have been gay,'" Jarrett added. "On the other hand, at that time, being gay was seen as something evil and wrong."

California approved the textbooks written by Jarrett, a Ph.D, to be used in the 8th grade.

Students will read that legendary stagecoach driver Charlie Parkhurst "was a woman who identified as a man," according to Fox News.

They will read that George Washington's chief of staff Baron Von Steuben "may have been gay," and that poet Walt Whitman "was drawn to young men... but denied his same sex preferences in public."

Critics say much of these accounts are speculation, but California rejected any publisher that didn't mention the homosexual preferences or claims against historical figures in their textbooks.

In one textbook, the state forced the publisher to add "lesbian" to describe NASA astronaut Sally Ride.

And here we thought the Democratic Party was the party of "choice." Clearly the state knows better than the parents what children need to learn.

Fake News Articles with Non-Credible News Source

News

New law makes it legal for atheist doctors and nurses to refuse care to religious patients

By SP Team on January 19, 2018

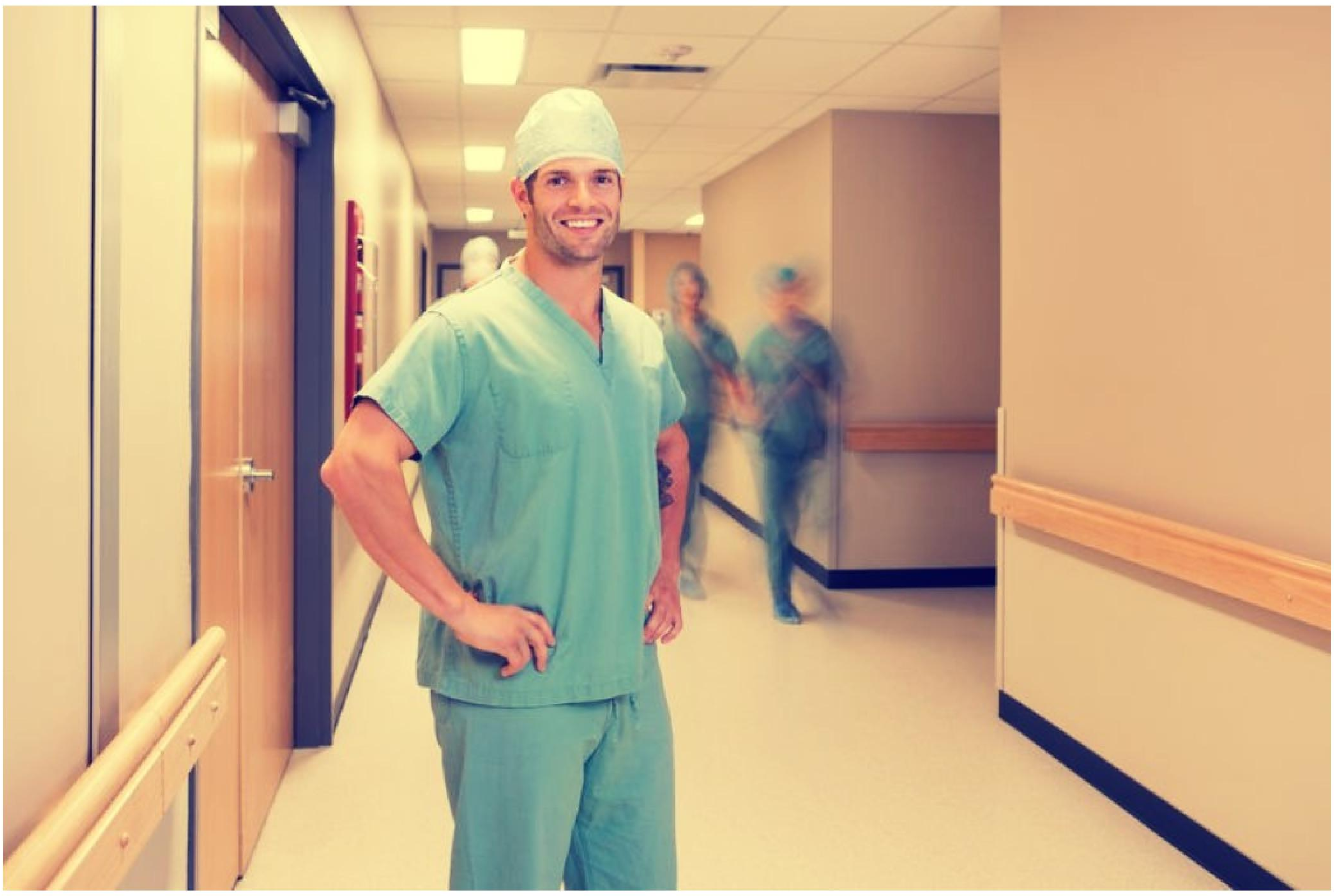

170.3k
SHARES

Share Tweet

JACKSON, MS – A new law in Mississippi has made it legal for doctors and nurses to refuse care to certain patients on religious grounds .

The law allows medical staff to refuse treatment to members of the LGBQT community on the grounds that it violates their religious beliefs. It would serve to reason this would also pertain to atheist doctors and nurses who could refuse to treat patients who are religious.

"No, no, no. This isn't the point of the law at all," said an angry senator Frank Danforth (R). "The law is supposed to protect doctors and nurses who follow the teachings of God and our Lord Jesus Christ from having to treat patients who practice an unholy lifestyle."

Many doctors and nurses in Mississippi are against the law, but have expressed that they will also follow it to the letter and refuse treatment to patients who they feel are bigoted against certain lifestyle choices.

"Of course not all religious people are against LGBQT's, just like not all atheists are against religion," said Dr. Susan Jewer, atheist. "But if medical professionals are allowed to refuse care on religious grounds, I am more than happy to oblige."

To date, there have been no reports of atheist doctors or nurses taking advantage of the new law.

NEWS

Military Drawing Up Plans For Nationwide Gun Confiscations

Published 7 years ago on January 10, 2013
By G-Had

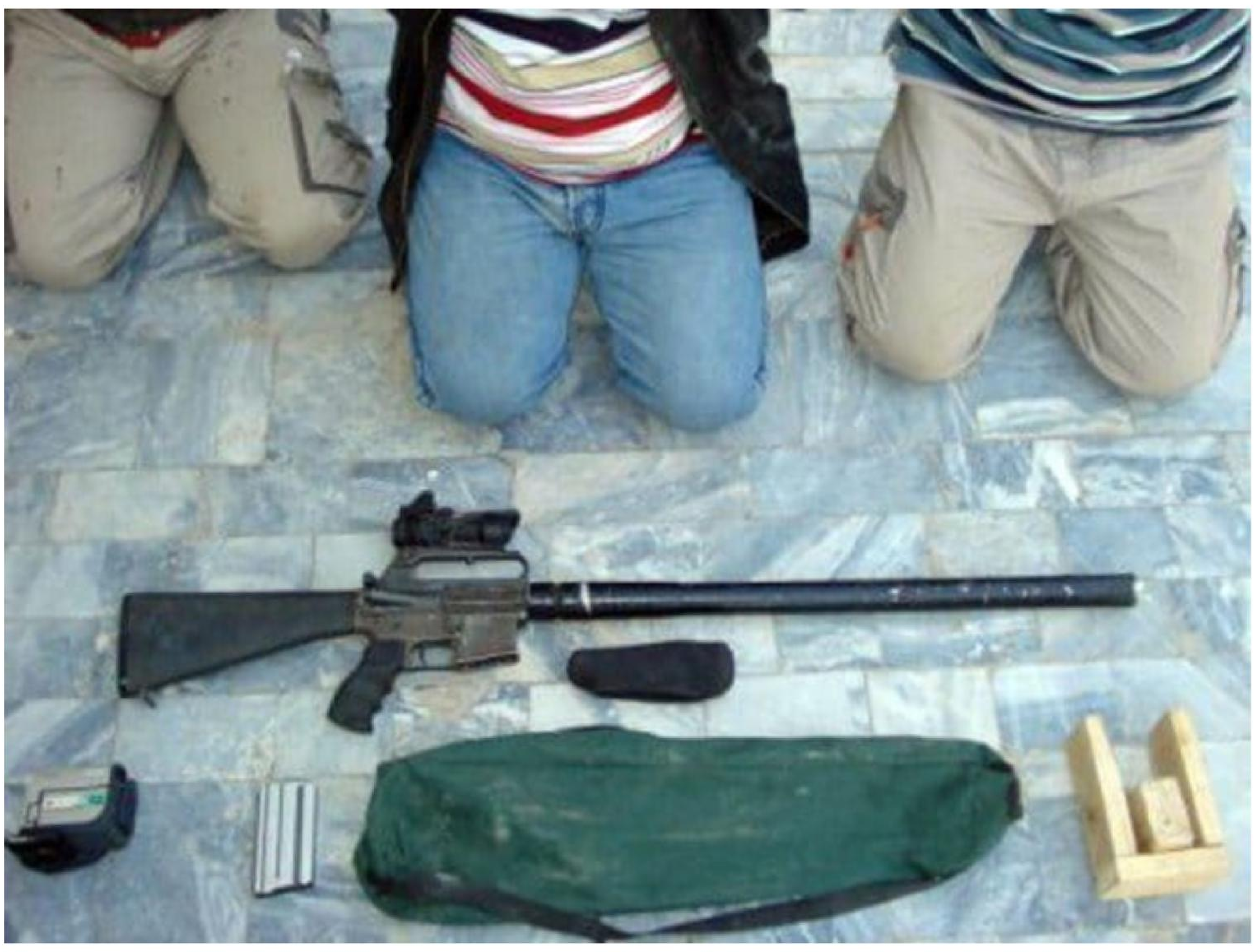

WASHINGTON, DC – A senior U.S. general has confirmed that the military has secretly drawn up plans to round up large numbers of privately-owned firearms from American gun owners.

Like 302K people like this. Be the first of your friends.

Gen. James M. Scott of the U.S. Air Force confirmed that the Pentagon received a series of formal directives from the White House between November 7 and December 13 to begin plans for a massive nationwide operation to confiscate guns using a series of federal databases compiled over the last few decades.

Scott spoke with *Duffel Blog* reporters in a parking garage in northern Virginia.

Scott also confirmed that a certain four-star general who heads the U.S. Transportation Command was intimately involved in the planning. General Scott would not reveal the general's name out of concerns for his safety.

The plan, known in the military as Operation PREAKNESS, combines a series of tactics developed for house sweeps and room clearing in Iraq and Afghanistan, which Scott admitted had been used as test-runs for the U.S.

"If we can confiscate millions of firearms in a country where we don't speak the language or understand the culture, the U.S. should be easy," Scott told *Duffel Blog*. "I just feel sorry for that poor Osama fellow we had to kill to justify the whole thing."

According to Scott, the actual planning for Operation PREAKNESS was initiated in early 2009 and developed in conjunction with the United Nations, Senator Dianne Feinstein, and several other liberal organizations such as the National Organization for Women, Planned Parenthood, Greenpeace, the American Federation of Labor, and the National Rifle Association, which is apparently a front for all the previous groups.

While there was initially some concern about the constitutionality of using the military on American soil, page 2131 of the ObamaCare Act actually amends the Posse Comitatus law to allow the military to disarm private citizens at the direction of the Secretary of Homeland Security. Objections by then-CIA Director David Petraeus were quietly silenced in November.

A test-run for PREAKNESS was actually conducted in early December in Clinger, Pennsylvania. A joint platoon of Army Rangers and UN Peacekeepers, working with select state and local officials and using imagery collected by the Google Street View Car, quietly went door-to-door and managed to collect all the firearms from Clinger owners.

The few owners who did complain were initially transported to Fort Leavenworth in Kansas to explain their case before a special international tribunal, before being sent to the National Center For Gun Control in Guantanamo Bay, Cuba.

A follow-on operation using just the UN Peacekeepers is planned later this week for any owners missed in the previous sweep, although the Peacekeepers have confirmed they will be using a post office truck to infiltrate the area.

Pope Francis Forgives 4,444 Pedophile Priests In Australia

February 27, 2018 Baxter Dmitry

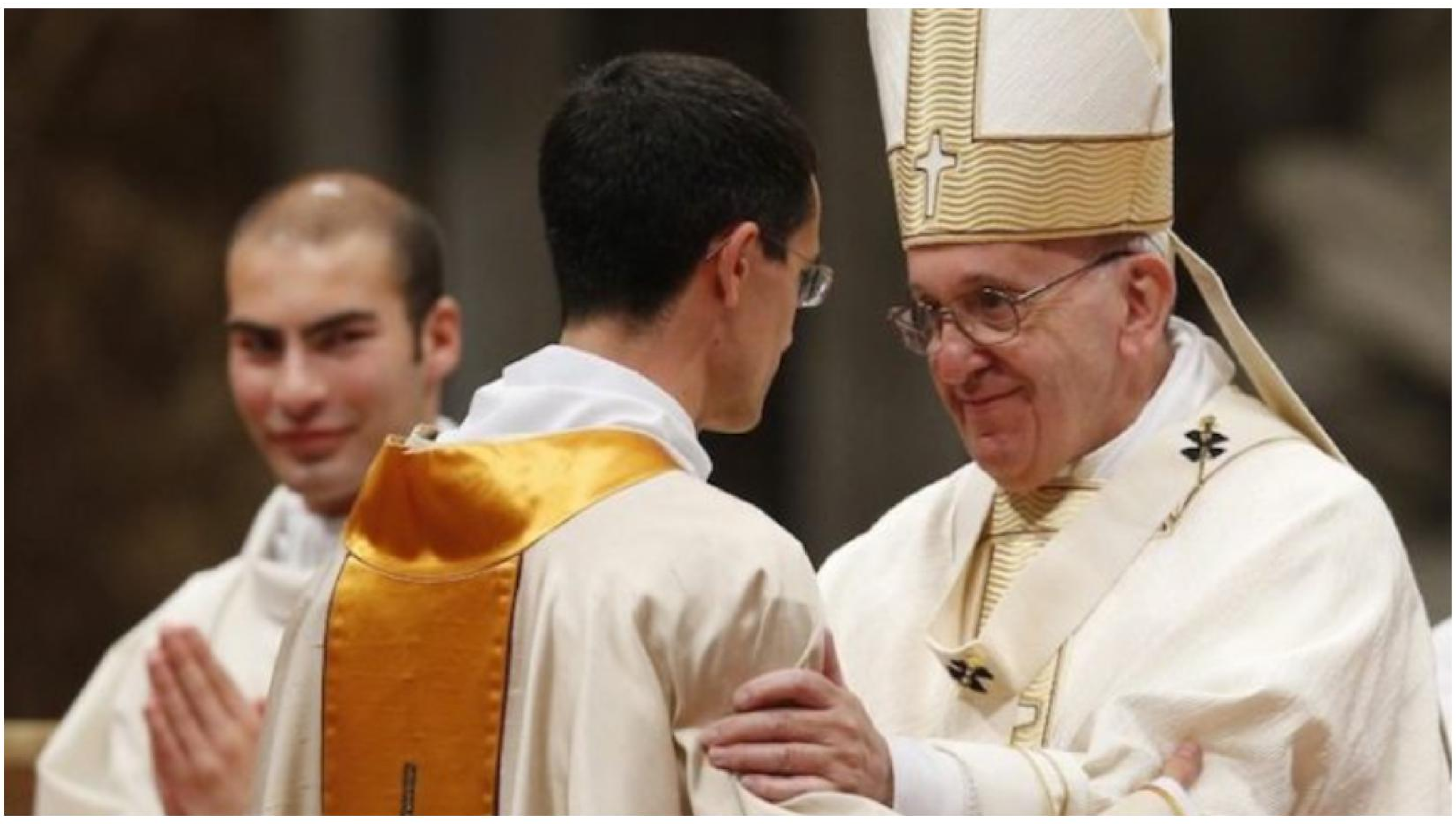

An inquiry in Sydney has uncovered evidence that Pope Francis has forgiven and covered up the crimes of 4,444 pedophile priests in Australia.

The Austrailian commission reported that not only were the priests forgiven by the Vatican for their crimes, allegations were "*swept under the carpet*" and never reported to law enforcement.

Following years of mounting pressure to look into the claims of pedophilia, Canberra finally set up the Royal Commission into Institutional Responses to Child Sexual Abuse, and the extent of the Catholic Church's cover up was astonishing.

During their investigation, the panel also discovered a pattern of witness intimidation and cover-ups. Gail Furness, the lawyer in charge of questioning at the Sydney inquiry, said in a statement:

> *"Between 1950 and 2010, overall seven percent of priests were alleged perpetrators,"*
>
> *"The accounts were depressingly similar. Children were ignored or worse, punished. Allegations were not investigated. Priests and religious (figures) were moved."*
>
> *"The parishes or communities to which they were moved knew nothing of their past. Documents were not kept or they were destroyed. Secrecy prevailed as did cover ups."*

Inquiry implicates Vatican finance chief

DW reports: The claims of a cover-up go so high that they have implicated Australia's top Catholic clergyman, George Pell.

Pell, who is now the Vatican finance chief, was questioned over his handling of abuse claims in the state of Victoria in the 1970s.

Cardinal Pell recently returned to Australia to face charges of child abuse and is the highest-ranking official in the Catholic Chruch to ever go on trial for pedophilia.

The inquiry found that about 90 percent of the 1,880 alleged perpetrators were men, and the victims averaged around age 10 for girls and 11 for boys.

> *"These numbers are shocking, they are tragic, they are indefensible," said Francis Sullivan, the head of the church's Truth, Justice and Healing Council.*
>
> *"This data, along with all we have heard over the past four years, can only be interpreted for what it is: a massive failure on the part of the Catholic Church in Australia to protect children from abusers. "As Catholics, we hang our heads in shame."*

Of the 300 cases referred to police throughout the course of the four-year investigation, only 27 prosecutions have been able to move forward, with 75 more pending.

NEWS

BREAKING: Black Lives Matter Leader Kept ‘Virtually All’ Donations

13.2k SHARES Share Tweet

You are surely familiar with the international activist movement which originated in the African-American community, under the name of Black Lives Matter. The organization advocates against violence and racism towards black people in society.

Not that long ago, the liberal media pushed an “inspiring” story of a homeless black woman who became one of the leading figures in the movement, and was an important member of Black Lives Matter, organizing over 900 events and gather millions of dollars in donations, which were allegedly for a good cause. These donations were supposed to help “raise black men and women” up in society.

However, newest revelations suggest that Marquesha Johnson was not the humanitarian everybody thought she was. In fact, the donations she collected helped only herself to “rise” in society.
A class action lawsuit, stated that Johnson had “solicited donations from vulnerable people to help others but instead used it to help herself.”

According to the document, the Black Lives Matter leading figure, purchased a $1.2 million home for herself. But this is not all, instead of using the donations to help people she also bought herself a brand new Range rover as well as some “other exorbitancies”.

Nevertheless, sadly, she will get away with it because it’s most likely that the way she did it was perfectly legal. Namely, she used the GoFundMe campaign to ask for the funds and reportedly, the company is particularly bad at working with police to crack down on scammers.

Allegedly, Marquesha Johnson was interrogated by the police, but she denied all accusations about misusing the funds, and she refused to submit any records.

She used the company platform in order to fill her pockets with money that weren’t hers to take. This is more than just bad publicity for Black Lives Matter, to have someone like this in their leading circles while blacks across America continue to suffer poverty, and gang culture.

STUDENT SUSPENDED FOR PRETENDING TO BE TRANSGENDER JUST TO USE GIRL SHOWERS DURING GYM CLASS

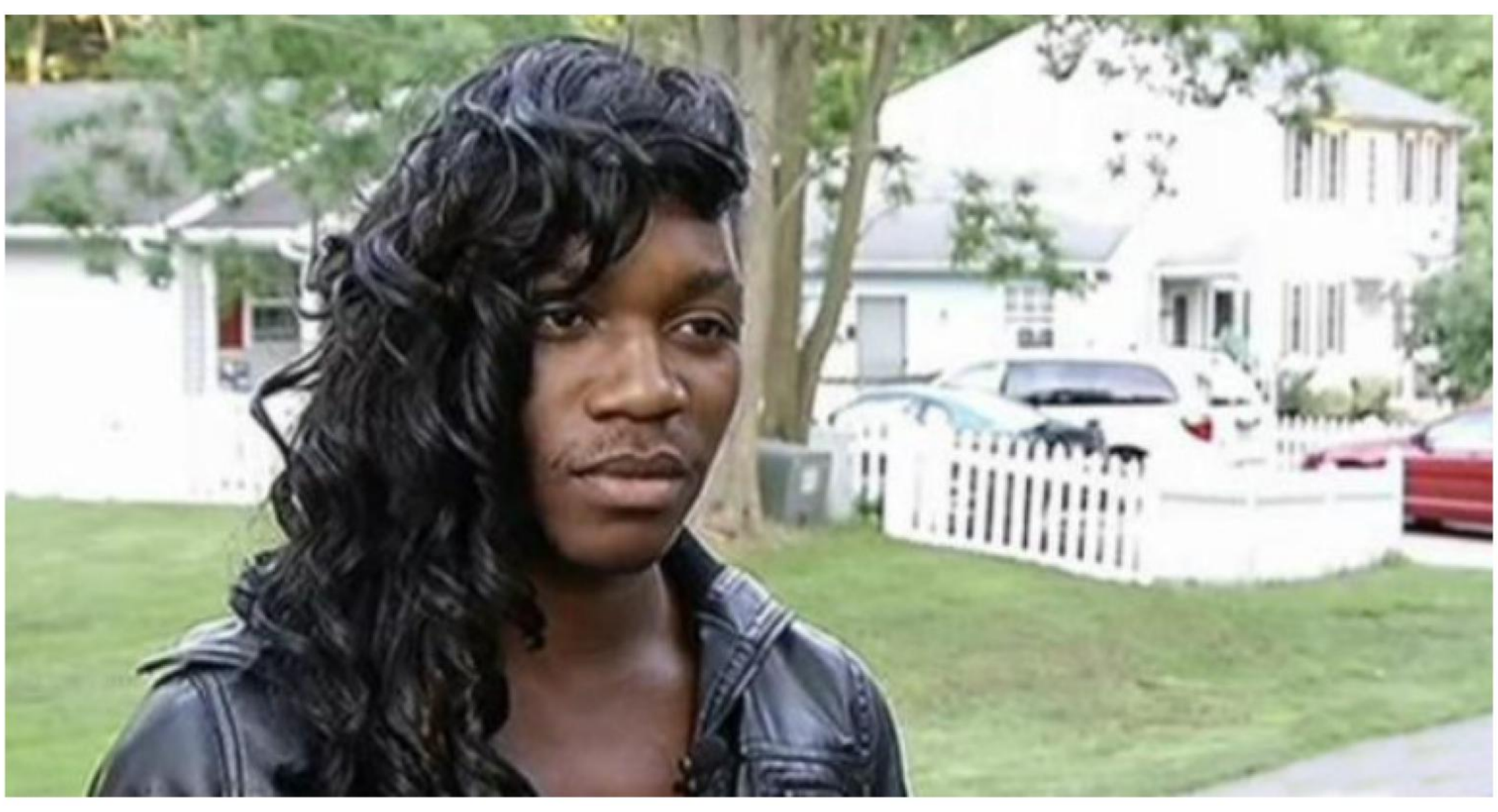

Hornwood, SC | A transgender male student has been suspended indefinitely after being accused of pretending to be transgender as an excuse to use the girl's shower during gym class.

Soccamee high school officials were alarmed by the student's indecent behavior after several students and parents lodged complaints and reported the "immoral behavior" to school administrators.

The transgender male student was recently allowed by school officials to use all female washrooms, including having access to the girl's locker room, after he had asked to be treated as a biological female.

The transgender male student also allegedly bragged about how the school would let him take showers with the girls just because he "wore a wig" and "painted his nails" according to several students.

> *"He always had this huge erection while we were in the shower, I never understood why the school allowed a boy to be in the girls' shower with us," one female student told local reporters.*

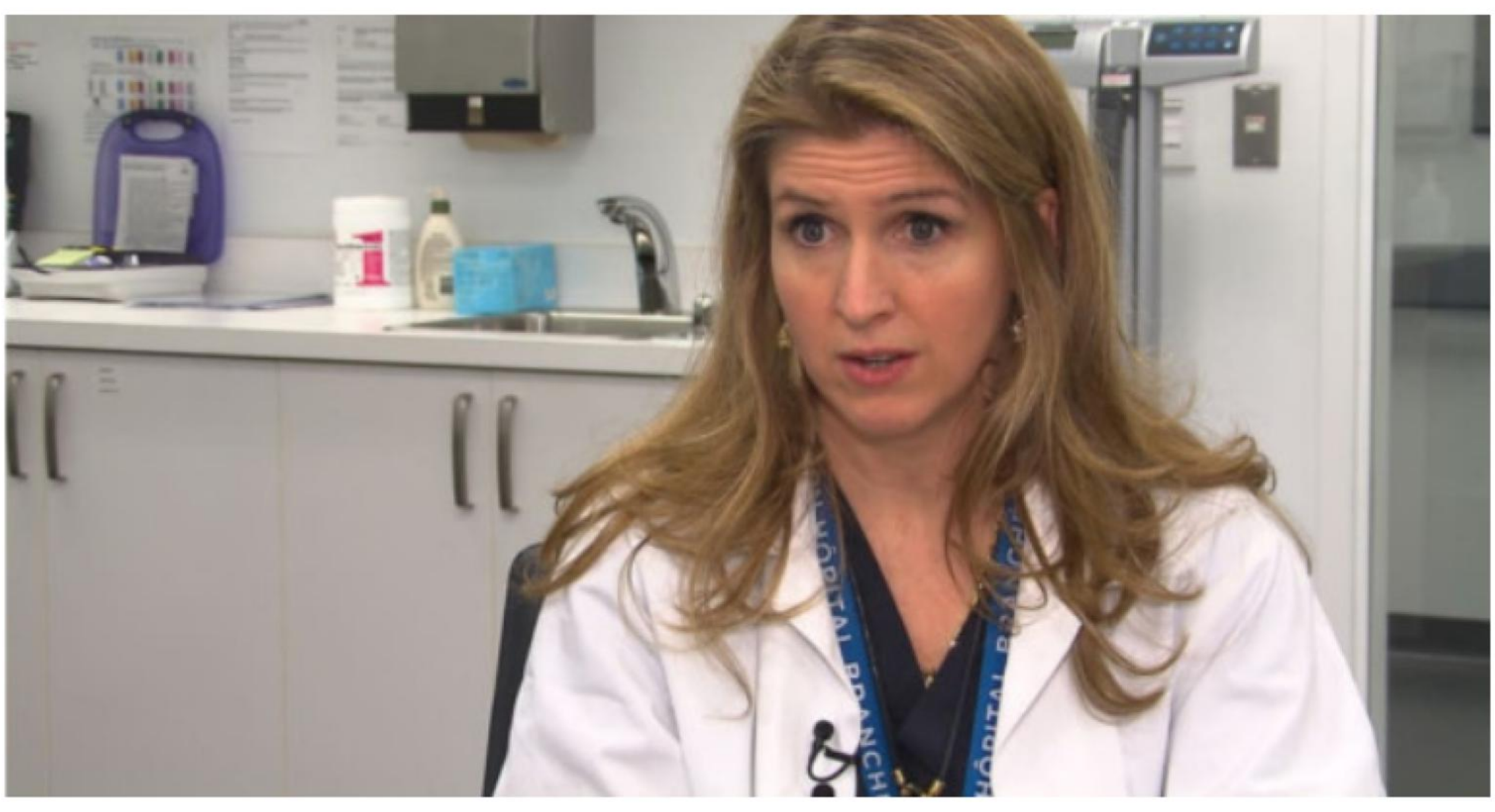

Soccamee high school nurse, Helena Ferguson, has already reported that three female students claim they have been impregnated by the transgender male student but believes others could follow.

Inappropriate behavior

Some students even reported being inappropriately touched by the transgender male student in the shower after gym class.

> *"He told me that if I didn't wash my vagina properly I could catch rabies and started to show me how by washing the inside of my vagina with his two hands and a bar of soap," one student admitted.*

Several female students admitted having consensual sexual relationships with the transgender male student but others described him as a "sexual predator."

> *"He once tried to put his dick in my asshole while I was bending over and pretended he had slipped in the shower," added another.*

Orangeburg County Sheriff's Office has opened a formal investigation into the allegations and the transgender male student, who is currently suspended indefinitely from attending Soccamee high school, is temporarily not allowed to enter the school perimeter or come in contact with fellow classmates until further notice.

California Gov. Jerry Brown To Force Schools To Show Kids 'Gay Sex'

Date: December 31, 2017
Author: Nwo Report

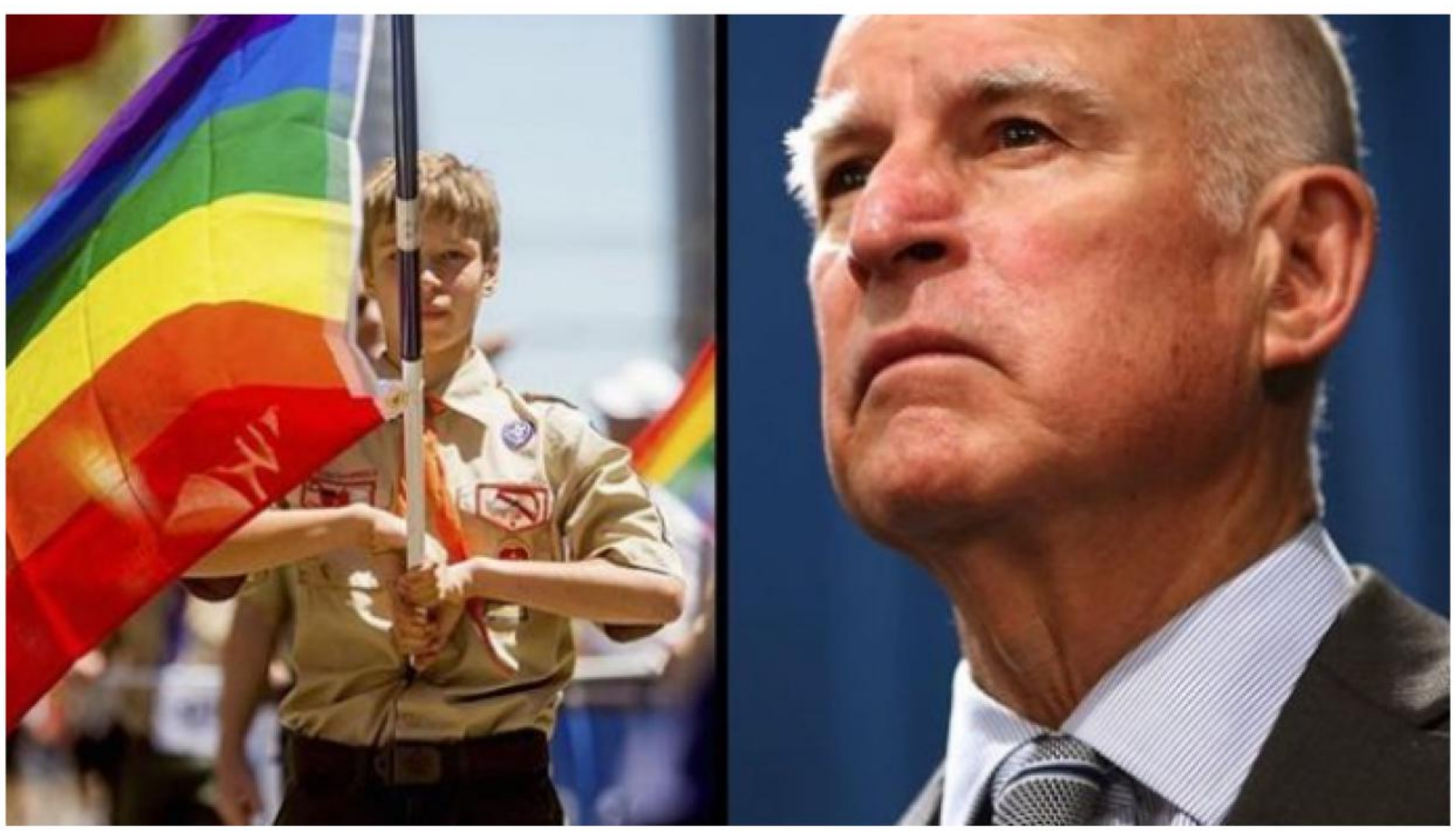

California Governor Jerry Brown has mandated the use of gay pornography in elementary schools in order to teach children about LGBT sex.

This makes California the first state in the union to not only use mandatory LGBT-inclusive textbooks in schools, but also force young children to learn sexually explicit material without the choice of opting out.

The textbooks will include references to famous gay celebrities and public figures, as well as detail the various sexual choices a gay person has.

Truthrevolt.org reports: That law requires a "fair, accurate, inclusive, and respectful" treatment of homosexual, bisexual, transgender, and lesbian Americans despite the historical insignificance.

"We're not trying to make anybody gay; we're not saying there's an agenda; we're not saying that these people are better than other people; what we're saying is this is another group of Americans and they face certain prejudices," said state publisher Mark Jarrett, whose history textbooks include special mention of the sexual preferences of historical figures like Jane Adams, Emily Dickinson, Nathaniel Hawthorne, President James Buchanan, though it's inconclusive if these prominent Americans were gay or not.

"I think we should say, 'Buchanan, he never married. He had a very good friend who was living with him. He may have been gay,'" Jarrett added. "On the other hand, at that time, being gay was seen as something evil and wrong."

California approved the textbooks written by Jarrett, a Ph.D, to be used in the 8th grade.

Students will read that legendary stagecoach driver Charlie Parkhurst "was a woman who identified as a man," according to Fox News.

They will read that George Washington's chief of staff Baron Von Steuben "may have been gay," and that poet Walt Whitman "was drawn to young men… but denied his same sex preferences in public."

Critics say much of these accounts are speculation, but California rejected any publisher that didn't mention the homosexual preferences or claims against historical figures in their textbooks.

In one textbook, the state forced the publisher to add "lesbian" to describe NASA astronaut Sally Ride.

And here we thought the Democratic Party was the party of "choice." Clearly the state knows better than the parents what children need to learn.
