# Supplementary material for: The role of analytical reasoning and source credibility on the evaluation of real and fake full-length news articles
Source: Cogn Res Princ Implic. 2021 Mar 31;6:24. doi: 10.1186/s41235-021-00292-3 (PMC8012428; doi:10.1186/s41235-021-00292-3)
Supplement: Supplementary file 2 — Additional file 2. Appendix B: Confidence ratings and sharing responses for the news articles. [file 41235_2021_292_MOESM2_ESM.docx]

**Appendix B**

**Confidence Ratings**

The findings for confidence ratings paralleled those for accuracy judgments. In particular, the two-way interaction between Veracity x CRT was significant in both studies [Study 1: *χ*^2^ _(1)_ = 36, *p* < 0.001; Study 2: *χ*^2^ _(1)_ = 10.63, *p* = 0.001]. Confidence in accurate detection of real news was not associated with analytical reasoning (indexed by CRT scores) [Study 1: *z* = 2.16, *p* = 0.061; Study 2: *z* = 1.57, *p* = 0.116]. Confidence in accurate detection of fake news, in contrast, increased with higher analytical reasoning (i.e., greater CRT scores) [Study 1: *z* = 2.96, *p* = 0.006; Study 2: *z* = 2.32, *p* = 0.009].

**News Sharing Responses**

News sharing responses were not analyzed further due to a floor effect in “*yes*” responses in both studies (Table B1).

Table B1

*Percent for responding “yes” for sharing real and fake news respectively in Study 1 and Study 2*

|  | Study 1 | Study 2 |
| --- | --- | --- |
| Real news | 1.8% | 2.1% |
| Fake news | 0.5% | 0.7% |
